# Efficacy, safety, and immunogenicity of SARS-CoV-2 mRNA vaccine (Omicron BA.5) LVRNA012: a randomized, double-blind, placebo-controlled phase 3 trial

**DOI:** 10.3389/fimmu.2024.1407826

**Published:** 2024-06-06

**Authors:** Huan Zhou, Hui Zheng, Yucai Peng, Yue Su, Xuya Yu, Weixiao Wang, Simin Li, Yuzhou Ding, Shiping Jiao, Ying Wang, Xingyu Zhu, Liping Luo, Ziyong Dong, Lu Liu, Fan Zhang, Qiang Wu, Jingxin Li, Fengcai Zhu

**Affiliations:** ^1^Clinical Trial Center, The First Affiliated Hospital of Bengbu Medical College, Bengbu, Anhui, China; ^2^School of Clinical Trial Technology, Anqing Medical College, Anqing, Anhui, China; ^3^Key Laboratory of Innovative Pharmaceutical Research and Clinical Evaluation Jointly Constructed by Anhui, Bengbu Medical College, Bengbu, Anhui, China; ^4^Key Laboratory of Environmental Medicine Engineering, Ministry of Education, School of Public Health, Southeast University, Nanjing, Jiangsu, China; ^5^Liverna Therapeutics Inc, Zhuhai, China; ^6^AIM Vaccine Co. Ltd., Beijing, China; ^7^School of Population Medicine and Public Health, Chinese Academy of Medical Sciences and Peking Union Medical College, Beijing, China; ^8^School of Public Health, Nanjing Medical University, Nanjing, Jiangsu, China; ^9^NHC Key Laboratory of Enteric Pathogenic Microbiology, Jiangsu Provincial Center for Disease Control and Prevention, Nanjing, Jiangsu, China; ^10^Institute of Global Public Health and Emergency Pharmacy, China Pharmaceutical University, Nanjing, Jiangsu, China

**Keywords:** COVID-19, mRNA vaccine, booster dose, heterologous immunization, inactivated vaccine

## Abstract

**Background:**

We aimed to evaluate the efficacy, safety, and immunogenicity of a SARS-CoV-2 mRNA vaccine (Omicron BA.5) LVRNA012 given as the booster in immunized but SARS-CoV-2 infection-free adults in China.

**Methods:**

This is a single-center, randomized, double-blind, placebo-controlled phase 3 clinical trial enrolling healthy adult participants (≥18 years) who had completed two or three doses of inactivated COVID-19 vaccines at least 6 months before, in Bengbu, Anhui province, China. Eligible participants were randomly assigned (1:1) to receive a booster intramuscular vaccination with an LVRNA012 vaccine (100ug) or placebo. The primary endpoint was the protective efficacy of a booster dose of the LVRNA012 vaccine or placebo against symptomatic COVID-19 of any severity 14 days after vaccination. Laboratory-confirmed COVID-19 infections were identified from 14 days to 180 days after intervention, with active surveillance for symptomatic illness 8 times per month between 7 to 90 days and at least once per month between 90 to 180 days after intervention.

**Results:**

2615 participants were recruited and randomly assigned in a 1:1 ratio to either the vaccine group (1308) or the placebo group (1307). A total of 141 individuals (46 in the LVRNA012 group and 95 in the placebo group) developed symptomatic COVID-19 infection 14 days after the booster immunization, showing a vaccine efficacy of 51.9% (95% CI, 31.3% to 66.4%). Most infections were detected 90 days after intervention during a period when XBB was prevalent in the community. Adverse reactions were reported by 64% of participants after the LVRNA012 vaccination, but most of them were mild or moderate. The booster vaccination with the LVRNA012 mRNA vaccine could significantly enhance neutralizing antibody titers against the Omicron variant XBB.1.5 (GMT 132.3 [99.8, 175.4]) than did those in the placebo group (GMT 12.5 [8.4, 18.7]) at day 14 for the previously immunized individuals.

**Conclusion:**

The LVRNA012 mRNA vaccine is immunogenic, and shows robust efficacy in preventing COVID-19 during the omicron-predominate period.

**Clinical trial registration:**

ClinicalTrials.gov, identifier NCT05745545.

## 1 Introduction

Inactivated COVID-19 vaccines are effective against SARS-CoV-2 and have good safety in the population, which have been widely utilized in numerous countries for large-scale vaccination programs (1). However, studies have demonstrated that immunity to two doses of the ancestral-strain inactivated vaccine declined rapidly over time (2–5), especially with the emergence of highly transmissible SARS-CoV-2 variants (such as Omicron) which escaped the neutralizing antibodies induced by the original SARS-CoV-2 strain vaccine (6–8). The protective effects induced by primary immunization have been greatly challenged and the booster doses after the primary immunization have become a concern globally.

The World Health Organization (WHO) recommended the use of homologous or heterologous boosters to restore and extend protection in individuals who have received the two doses of inactivated vaccine, and heterologous boosters were more effective (9, 10). The study from Brazil indicated that during the period of the Omicron, vaccine effectiveness against symptomatic disease 8-59 days after receiving a homologous booster and a heterologous (BNT162b2) booster was 8.6% (95% CI, 5.6-11.5) and 56.8% (95% CI, 56.3-57.3), respectively, and VE against severe COVID-19 was 73.6% (95% CI, 63.9-80.7) and 86.0% (95% CI, 84.5-87.4), respectively, in people who completed two doses of inactivated vaccine (11). Another study in Chile showed that for those who had completed a primary immunization with CoronaVac, the VE against symptomatic COVID-19 and related hospitalization was 78.8% (95% CI 76.8-80.6) and 86.3% (83.7-88.5) for a homologous booster, 96.5% (96.2-96.7) and 96.1% (95.3-96.9) for a BNT162b2 booster (12). When COVID-19 outbreaks first occurred, inactivated vaccines, mRNA vaccines, etc., played an important role in large geographic areas. However, in the subsequent booster phase, mRNA vaccines have the advantages of rapid development and update, and highly efficient immune responses compared to traditional vaccines, which puts mRNA technology at the forefront of the COVID-19 vaccine race (13–15).

Although severe respiratory disease declined during the COVID-19 pandemic and vaccines are available, vaccines need to be updated to cope with antigenically distinct variants. LVRNA012 is an mRNA vaccine encoding the full-length spike (S) protein of the SARS-CoV-2 variant (Omicron BA.5), including key mutation sites of the S proteins of BQ.1, XBB 1.5, and XBB.1.16. It is shown that LVRNA012 is not only highly immunogenic and safe, but also can induce high-level broad-spectrum cross-neutralizing activities against Omicron BA.5, XBB.1, and BQ1.1 in an unpublished pre-clinical animal study. Before the phase III trial, the monovalent vaccine LVRNA009 (wild-type strain), the monovalent vaccine LVRNA012 (BA.5 variant), and the bivalent vaccine LVRNA021 (Delta+BA.5), from the same mRNA vaccine platform, conducted Phase 1 and Phase 2 clinical trials. The three SARS-CoV-2 mRNA vaccines (LVRNA009 (16), LVRNA012, and LVRNA021) demonstrated good safety and tolerability in phase I trials. In the subsequent phase II trial results to be published, the mRNA vaccines were able to induce the production of neutralizing antibodies against SARS-CoV-2 in healthy individuals with a clinically acceptable safety profile. Considering that the majority of the Chinese population only received 2 or 3 doses of inactivated COVID-19 vaccine based on the ancestral strain and the potential benefits of the heterologous booster against Omicron variants, it is of great significance to carry out a clinical study of heterologous booster using Omicron BA.5 mRNA vaccine in a population that has already completed 2 or 3 doses of inactivated vaccines.

Here, we aimed to report the efficacy, safety, and immunogenicity results of the mRNA vaccine LVRNA012 as a booster in adults aged over 18 years who had previously received two or three doses of inactivated vaccine.

## 2 Methods

### 2.1 Study design and participants

This is a single-center, double-blind, randomized, placebo-controlled phase 3 study conducted at the first affiliated hospital of Bengbu Medical College in Bengbu, Anhui province, China. Healthy adults or adults with mild underlying diseases (including hypertension, mild diabetes mellitus, mild hypothyroidism, etc) at 18 years of age or older who have been inoculated with two or three doses of inactivated COVID-19 vaccines for at least six months were recruited, with a negative test for SARS-COV-2 infection by nucleic acid with the method of Reverse Transcription-Polymerase Chain Reaction (RT-PCR) within 48 hours. The main exclusion criteria were pregnancy or lactation, a history of COVID-19 infection within 6 months or human coronavirus infection or disease such as Severe Acute Respiratory Syndrome and Middle East Respiratory Syndrome before, or previous history of severe anaphylaxis to vaccines or drugs, or receipt of any other COVID-19 vaccine apart from inactivated COVID-19 vaccines, or receipt of any immunosuppressants or other immunomodulatory drugs for over 2 weeks within the past 6 months, or any disease that could seriously affect the function of the immune system.

The study was funded by AIM Vaccine Co., Ltd. The protocol and informed consent form (ICF) were approved by the Clinical Trial Ethics Committee of the first affiliated hospital of Bengbu Medical College. Written ICF was obtained from all potential participants before enrolment. This trial was conducted following national regulations and the principles of the Declaration of Helsinki and Good Clinical Practice, with registration at www.clinicaltrials.gov (NCT05745545). The study protocol, including the CONSORT checklist, can be found in S1 Study Protocol and S1 CONSORT Checklist.

### 2.2 Randomization and masking

The eligible participants were randomly assigned at a ratio of 1:1 to receive one dose of either LVRNA012 or placebo via intramuscular injection in the deltoid muscle, using block randomization with a block size of four. Randomization was performed by an independent statistician using SAS software (version 9.4), who played no further role in the study. A randomization code was assigned to each participant in the order of his/her enrolment, and the investigational products corresponding to the code were injected into each specific participant. The staff who prepared the vaccines and administrated vaccinations were aware of the treatment allocations of the participants, but they were not allowed to share the information with others and did not take part in any other process of the study. Other investigators, participants, and staff undertaking laboratory detection were masked during treatment administration. To keep the participants blind, a screen was set up between them and the vaccination administrators. When the participants received the injection, they were allowed to expose their upper arms through the screen, thus they could not see the syringe and didn’t know which vaccine they had accepted.

### 2.3 Procedures

LVRNA012 was a vaccine candidate manufactured by Liverna Therapeutics Inc, a subsidiary company of AIM Vaccine Co., Ltd. Its mRNA was produced by *in vitro* transcription, and further encapsulated into lipid nanoparticle (LNP). The product was manufactured in a liquid form at a concentration of 1.0 ml/piece containing 100ug mRNA which encodes SARS-COV-2 S-protein.

Vaccines were injected for one dose at 1.0 ml by unmasked vaccine administrators and the placebo was administrated at 0.5 ml one dose. These vaccine administrators couldn’t take part in any other aspect of the study. Participants were monitored for 30 min after the injection. Solicited adverse events (AEs) were recorded by diary card within 14 days and all other unsolicited events were recorded similarly during the 28-day follow-up for each participant. All serious adverse events (SAEs), adverse events of special interest (AESIs), and pregnancy-related events (including pregnancy outcomes, delivery characteristics, the condition of the newborn, and the growth and development within 1 month after birth) were collected within 6 months after the vaccination. All adverse events were graded according to the China NMPA guidelines and the correlation with vaccination by the investigator (17).

The first 100 participants enrolled were allocated to the immunogenicity subgroups. For these participants, blood samples before the vaccination and on days 7, 14, 28, 90, and 180 were collected for the detection of neutralizing antibodies against the current main epidemic strains, and extra venous blood samples were collected to detect cytokines of IL-2, IL-4, IL-13, IFN-γ (ELISpot) before the vaccination and on days 7, 14, 28, 90 after vaccination.

All participants were monitored for symptomatic COVID-19 infection after 7 days from the booster vaccination through remote visits or on-site visits. We captured suspected symptoms related to COVID-19 via remote visits, conducted about 8 times every month from the 7^th^ to the 90^th^ day after the booster and at least once a month from 90^th^ to 180^th^ day. On-site visits were conducted to collect the participants’ throat swab samples (for SARS-CoV-2 nucleic acid or antigen detection) when they have any possible symptoms or physical signs due to COVID-19, any respiratory-related symptoms, or pneumonia. The participants with suspected symptoms and positive nucleic acid or antigen tests were confirmed as COVID-19 cases and their backup specimens were finally sent to the central laboratory for SARS-CoV-2 strain type detection.

### 2.4 Outcomes

The primary endpoints for vaccine efficacy (VE) were the person-year incidence rate of symptomatic COVID-19 cases of any severity confirmed by laboratory testing (SARS-CoV-2 nucleic acid or antigen detection) occurring from 14 days to 180 days after booster vaccination of LVRNA012 or placebo, and the secondary efficacy endpoints were the person-year incidence of severe and critical COVID-19 cases or deaths due to COVID-19. The exploratory efficacy endpoints were also set to evaluate the protective efficacy of LVRNA012 against symptomatic COVID-19 cases of any severity occurring 7 days after immunization. The efficacy analysis was based on the intention-to-treat population who did not violate the protocol and with no exposure to COVID-19 from the 0^th^ day to the 7^th^ day.

The secondary endpoints for safety evaluation were solicited AEs within 30 minutes and on days 14, and 28 after vaccination, including local and systemic reactions, unsolicited AEs within 28 days, and SAEs, AESIs, and pregnancy events within 6 months. The safety endpoints were based on all participants enrolled and received one shot.

The secondary endpoints for immunogenicity were humoral immune responses including neutralizing antibodies induced by LVRNA012 in the intention-to-treat cohort of immunogenicity subgroups. The titers of neutralizing antibodies to live SARS-CoV-2 virus of current strains were detected with cytopathic effect based microneutralization assay at baseline and 7, 14, 28, 90, and 180 days after vaccination. Exploratory immunogenic endpoints were T-cell responses, measured by IL-2, IL-4, IL-13, and IFN-γ at days 0, 7, 14, 28, and 90. The cytokines were measured by ELISpot, and the results were expressed as the number of spot-forming cells per 1,000,000 peripheral blood mononuclear cells (PBMCs).

### 2.5 Statistical analysis

The sample size was determined based on statistical power calculations and driven by the primary endpoint. Assume that approximately 9% of the incidence rate of COVID-19 in the study area and the expected VE of the vaccine is no less than 60%, a sample size of 3200 could achieve a 90% power of the test when the superiority margin of VE is 30%, the allocation ratio of the experimental group to the placebo control group is 1:1, the test level is 0.025 on one side. During an observation period of 6 months, we estimated that about 162 eligible cases of any RT-PCR-confirmed COVID-19 with clinical symptoms could be determined.

All statistical analysis was performed using statistical software SAS 9.4 and GraphPad Prism 8.0. Categorical data, including basic participant characteristics and incidence of adverse events, were expressed as counts and percentages. The difference between groups was calculated with χ² test or Fisher’s exact test. Measurement data was expressed statistically with mean or geometric mean, median, standard deviation, maximum value, and minimum value. Geometric mean titers (GMTs) of neutralizing antibodies were calculated with the two-sided 95% confidence interval (CI) based on the t-distribution of the log-transformed titers, and the statistical significance was tested by *t*-test. Cellular immunity (IFN-γ, IL-2, IL-4, IL-13, and the ratio of Th1 and Th2 expressing level) was statistically described, and the differences between groups were calculated with nonparametric tests. Vaccine efficacy and 95% CI were calculated using the Cox regression model based on the annual incidence rate of COVID-19 cases confirmed by the central laboratory with clinical symptoms since the 14^th^ day after the booster. The statistical difference was analyzed by the exact Poisson regression model and the significance level was set at p < 0.05 (two-sided).

## 3 Results

### 3.1 Participants

**Figure 1:**
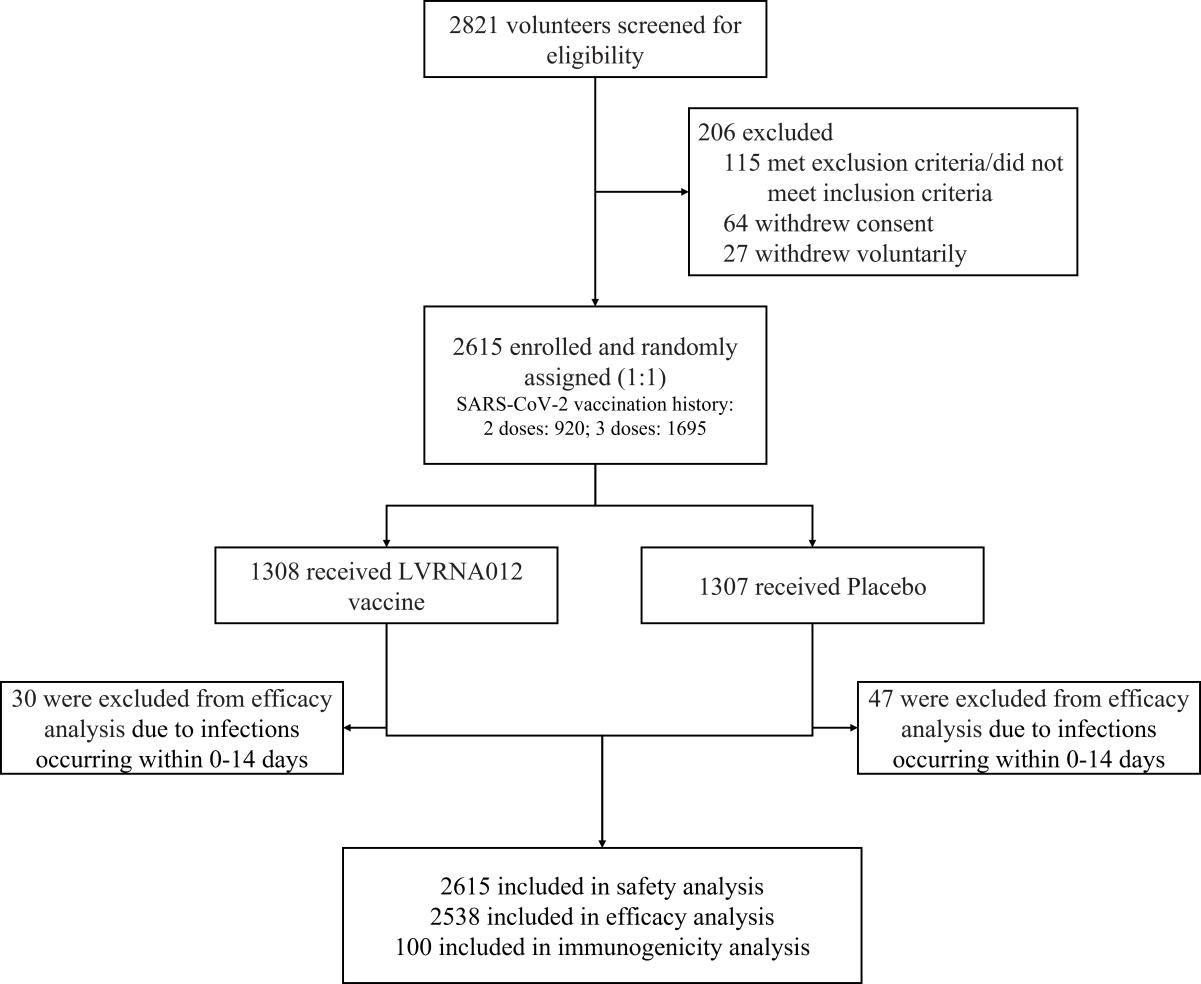
Trial profile. One subject was assigned to the vaccine group but was actually vaccinated with placebo. 77 subjects with nucleic acid or antigen-confirmed COVID-19 infections occurring within 0-14 days were excluded from the efficacy analyses.

Between January 4 and January 15, 2023, we recruited 2,812 subjects, including both male and non-pregnant females, aged 18 years or older who have completed 2 or 3 doses of inactivated COVID-19 vaccine more than 6 months ago. Individuals with a previous COVID-19 history or confirmed SARS-CoV-2 infection and pregnant women were excluded from this study. A total of 2615 participants were equally assigned in a 1:1 ratio to either the vaccine group (1308) or the placebo group (1307), with the first 50 subjects enrolled in each group (100 in total) being the immunization subcohort for immunogenicity analysis (**Figure 1**, **Supplementary Table S1**). 27 participants withdrew before receiving their booster and one subject was assigned to the vaccine group but was vaccinated with the placebo. The primary analysis was performed based on the intervention-modified intention-to-treat cohort. During the trial period, participants underwent the first wave of COVID-19 outbreaks from January to April 2023 with BA.5 as the predominant mutant strain, and the second wave of COVID-19 outbreaks from May to July with XBB as the predominant mutant strain.

In the total cohort, the median age of the participants was 33.0 (IQR: 27.0, 41.0) years, 84.3% identified as male, and the ethnic distribution included 97.0% of Han Chinese. The demographic characteristics of the participants were comparable between the LVRNA012 vaccine group and the placebo group (**Table 1**). 64.8% of the participants had received three doses of inactivated vaccine. No difference was noted in terms of the intervals between the last dose of inactivated COVID-19 vaccines and the booster dose across the groups (12.4 months [IQR:10.4, 16.1]) in the vaccine group and 12.3 months [IQR: 10.2, 16.1] in the placebo group).

**Table 1:** Baseline characteristics of the participants.

	All randomized participants		Immunogenicity subset	
	LVRNA012 group (n=1308)	Placebo group (n=1307)	LVRNA012 group (n=49)	Placebo group (n=51)
Sex				
Female	205 (15.7)	209 (16.0)	10 (20.4)	11 (21.6)
Male	1103 (84.3)	1098 (84.0)	39 (79.6)	40 (78.4)
Age, years				
Median age (IQR)	33.0 (27.0, 41.0)	32.0 (27.0, 41.0)	34.0 (29.0, 39.0)	37.0 (32.0, 40.0)
**Height (cm)**	170.4 (7.6)	170.1 (7.6)	168.6 (9.5)	170.0 (7.7)
**Weight (kg)**	72.2 (13.2)	71.9 (13.7)	66.9 (9.5)	68.7 (11.0)
Ethnicity				
Han	1269 (97.0)	1265 (96.8)	48 (98.0)	49 (96.1)
Others	39 (3.0)	42 (3.2)	1 (2.0)	2 (3.9)
SARS-CoV-2 vaccination history (%)				
2 doses	458 (35.0)	462 (35.3)	19 (38.8)	23 (45.1)
3 doses	850 (65.0)	845 (64.7)	30 (61.2)	28 (54.9)
Time interval since the last priming dose of inactivated vaccine, months	12.4 (10.4, 16.1)	12.3 (10.2, 16.1)	12.6 (9.5, 16.4)	12.7 (10.8, 16.7)

Data are n (%), mean (SD), or median (IQR). n, Number of participants; SD, Standard deviation; IQR, Interquartile range.

### 3.2 Efficacy

**Figure 2:**
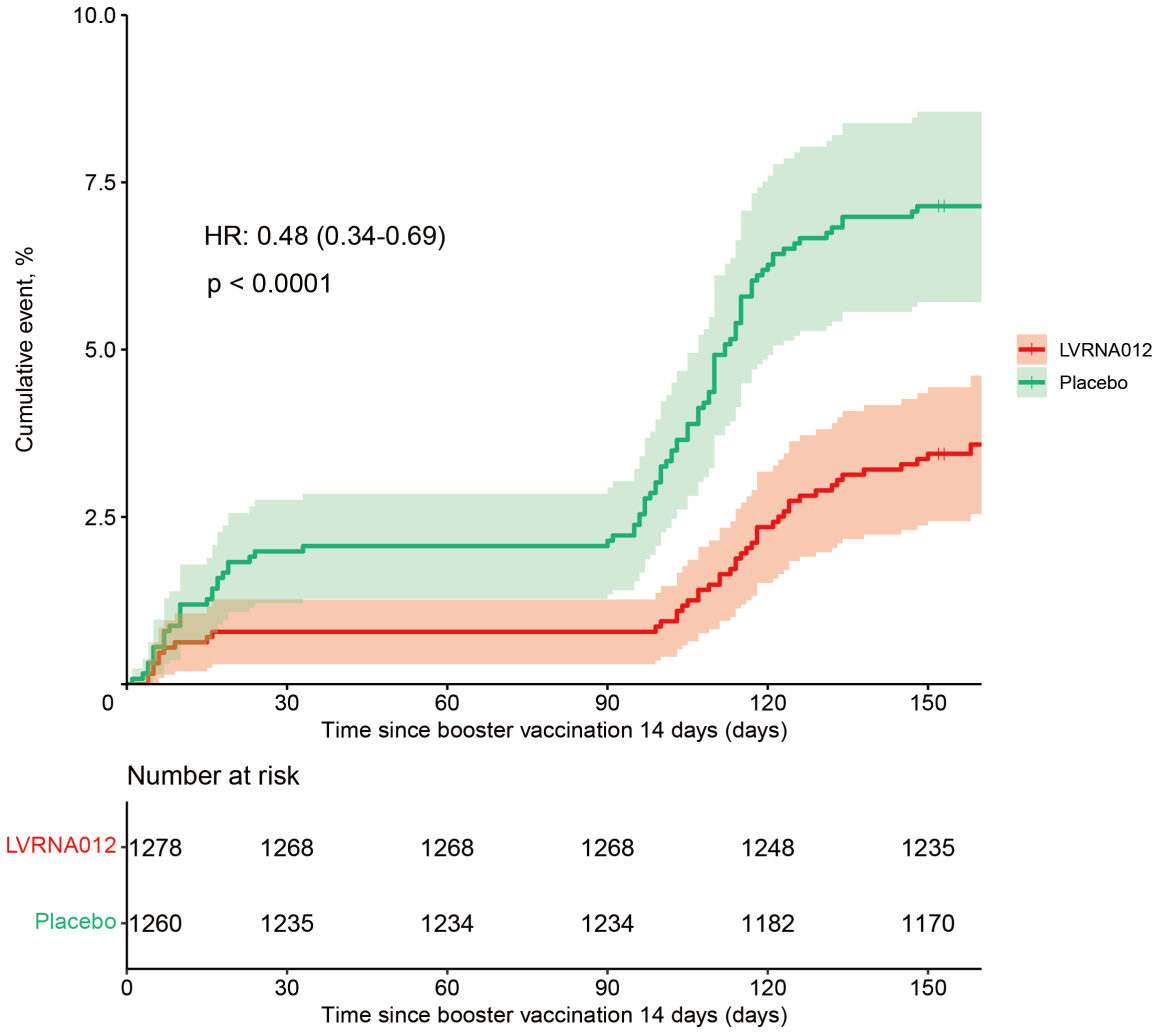
Cumulative Incidence of COVID-19 Incident Cases (1 – Kaplan-Meier Estimate) 14 days following the vaccination of the LVRNA012 vaccine or the placebo. Shown is the cumulative incidence curve of the first COVID-19 occurrence after the vaccination of the LVRNA012 vaccine or the placebo, as calculated employing the Kaplan–Meier method. The shading represents 95% confidence intervals. Each symbol represents the onset of a COVID-19 case.

As of the data cut-off date (15 July 2023), a total of 212 infections had occurred in 2,615 participants aged 18 years or older who were available for assessment and had no evidence of previous SARS-CoV-2 infection. Of these, 30 vaccinees and 47 placebo recipients developed symptomatic COVID-19 infection within 14 days of a booster vaccination, which were excluded from the vaccine efficacy analysis. In addition, one vaccine and five placebo recipients developed infection between 7 and 14 days after vaccination. After 14 to 180 days of booster vaccination, 45 vaccinated and 90 placebo recipients developed symptomatic COVID-19 infections, representing a vaccine efficacy rate of 51.9% (95% CI, 31.3 to 66.4) (**Figure 2**, **Supplementary Figure S1**). There was one case of repeat infection in each of the vaccine subjects and placebo subjects, with the first infection occurring within 4 days of vaccination and the second occurring about 5 months after vaccination, although the symptoms of infection were mild. Notably, the vaccine’s protection against the COVID-19 virus is higher within 120 days than within 180 days (69.4 [46.4, 82.6] *vs* 51.9 [31.3, 66.4]) (**Table 2**). The protective efficacy of the vaccine obtained after the inclusion of cases of infections presenting at 7-14 days was 53.4 (95% CI, 33.7, 67.2).

**Table 2:** Protection against COVID-19 diseases of the LVRNA012 vaccine.

	LVRNA012 group	Placebo group	Hazard ratio (%)	Protection (95% CI)	P-value
Protection since 14 days after vaccination					
**Number of cases, n**	1278	1260			
14-30 days	10 (0.8%)	19 (1.5%)	51.7 (24.1, 111.3)	48.3 (-11.3, 75.9)	0.0917
14-90 days	10 (0.8%)	25 (1.9%)	39.3 (18.9, 81.8)	60.7 (18.2, 81.1)	0.0125
14-120 days	16 (1.3%)	51 (4.0%)	30.6 (17.4, 53.6)	69.4 (46.4, 82.6)	0.0197
14-180 days	45 (3.5%)	90 (7.1%)	48.1 (33.6, 68.7)	51.9 (31.3, 66.4)	<0.0001
Protection since 7 days after vaccination					
**Number of cases, n**	1279	1265			
7-30 days	11 (0.9%)	24 (1.9%)	45.1 (22.1, 92.1)	54.9 (7.9, 77.9)	0.0288
7-90 days	11 (0.9%)	30 (2.4%)	36.0 (18.1, 71.9)	64.0 (28.1, 81.9)	0.0038
7-120 days	17 (1.3%)	56 (4.4%)	29.6 (17.2, 50.9)	70.4 (49.1, 82.8)	<0.0001
7-180 days	46 (3.6%)	95 (7.5%)	46.6 (32.8, 66.3)	53.4 (33.7, 67.2)	<0.0001

Data are n (%) or n/N (%). The hazard ratio is estimated by Cox regression analysis. Protection was calculated as a one minus hazard ratio.

### 3.3 Safety

2437 adverse reactions were reported by 1033 (39.46%) of 2 615 participants within 28 days after receiving the booster vaccination with the LVRNA012 vaccine or the placebo (**Table 3**, **Supplementary Tables S2-4**). Solicited adverse reactions at the injection site were reported more frequently in the vaccine group than the placebo group after the booster vaccination (582 [44.50%] *vs* 56 [4.28%], p-value<0.0001). Local pain at the injection site was the most common adverse reaction, reported in 42.97% of the participants in the vaccine group and 3.98% in the placebo group (p-value<0.0001). Systemic solicited adverse reactions were more common in the vaccine group than in the placebo group after booster vaccination (644 [49.24%] *vs* 158 [12.09%], p-value<0.0001). Fever was the most common systemic AE in both the vaccine group (588 [44.95%]) and the placebo group (110 [8.42%], p-value<0.0001). Unsolicited adverse reactions after the injection were similar among participants in the vaccine and placebo groups (22 [1.68%] *vs*. 26 [1.99%]). Of these, cough was the most common unsolicited adverse reaction in both the vaccine and placebo groups (5 [0.38%] *vs* 7 [0.54%]).

**Table 3:** Solicited and unsolicited adverse reactions occurred within 28 days after the vaccination.

		Vaccine group (N=1308)	Placebo group (N=1307)	Total (N=2615)	P-value^*^
Adverse reactions					
Total	Any	833 (63.69%)	199 (15.23%)	1033 (39.46%)	<0.0001
	≥Grade 3	204 (15.60%)	10 (0.77%)	214 (8.18%)	<0.0001
Solicited adverse reactions					
	Any	828 (63.30%)	185 (14.15%)	1013 (38.74%)	<0.0001
	≥Grade 3	204 (15.60%)	10 (0.77%)	214 (8.18%)	<0.0001
Administration-site adverse reactions					
Total	Any	582 (44.50%)	56 (4.28%)	638 (24.40%)	<0.0001
	≥Grade 3	14 (1.07%)	0	14 (0.54%)	0.0001
Pain	Any	562 (42.97%)	52 (3.98%)	614 (23.48%)	<0.0001
	≥Grade 3	8 (0.61%)	0	8 (0.31)	0.0077
Induration	Any	80 (6.12%)	0	80 (3.06%)	<0.0001
	≥Grade 3	1 (0.08%)	0	1 (0.04%)	1.0000
Redness	Any	47 (3.59%)	2 (0.15%)	49 (1.87%)	<0.0001
	≥Grade 3	1 (0.08%)	0	1 (0.04%)	1.0000
Swelling	Any	112 (8.56%)	3 (0.23%)	115 (4.40%)	<0.0001
	≥Grade 3	6 (0.46%)	0	6 (0.23%)	0.0311
Skin eruption	Any	3 (0.23%)	2 (0.15%)	5 (0.19%)	1.0000
Itch	Any	71 (5.43%)	8 (0.61%)	79 (3.02%)	<0.0001
	≥Grade 3	1 (0.08%)	0	1 (0.04%)	1.0000
Cellulitis	Any	1 (0.08%)	0	1 (0.04%)	1.0000
Systemic adverse reactions					
Total	Any	644 (49.24%)	158 (12.09%)	802 (30.67%)	<0.0001
	≥Grade 3	196 (14.98%)	8 (0.61%)	204 (7.80%)	<0.0001
Fever	Any	588 (44.95%)	110 (8.42%)	798 (26.69%)	<0.0001
	≥Grade 3	192 (14.68%)	8 (0.61%)	200 (7.65%)	<0.0001
Diarrhea	Any	37 (2.83%)	21 (1.61%)	58 (2.22%)	0.0455
	≥Grade 3	1 (0.08%)	0	1 (0.04%)	1.0000
Nausea	Any	29 (2.22%)	8 (0.61%)	37 (1.41%)	0.0007
Vomiting	Any	11 (0.84%)	5 (0.38%)	16 (0.61%)	0.2087
Headache	Any	138 (10.55%)	20 (1.53%)	158 (6.04%)	<0.0001
	≥Grade 3	1 (0.08%)	0	1 (0.04%)	1.0000
Myalgia (Non-inoculated sites)	Any	48 (3.67%)	13 (0.99%)	61 (2.33%)	<0.0001
	≥Grade 3	4 (0.31%)	0	4 (0.15%)	0.1247
Joint pain	Any	42 (3.21%)	12 (0.92%)	54 (2.07%)	<0.0001
	≥Grade 3	2 (0.15%)	0	2 (0.08%)	0.4998
Shiver	Any	74 (5.66%)	3 (0.23%)	77 (2.94%)	<0.0001
Loss of appetite	Any	51 (3.90%)	8 (0.61%)	59 (2.26%)	<0.0001
Fatigue	Any	124 (9.48%)	23 (1.76%)	147 (5.62%)	<0.0001
	≥Grade 3	6 (0.46%)	0	6 (0.23%)	0.0311
Acute allergic reactions	Any	4 (0.31%)	0	4 (0.15%)	0.1247
Unsolicited adverse reactions					
Total	Any	22 (1.68%)	26(1.99%)	48 (1.84%)	0.5645
	≥Grade 3	0	2 (0.15%)	2 (0.08%)	0.6464

Data are n (%). n = number of participants. % = proportion of participants. Any = all the participants with any grade adverse reactions or events. The analysis was based on the intervention-modified intention-to-treat cohort. *Calculated with χ² test or Fisher’s exact test.

Overall, 9 serious adverse events were reported (7 [0.54%] in the vaccine group and 2 [0.15%] in the placebo group), none of which were related to the vaccine, except for 1 case of myocardial infarction that was related to vaccination with placebo (**Supplementary Table S5**). One subject (0.08%) in the vaccine group exhibited hemoptysis unrelated to vaccination. Two subjects (0.15%) in the vaccine group suffered heart diseases (1 myocardial infarction and 1 angina), while 1 subject in the placebo group suffered a myocardial infarction. In addition, two cases of infectious and invasive diseases (1 infectious pneumonia with negative nucleic acid test and 1 viral myocarditis) appeared to be present in the vaccine group, whereas none were present in the placebo group.

### 3.4 Immunogenicity

**Figure 3:**
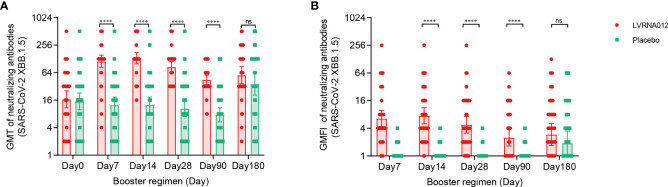
Neutralising antibodies against SARS-CoV-2 XBB.1.5 before and after a booster vaccination. GMTs of neutralizing antibodies to SARS-CoV-2 XBB.1.5 **(A)**. GMFI of neutralizing antibodies to wild-type SARS-CoV-2 XBB.1.5 **(B)**. Error bars indicate 95% CIs. The analysis was based on the intervention-modified intention-to-treat cohort. Measurements on day 0 were taken immediately before vaccination. P values result from a comparison between the two treatment groups using t-tests for log-transformed antibody titers. ****p<0·0001. ns represents not significant.

At baseline, serum NAbs against SARS-CoV-2 XBB.1.5 were measured in 100 participants (49 in the LVRNA012 vaccine group and 51 in the placebo group) and were similar in two groups: GMTs of 16.7 (95% CI 10.9, 25.6) in the LVRNA012 vaccine group and 15.0 (95% CI 9.8, 22.8) in the placebo group (**Figure 3**, **Supplementary Table S6**). At day 7, the LVRNA012 vaccine group had significantly higher serum NAbs GMTs against SARS-CoV-2 XBB.1.5 than the placebo group (113.2 [95% CI 83.1, 154.1] in the LVRNA012 vaccine group; 12.5 [95% CI 8.4, 18.6] in the placebo group; p<0.0001). The booster immunization with the LVRNA012 vaccine elicited 9.7-10.7 times higher serum NAbs responses than that elicited by the placebo between days 7 and 14. Neutralization titers remained relatively high 180 days after booster vaccination with the LVRNA012 vaccine (56.3 [95% CI 36.7, 86.6]) compared to placebo (36.1 [20.5, 63.6]).

**Figure 4:**
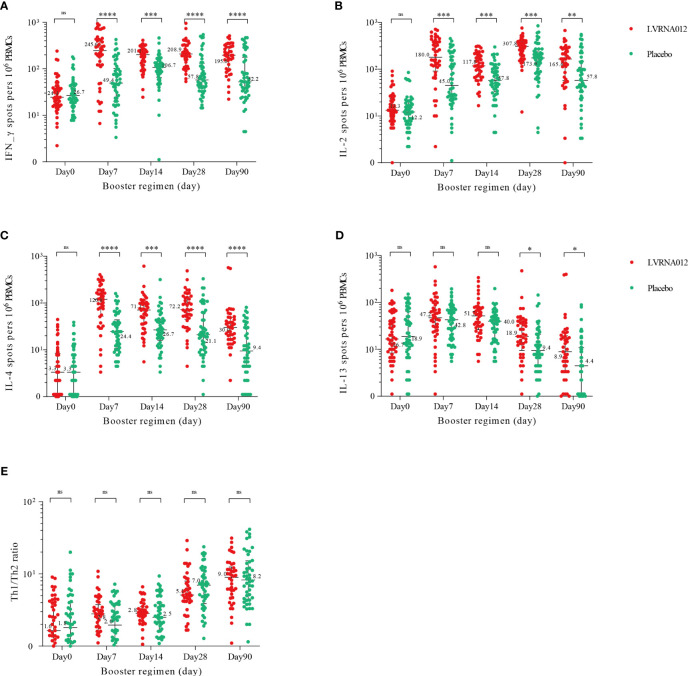
SARS-CoV-2 spike-specific T-cell cytokine responses before and after boosting. IFN-γ **(A)**, IL-2 **(B)**, IL-2 **(C)**, and IL-13 **(D)** cytokine concentrations. Th1/Th2 ratios **(E)** were calculated by summing IFN-γ and IL-2 cytokine levels and then dividing by the sum of IL-4 and IL-13 cytokine levels. IFN-γ=interferon-γ. IL-2, interleukin-2. IL-4, interleukin-4. IL-13, interleukin-13. PBMCs, peripheral blood mononuclear cells. *p<0·05. **p<0·01. ***p<0·001. ****p<0·0001. ns represents not significant.

The SARS-CoV-2 spike protein-specific IFN-γ and IL-2 ELISpot responses were significantly increased 7 days after booster immunization with the LVRNA012 vaccine (p<0.001) and the responses in the LVRNA012 group were 10-12 times higher than those in the placebo group, remaining high until 90 days (**Figure 4**, **Supplementary Table S7**). The booster dose also enhanced specific IL-4 responses in the LVRNA012 group, with a more dramatic increase (35.7 times) compared to the placebo group at day 7 after the booster dose. Although the number of IL-13-secreting T cells was relatively high at baseline before boost, it increased only slightly (2.3-2.9-fold) after 7 days of boost, and there was no significant difference between the LVRNA012 vaccine group and placebo group (p-value>0.05).

## 4 Discussion

The trial provides evidence of short-term efficacy, good safety, and high immunogenicity of the SARS-CoV-2 Omicron BA.5 mRNA vaccine (LVRNA012) given as the booster in subjects aged 18 years or older who have completed two or three doses of inactivated COVID-19 vaccine more than 6 months ago. The LVRNA012 vaccine has 53.4% (95% CI, 33.7% to 67.2%) efficacy in preventing symptomatic SARS-CoV-2 infections 7 days after vaccination, while protection against more than moderate symptoms could not be definitively assessed because of the small number of cases. Although the protective efficacy of the vaccine against COVID-19 attenuates as the strain mutates and the neutralizing antibody titer decreases (18), it is encouraging to note that we found that the LVRNA012 vaccine maintained a high protective efficacy (63.6 [30.6, 80.9]) up to three months, in which the predominant strain prevalent in China was BA.5.

A total of 63.69% of participants reported adverse reactions after LVRNA012 vaccination, most of which were mild or moderate, and similar in incidence and severity to other omicron-containing mRNA-based COVID-19 vaccines (19, 20). As with the mRNA-1273.214 booster, pain in the vaccination site was the most common adverse reaction (79.4% of mRNA-1273.214 *vs*. 42.97% of LVRNA012). We found that booster vaccination with the LVRNA012 mRNA vaccine was able to significantly enhance neutralizing antibody titers against the Omicron variant XBB.1.5 within a short period (7 days) and remained high for 28 days, followed by a slow decrease and that the corresponding neutralizing antibodies were still detectable after six months. The LVRNA012 mRNA vaccine boosters had a significantly stronger specific T cell response, characterized by a CD4^+^ T response expressing Th1 cytokines (IFN-γ, IL-2) and Th2 cytokines (IL-4), which may be important for persistence against the emerging SARS-CoV-2 variant. Convergent patterns of change in neutralizing antibodies and cytokines induced by the vaccine imply a potential synergistic role of cellular and humoral immunity in the fight against infection. Interestingly, we also observed a 2.4-.3.0-fold increase in IL-13 concentrations within 14 days of either vaccine or placebo vaccination, and the reasons for this phenomenon will have to be investigated in follow-up.

In the face of the continuous emergence of severe acute respiratory syndrome coronavirus 2 (SARS-CoV-2) variants and the weakening of vaccine-induced neutralizing antibodies as an indicator of protection, booster vaccination with a vaccine developed based on the variants is necessary (20, 21). The LVRNA012 mRNA vaccine induces the production of neutralizing antibodies and cytokines in large quantities within a short period, thereby activating the immune system to fight infection, which is important for blocking the mass population transmission of the SARS-CoV-2 variant that may occur later. During the first three months of this study, the prevalent COVID-19 variant in China was predominantly omicron BA.5, and the LVRNA012 mRNA vaccine developed based on BA.5 provided high protection against this prevalent strain (18). The LVRNA012 mRNA vaccine also showed some protective efficacy against the XBB variant, which subsequently emerged and dominated the population.

There are several limitations to this study. First, although we planned to recruit adults aged 18 years and older into this study, we recruited predominantly young male participants, which does not effectively reflect the population at greatest risk of serious outcomes. Second, the assumption on the VE for the LVRNA012 mRNA vaccine was at least 60%, but the sample size of this study also observed a difference in the risk of morbidity between the vaccine and placebo groups. Besides, hospitalization and death did not occur among infected individuals, making it difficult to assess the effectiveness of the vaccine against severe outcomes. Third, although we confirmed symptomatic COVID-19 infections with nucleic acid or antigen testing, some individuals with mild symptoms after exposure or infection with the Omicron variant may have been missed, and thus the assessment of vaccine efficacy and neutralizing antibody levels would have been somewhat confounded. In addition, at the later stage of recruiting subjects, the widespread spread of COVID-19 in the population was such that we were unable to reach our target enrolment, which had a certain impact on the statistical power and validity of the study. Furthermore, considering participant compliance, we relaxed the frequency of monitoring after 90 days of vaccination, which may result in underreporting of infections. Finally, the reason for the sudden increase in XBB.1.5-neutralizing antibody titers after six months is not well understood and may be related to an increase in the number of asymptomatic infections in the population or to the fact that no large-scale population-based blood collection for neutralizing antibody testing has been carried out, which needs to be investigated in further experiments.

## 5 Conclusions

The heterologous booster regimen using the LVRNA012 mRNA vaccine was safe and had high immunogenicity and protective efficacy. The substantial increase in antibody titers and humoral immunity after heterologous boosting was encouraging and maintained a high intensity over six months, although immune persistence over a longer time needs to be further investigated. Our findings support booster vaccination with the LVRNA012 mRNA vaccine, fueling its dissemination in large populations.

## Data availability statement

The original contributions presented in the study are included in the article/**Supplementary Material**. Further inquiries can be directed to the corresponding authors.

## Ethics statement

The studies involving humans were approved by Clinical Trial Ethics Committee of the first affiliated hospital of Bengbu Medical College. The studies were conducted in accordance with the local legislation and institutional requirements. The participants provided their written informed consent to participate in this study.

## Author contributions

HZho: Writing – review & editing, Resources, Methodology, Investigation. HZhe: Writing – original draft, Software, Methodology, Formal analysis. YP: Writing – review & editing, Visualization, Data curation. YS: Writing – review & editing, Methodology, Investigation, Data curation. XY: Writing – review & editing, Data curation. WW: Writing – review & editing, Validation, Data curation. SL: Writing – review & editing, Methodology, Formal analysis, Data curation. YD: Writing – review & editing, Supervision, Resources, Formal analysis, Data curation. SJ: Writing – review & editing, Resources, Data curation. YW: Writing – review & editing, Methodology, Data curation. XZ: Writing – review & editing, Supervision, Investigation. LPL: Writing – review & editing, Supervision, Investigation. ZD: Writing – review & editing, Supervision, Investigation. LL: Writing – review & editing, Supervision, Investigation. FZ: Writing – review & editing, Supervision, Software. QW: Writing – review & editing, Visualization, Resources. JL: Writing – review & editing, Supervision, Funding acquisition. FCZ: Writing – review & editing, Visualization, Supervision, Resources, Conceptualization.
