# Supplementary material for: Efficacy, safety, and immunogenicity of SARS-CoV-2 mRNA vaccine (Omicron BA.5) LVRNA012: a randomized, double-blind, placebo-controlled phase 3 trial
Source: Front Immunol. 2024 Jun 6;15:1407826. doi: 10.3389/fimmu.2024.1407826 (PMC11187303; doi:10.3389/fimmu.2024.1407826)
Supplement: Supplementary file 1 [file DataSheet_1.docx]

**Supplemental material**

**Content**

[Table S1. Reasons for 115 volunteers not meeting screening criteria. 2](#_Toc166080955)

[Table S2. Unsolicited adverse reactions occurred within 28 days after the vaccination. 3](#_Toc166080956)

[Table S3. Solicited and unsolicited adverse events occurred within 28 days after the vaccination. 5](#_Toc166080957)

[Table S4. Unsolicited adverse events occurred within 28 days after the vaccination. 7](#_Toc166080958)

[Table S5. Serious adverse events occurred within 6 months after the booster vaccination 11](#_Toc166080959)

[Table S6. Neutralizing antibodies to SARS-CoV-2 XBB.1.5 before and after a heterogeneous boost vaccination. 12](#_Toc166080960)

[Table S7. SARS-CoV-2 spike-specific cytokine T cells responses before and after a heterogeneous boost vaccination 13](#_Toc166080961)

[Figure S1. Cumulative Incidence of COVID-19 Incident Cases (1 – Kaplan-Meier Estimate) 7 days following the vaccination of the LVRNA012 vaccine or the placebo. 15](#_Toc166080962)

[Figure S2. Cumulative Incidence of COVID-19 Incident Cases (1 – Kaplan-Meier Estimate) 7 days following the vaccination of the LVRNA012 vaccine or the placebo in participants who had previously received two or three doses of inactivated vaccine. 16](#_Toc166080963)

[Figure S3. Trends of mutant strains in native cases of COVID-19 infections in China. 17](#_Toc166080964)

[Figure S4. Trends in COVID-19 of influenza-like illnesses and influenza virus positivity rates in China. 18](#_Toc166080965)

# **Table S1. Reasons for 115 volunteers not meeting screening criteria.**

| **No.** | **Reasons for not eligible** | **Number of volunteers** |
| --- | --- | --- |
| 1 | Have received 2 or 3 doses of inactivated COVID-19 vaccine, but the last dose of inactivated COVID-19 vaccine was received less than 6 months ago. | 3 |
| 2 | Subjects who are not in a healthy state or have a serious underlying medical condition. | 1 |
| 3 | Women of childbearing potential (amenorrhoea of less than 1 year or no record of surgical sterilisation) or women known to be pregnant or breastfeeding who have not been using effective contraception for 14 days prior to the first dose of vaccine. | 6 |
| 4 | Individuals with abnormal and clinically significant vital signs | 56 |
| 5 | COVID-19 infection or use of any COVID-19 prophylaxis other than 3 doses of novel inactivated coronavirus vaccine within the last 6 months. | 17 |
| 6 | Axillary temperature ≥37.3°C detected on the day of vaccination or fever in the last 24 hours (axillary temperature ≥37.3°C or oral temperature ≥37.5°C). | 4 |
| 7 | Previous history of severe allergic or anaphylactic reactions to vaccines or drugs, e.g. urticaria, severe skin eczema, dyspnoea, laryngeal oedema, angioneurotic oedema. | 6 |
| 8 | Participated in a clinical study of another drug within 28 days prior to vaccination or planned to participate in a clinical study of another drug within 6 months after vaccination. | 1 |
| 9 | Confirmation of a condition that affects the functioning of the immune system, such as cancer, congenital or acquired immunodeficiency, or uncontrolled autoimmune disease, based on a known medical history or diagnosis. | 5 |
| 10 | Suspected or known alcohol dependence or drug abuse | 3 |
| 11 | Other factors that the researcher considered inappropriate for participation in this study. | 14 |
| Total | | 115 |

# **Table S2. Unsolicited adverse reactions occurred within 28 days after the vaccination.**

|  | | **Vaccine group (N=1308)** | **Placebo group (N=1307)** | **Total (N=2615)** | **P-value^*^** |
| --- | --- | --- | --- | --- | --- |
| **Unsolicited adverse reactions** | | | | | |
| Total | Any | 22 (1.68%) | 26(1.99%) | 48 (1.84%) | 0.5645 |
|  | ≥Grade 3 | 0 | 2 (0.15%) | 2 (0.08%) | 0.6464 |
| **Respirational, thoracic, and mediastinal disorders** | | | | | |
| Total | Any | 8 (0.61%) | 10 (0.77%) | 18 (0.69%) | 0.6464 |
| Cough | Any | 5 (0.38%) | 7 (0.54%) | 12 (0.46%) | 0.5801 |
| Runny nose | Any | 3 (0.23%) | 2 (0.15%) | 5 (0.19%) | 1.0000 |
| Dyspnea | Any | 1 (0.08%) | 0 | 1 (0.04%) | 1.0000 |
| Expectoration | Any | 1 (0.08%) | 1 (0.08%) | 2 (0.08%) | 1.0000 |
| Sore Throat | Any | 1 (0.08%) | 1 (0.08%) | 2 (0.08%) | 1.0000 |
| Stuffy nose | Any | 1 (0.08%) | 1 (0.08%) | 2 (0.08%) | 1.0000 |
| **Diseases of the gastrointestinal system** | | | | | |
| Total | Any | 4 (0.31%) | 5 (0.38%) | 9 (0.34%) | 0.7534 |
| Toothache | Any | 2 (0.15%) | 0 | 2 (0.08%) | 0.4998 |
| Canker sores | Any | 1 (0.08%) | 2 (0.15%) | 3 (0.11%) | 0.6247 |
| Gastroesophageal reflux | Any | 0 | 1 (0.08%) | 1 (0.04%) | 0.4998 |
| Bellyache | Any | 1 (0.08%) | 0 | 1 (0.04%) | 1.0000 |
| Dyspepsia | Any | 0 | 1 (0.08%) | 1 (0.04%) | 0.4998 |
| Abdominal distension | Any | 0 | 1 (0.08%) | 1 (0.04%) | 0.4998 |
| Non-infectious gingivitis | Any | 0 | 1 (0.08%) | 1 (0.04%) | 0.4998 |
| **Infectious and invasive diseases** | | | | | |
| Total | Any | 2 (0.15%) | 1 (0.08%) | 3 (0.08%) | 1.0000 |
| Infectious pneumonia | Any | 1 (0.08%) | 0 | 1 (0.04%) | 1.0000 |
| Bronchitis | Any | 1 (0.08%) | 0 | 1 (0.04%) | 1.0000 |
| Upper respiratory infections | Any | 0 | 1 (0.08%) | 1 (0.04%) | 0.4998 |
| **Systemic and administration-site adverse reactions** | | | | | |
| Total | Any | 5 (0.38%) | 4 (0.31%) | 9 (0.34%) | 1.0000 |
|  | ≥Grade 3 | 0 | 1 (0.08%) | 1 (0.04%) | 0.4998 |
| Fever | Any | 2 (0.15%) | 1 (0.08%) | 3 (0.11%) | 1.0000 |
|  | ≥Grade 3 | 0 | 1 (0.08%) | 1 (0.04%) | 0.4998 |
| Thoracalgia | Any | 0 | 1 (0.08%) | 1 (0.04%) | 0.4998 |
| Administration-site erythema | Any | 1 (0.08%) | 0 | 1 (0.04%) | 1.0000 |
| Administration-site Itch | Any | 1 (0.08%) | 0 | 1 (0.04%) | 1.0000 |
| Administration-site swelling | Any | 1 (0.08%) | 0 | 1 (0.04%) | 1.0000 |
| Chest discomfort | Any | 1 (0.08%) | 0 | 1 (0.04%) | 1.0000 |
| Armpit pain | Any | 1 (0.08%) | 0 | 1 (0.04%) | 1.0000 |
| Administration-site Discoloration | Any | 0 | 1 (0.08%) | 1 (0.04%) | 0.4998 |
| Fatigue | Any | 0 | 1 (0.08%) | 1 (0.04%) | 0.4998 |
| **Neurological disorders** | | | | | |
| Total | Any | 3 (0.23%) | 2 (0.15%) | 5 (0.19%) | 1.0000 |
| Dizziness | Any | 3 (0.31%) | 2 (0.23%) | 5 (0.27%) | 1.0000 |
| **Musculoskeletal and connective tissue diseases** | | | | | |
| Total | Any | 2 (0.15%) | 3 (0.23%) | 5 (0.19%) | 0.6871 |
| Joint pain | Any | 1 (0.08%) | 0 | 1 (0.04%) | 1.0000 |
| Limb pain | Any | 0 | 1 (0.08%) | 1 (0.04%) | 0.4998 |
| Back pain | Any | 1 (0.08%) | 0 | 1 (0.04%) | 1.0000 |
| Limb discomfort | Any | 0 | 1 (0.08%) | 1 (0.04%) | 0.4998 |
| Spinal pain | Any | 0 | 1 (0.08%) | 1 (0.04%) | 0.4998 |
| **Skin and subcutaneous tissue diseases** | | | | | |
| Total | Any | 1 (0.08%) | 3 (0.23%) | 4 (0.15%) | 0.3745 |
| Itch | Any | 0 | 1 (0.08%) | 1 (0.04%) | 0.4998 |
| Prurigo | Any | 1 (0.08%) | 0 | 1 (0.04%) | 1.0000 |
| Skin eruption | Any | 0 | 1 (0.08%) | 1 (0.04%) | 0.4998 |
| Urticaria | Any | 0 | 1 (0.08%) | 1 (0.04%) | 0.4998 |
| **Heart diseases** | | | | | |
| Total | Any | 1 (0.08%) | 1 (0.08%) | 2 (0.08%) | 1.0000 |
|  | ≥Grade 3 | 0 | 1 (0.08%) | 1 (0.04%) | 0.4998 |
| Ventricular extrasystoles | Any | 1 (0.08%) | 0 | 1 (0.08%) | 1.0000 |
| Myocardial infarction | Any | 0 | 1 (0.08%) | 1 (0.08%) | 0.4998 |
|  | ≥Grade 3 | 0 | 1 (0.08%) | 1 (0.04%) | 0.4998 |
| **Mental illness** | | | | | |
| Total | Any | 0 | 1 (0.08%) | 1 (0.04%) | 0.4998 |
| Insomnia | Any | 0 | 1 (0.08%) | 1 (0.04%) | 0.4998 |

Data are n (%). n = number of participants. % = proportion of participants. Any = all the participants with any grade adverse reactions or events. The analysis was based on the intervention-modified intention-to-treat cohort. *Calculated with χ² test or Fisher’s exact test.

# **Table S3. Solicited and unsolicited adverse events occurred within 28 days after the vaccination.**

|  | | **Vaccine group (N=1308)** | **Placebo group (N=1307)** | **Total (N=2615)** | **P-value^*^** | |
| --- | --- | --- | --- | --- | --- | --- |
| **Adverse events** | | |  |  |  | |
| Total | Any | 844 (64.53%) | 229 (17.52%) | 1073 (41.03%) | <0.0001 | |
|  | ≥Grade 3 | 210 (16.05%) | 14 (1.07%) | 224 (8.57%) | <0.0001 | |
| **Solicited adverse events** | | | | | |  |
| Total | Any | 830 (63.46%) | 190 (14.54%) | 1020 (39.01%) | <0.0001 | |
|  | ≥Grade 3 | 204 (15.60%) | 10 (0.77%) | 214 (8.18%) | <0.0001 | |
| **Administration-site adverse events** | | | | | |  |
| Total | Any | 582 (44.50%) | 56 (4.28%) | 638 (24.40%) | <0.0001 | |
|  | ≥Grade 3 | 14 (1.07%) | 0 | 14 (0.54%) | 0.0001 | |
| Pain | Any | 562 (42.97%) | 52 (3.98%) | 614 (23.48%) | <0.0001 | |
|  | ≥Grade 3 | 8 (0.61%) | 0 | 8 (0.31%) | 0.0077 | |
| Induration | Any | 80 (6.12%) | 0 | 80 (3.06%) | <0.0001 | |
|  | ≥Grade 3 | 1 (0.08%) | 0 | 1 (0.04%) | 1.0000 | |
| Redness | Any | 47 (3.59%) | 2 (0.15%) | 49 (1.87%) | <0.0001 | |
|  | ≥Grade 3 | 1 (0.08%) | 0 | 1 (0.04%) | 1.0000 | |
| Swelling | Any | 112 (8.56%) | 3 (0.23%) | 115 (4.40%) | <0.0001 | |
|  | ≥Grade 3 | 6 (0.46%) | 0 | 6 (0.23%) | 0.0311 | |
| Skin eruption | Any | 3 (0.23%) | 2 (0.15%) | 5 (0.19%) | 1.0000 | |
| Itch | Any | 71 (5.43%) | 8 (0.61%) | 79 (3.02%) | <0.0001 | |
|  | ≥Grade 3 | 1 (0.08%) | 0 | 1 (0.04%) | 1.0000 | |
| Cellulitis | Any | 1 (0.08%) | 0 | 1 (0.04%) | 1.0000 | |
| **Systemic adverse events** | | | | | |  |
| Total | Any | 647 (49.46%) | 164 (12.55%) | 811 (31.01%) | <0.0001 | |
|  | ≥Grade 3 | 196 (14.98) | 10 (0.77%) | 206 (7.88%) | <0.0001 | |
| Fever | Any | 588 (44.95%) | 112 (8.57%) | 700 (26.77%) | <0.0001 | |
|  | ≥Grade 3 | 192 (14.68%) | 10 (0.77%) | 202 (7.72%) | <0.0001 | |
| Diarrhea | Any | 38 (2.91%) | 22 (1.68%) | 60 (2.29%) | 0.0492 | |
|  | ≥Grade 3 | 1 (0.08%) | 0 | 1 (0.04%) | 1.0000 | |
| Nausea | Any | 29 (2.22%) | 8 (0.61%) | 37 (1.41%) | 0.0007 | |
| Vomiting | Any | 11 (0.84%) | 5 (0.38%) | 16 (0.61%) | 0.2087 | |
| Headache | Any | 139 (10.63%) | 22 (1.68%) | 161 (6.16%) | <0.0001 | |
|  | ≥Grade 3 | 1 (0.08%) | 0 | 1 (0.04%) | 1.0000 | |
| Myalgia | Any | 49 (3.75%) | 16 (1.22%) | 65 (2.49%) | <0.0001 | |
|  | ≥Grade 3 | 4 (0.31%) | 0 | 4 (0.15%) | 0.1247 | |
| Joint pain | Any | 42 (3.21%) | 12 (0.92%) | 54 (2.07%) | <0.0001 | |
|  | ≥Grade 3 | 2 (0.15%) | 0 | 2 (0.08%) | 0.4998 | |
| Rigor | Any | 74 (5.66%) | 3 (0.23%) | 77 (2.94%) | <0.0001 | |
| Loss of appetite | Any | 51 (3.90%) | 8 (0.61%) | 59 (2.26%) | <0.0001 | |
| Fatigue | Any | 124 (9.48%) | 23 (1.76%) | 147 (5.62%) | <0.0001 | |
|  | ≥Grade 3 | 6 (0.46%) | 0 | 6 (0.23%) | 0.0311 | |
| Acute allergic reactions | Any | 4 (0.31%) | 0 | 4 (0.15%) | 0.1247 | |
| **Unsolicited adverse events** | | | | | |  |
| Total | Any | 59 (4.51%) | 65 (4.97%) | 124 (4.74%) | 0.5825 | |
|  | ≥Grade 3 | 9 (0.69%) | 5 (0.38%) | 14 (0.53%) | 0.4277 | |

Data are n (%). n = number of participants. % = proportion of participants. Any = all the participants with any grade adverse reactions or events. The analysis was based on the intervention-modified intention-to-treat cohort. *Calculated with χ² test or Fisher’s exact test.

# **Table S4. Unsolicited adverse events occurred within 28 days after the vaccination.**

|  | | **Vaccine group (N=1308)** | **Placebo group (N=1307)** | **Total (N=2615)** | **P-value^*^** | |
| --- | --- | --- | --- | --- | --- | --- |
| **Unsolicited adverse events** | | | | | |  |
| Total | Any | 59 (4.51%) | 65 (4.97%) | 124 (4.74%) | 0.5825 | |
|  | ≥Grade 3 | 9 (0.69%) | 5 (0.38%) | 14 (0.53%) | 0.4277 | |
| **Respirational, thoracic, and mediastinal disorders** | | | | | |  |
| Total | Any | 18 (1.38%) | 13 (0.99%) | 31 (1.19%) | 0.4705 | |
|  | ≥Grade 3 | 1 (0.08%) | 0 | 1 (0.04%) | 1.0000 | |
| Cough | Any | 9 (0.69%) | 9 (0.69%) | 18 (0.69%) | 1.0000 | |
| Runny nose | Any | 5 (0.38%) | 2 (0.15%) | 7 (0.27%) | 0.4525 | |
| Throat pain | Any | 3 (0.23%) | 1 (0.08%) | 4 (0.15%) | 0.6247 | |
| Dyspnea | Any | 1 (0.08%) | 0 | 1 (0.04%) | 1.0000 | |
| Expectoration | Any | 1 (0.08%) | 1 (0.08%) | 2 (0.08%) | 1.0000 | |
| Throat irritation | Any | 1 (0.08%) | 0 | 1 (0.04%) | 1.0000 | |
| Sore Throat | Any | 1 (0.08%) | 1 (0.08%) | 2 (0.08%) | 1.0000 | |
| Stuffy nose | Any | 1 (0.08%) | 1 (0.08%) | 2 (0.08%) | 1.0000 | |
| Hemoptysis | Any | 1 (0.08%) | 0 | 1 (0.04%) | 1.0000 | |
|  | ≥Grade 3 | 1 (0.08%) | 0 | 1 (0.04%) | 1.0000 | |
| **Diseases of the gastrointestinal system** | | | | | |  |
| Total | Any | 13 (0.99%) | 10 (0.77%) | 23 (0.88%) | 0.6763 | |
| Toothache | Any | 6 (0.46%) | 2 (0.15%) | 8 (0.31%) | 0.2883 | |
| Diarrhoea | Any | 2 (0.15%) | 0 | 2 (0.08%) | 0.4998 | |
| Constipation | Any | 1 (0.08%) | 0 | 1 (0.04%) | 1.0000 | |
| Dry mouth | Any | 1 (0.08%) | 0 | 1 (0.04%) | 1.0000 | |
| Canker sores | Any | 1 (0.08%) | 3 (0.23%) | 4 (0.15%) | 0.3745 | |
| Chapped lips | Any | 1 (0.08%) | 0 | 1 (0.04%) | 1.0000 | |
| Gastroesophageal reflux | Any | 1 (0.08%) | 1 (0.08%) | 2 (0.08%) | 1.0000 | |
| Bellyache | Any | 1 (0.08%) | 1 (0.08%) | 2 (0.08%) | 1.0000 | |
| Dyspepsia | Any | 0 | 2 (0.15%) | 2 (0.08%) | 0.2497 | |
| Epigastric pain | Any | 0 | 1 (0.08%) | 1 (0.04%) | 0.4998 | |
| Abdominal distension | Any | 0 | 1 (0.08%) | 1 (0.04%) | 0.4998 | |
| Non-infectious gingivitis | Any | 0 | 1 (0.08%) | 1 (0.04%) | 0.4998 | |
| **Infectious and invasive diseases** | | | | | |  |
| Total | Any | 12 (0.92%) | 25 (1.91%) | 37 (1.41%) | 0.0323 | |
|  | ≥Grade 3 | 4 (0.30%) | 2 (0.15%) | 6 (0.23%) | 0.6871 | |
| COVID-19 | Any | 9 (0.69%) | 21 (1.61%) | 30 (1.15%) | 0.0285 | |
|  | ≥Grade 3 | 2 (0.15%) | 2 (0.15%) | 4 (0.15%) | 1.0000 | |
| Infectious pneumonia | Any | 1 (0.08%) | 0 | 1 (0.04%) | 1.0000 | |
|  | ≥Grade 3 | 1 (0.08%) | 0 | 1 (0.04%) | 1.0000 | |
| Bronchitis | Any | 1 (0.08%) | 0 | 1 (0.04%) | 1.0000 | |
| Pulpitis | Any | 1 (0.08%) | 0 | 1 (0.04%) | 1.0000 | |
| Viral myocarditis | Any | 1 (0.08%) | 0 | 1 (0.04%) | 1.0000 | |
|  | ≥Grade 3 | 1 (0.08%) | 0 | 1 (0.04%) | 1.0000 | |
| Upper respiratory infections | Any | 0 | 1 (0.08%) | 1 (0.04%) | 0.4998 | |
| Pericoronitis | Any | 0 | 1 (0.08%) | 1 (0.04%) | 0.4998 | |
| Tonsillitis | Any | 0 | 1 (0.08%) | 1 (0.04%) | 0.4998 | |
| Nasopharyngitis | Any | 0 | 1 (0.08%) | 1 (0.04%) | 0.4998 | |
| **Systemic and administration-site adverse events** | | | | | |  |
| Total | Any | 8 (0.61%) | 6 (0.46%) | 14 (0.54%) | 0.7900 | |
|  | ≥Grade 3 | 0 | 1 (0.08%) | 1 (0.04%) | 0.4998 | |
| Fever | Any | 3 (0.23%) | 3 (0.23%) | 6 (0.23%) | 1.0000 | |
|  | ≥Grade 3 | 0 | 1 (0.08%) | 1 (0.04%) | 0.4998 | |
| Thoracalgia | Any | 2 (0.15%) | 1 (0.08%) | 3 (0.11%) | 1.0000 | |
| Administration-site erythema | Any | 1 (0.08%) | 0 | 1 (0.04%) | 1.0000 | |
| Administration-site Itch | Any | 1 (0.08%) | 0 | 1 (0.04%) | .0000 | |
| Administration-site swelling | Any | 1 (0.08%) | 0 | 1 (0.04%) | 1.0000 | |
| Chest discomfort | Any | 1 (0.08%) | 0 | 1 (0.04%) | 1.0000 | |
| Armpit pain | Any | 1 (0.08%) | 0 | 1 (0.04%) | 1.0000 | |
| Administration-site Discoloration | Any | 0 | 1 (0.08%) | 1 (0.04%) | 0.4998 | |
| Fatigue | Any | 0 | 1 (0.08%) | 1 (0.04%) | 0.4998 | |
| **Neurological disorders** | | | | | |  |
| Total | Any | 4 (0.31%) | 3 (0.23%) | 7 (0.27%) | 1.0000 | |
| Dizziness | Any | 4 (0.31%) | 3 (0.23%) | 7 (0.27%) | 1.0000 | |
| **Musculoskeletal and connective tissue diseases** | | | | | |  |
| Total | Any | 3 (0.23%) | 5 (0.38%) | 8 (0.31%) | 0.5071 | |
| Joint pain | Any | 1 (0.08%) | 0 | 1 (0.04%) | 1.0000 | |
| Limb pain | Any | 1 (0.08%) | 1 (0.08%) | 2 (0.08%) | 1.0000 | |
| Back pain | Any | 1 (0.08%) | 1 (0.08%) | 2 (0.08%) | 1.0000 | |
| Limb discomfort | Any | 0 | 2 (0.15%) | 2 (0.08%) | 0.2497 | |
| Muscle spasm | Any | 0 | 1 (0.08%) | 1 (0.04%) | 0.4998 | |
| Spinal pain | Any | 0 | 1 (0.08%) | 1 (0.04%) | 0.4998 | |
| **Skin and subcutaneous tissue diseases** | | | | | |  |
| Total | Any | 3 (0.23%) | 3 (0.23%) | 6 (0.23%) | 1.0000 | |
|  | ≥Grade 3 | 1 (0.08%) | 0 | 1 (0.04%) | 1.0000 | |
| Itch | Any | 2 (0.15%) | 1 (0.08%) | 3 (0.11%) | 1.0000 | |
|  | ≥Grade 3 | 1 (0.08%) | 0 | 1 (0.04%) | 1.0000 | |
| Prurigo | Any | 1 (0.08%) | 0 | 1 (0.04%) | 1.0000 | |
| Erythra | Any | 0 | 1 (0.08%) | 1 (0.04%) | 0.4998 | |
| Urticaria | Any | 0 | 1 (0.08%) | 1 (0.04%) | 0.4998 | |
| **Injuries, poisoning, and operational complications** | | | | | |  |
| Total | Any | 3 (0.23%) | 3 (0.23%) | 6 (0.23%) | 1.0000 | |
|  | ≥Grade 3 | 0 | 2 (0.15%) | 2 (0.08%) | 0.2497 | |
| Injury | Any | 1 (0.08%) | 0 | 1 (0.04%) | .0000 | |
| Ligament sprains | Any | 1 (0.08%) | 0 | 1 (0.04%) | 1.0000 | |
| Rib fracture | Any | 0 | 1 (0.08%) | 1 (0.04%) | 0.4998 | |
| Limb injuries | Any | 0 | 1 (0.08%) | 1 (0.04%) | 0.4998 | |
| Tibia fracture | Any | 1 (0.08%) | 0 | 1 (0.04%) | 1.0000 | |
|  | ≥Grade 3 | 1 (0.08%) | 0 | 1 (0.04%) | 1.0000 | |
| Head injury | Any | 0 | 1 (0.08%) | 1 (0.04%) | 0.4998 | |
|  | ≥Grade 3 | 0 | 1 (0.08%) | 1 (0.04%) | 0.4998 | |
| **Physical examination** | | | | | |  |
| Total | Any | 2 (0.15%) | 2 (0.15%) | 4 (0.15%) | 1.0000 | |
| AST elevated | Any | 1 (0.08%) | 0 | 1 (0.04%) | 1.0000 | |
| Increased blood pressure | Any | 1 (0.08%) | 1 (0.08%) | 2 (0.08) | 1.0000 | |
| Elevated blood glucose | Any | 0 | 1 (0.08) | 1 (0.04%) | 0.4998 | |
| **Heart diseases** | | | | | |  |
| Total | Any | 3 (0.23%) | 1 (0.08%) | 4 (0.15%) | 0.6247 | |
|  | ≥Grade 3 | 2 (0.15%) | 1 (0.08%) | 3 (0.11%) | 1.0000 | |
| Ventricular extrasystoles | Any | 1 (0.08%) | 0 | 1 (0.08%) | 1.0000 | |
| Myocardial infarction | Any | 1 (0.08%) | 1 (0.08%) | 2 (0.08%) | 1.0000 | |
|  | ≥Grade 3 | 1 (0.08%) | 1 (0.08%) | 2 (0.08%) | 1.0000 | |
| Angina | Any | 1 (0.08%) | 0 | 1 (0.08%) | 1.0000 | |
|  | ≥Grade 3 | 1 (0.08%) | 0 | 1 (0.08%) | 1.0000 | |
| **Metabolic diseases** | | | | | | |
| Total | Any | 1 (0.08%) | 0 | 1 (0.08%) | 1.0000 | |
| Diabetes mellitus type 2 | Any | 1 (0.08%) | 0 | 1 (0.08%) | 1.0000 | |
|  | ≥Grade 3 | 1 (0.08%) | 0 | 1 (0.08%) | 1.0000 | |
| **The kidneys and urinary system diseases** | | | | | |  |
| Total | Any | 1 (0.08%) | 1 (0.08%) | 2 (0.08%) | 1.0000 | |
| Nephrolithiasis | Any | 1 (0.08%) | 1 (0.08%) | 2 (0.08%) | 1.0000 | |
| **The reproductive system and breast glands diseases** | | | | | |  |
| Total | Any | 0 | 1 (0.08%) | 1 (0.04%) | 0.4998 | |
| Breast pain | Any | 0 | 1 (0.08%) | 1 (0.04%) | 0.4998 | |
| **Mental illness** | | | | | |  |
| Total | Any | 0 | 1 (0.08%) | 1 (0.04%) | 0.4998 | |
| Insomnia | Any | 0 | 1 (0.08%) | 1 (0.04%) | 0.4998 | |
| **Benign, malignant, and unidentified tumors (including cystic and polypoid)** | | | | | |  |
| Total | Any | 0 | 1 (0.08%) | 1 (0.04%) | 0.4998 | |
| Uterine leiomyomas | Any | 0 | 1 (0.08%) | 1 (0.04%) | 0.4998 | |

Data are n (%). n = number of participants. % = proportion of participants. Any = all the participants with any grade adverse reactions or events. The analysis was based on the intervention-modified intention-to-treat cohort. *Calculated with χ² test or Fisher’s exact test.

# **Table S5. Serious adverse events occurred within 6 months after the booster vaccination**

|  | **Vaccine group (N=1308)** | **Placebo group (N=1307)** | **Total (N=2615)** | **P-value^*^** | |
| --- | --- | --- | --- | --- | --- |
| **Serious adverse events** | 7 (0.54%) | 2 (0.15%) | 9 (0.34%) | 0.1789 | |
| **Respirational, thoracic, and mediastinal disorders** | | | | |  |
| Total | 1 (0.08%) | 0 | 1 (0.04%) | 1.0000 | |
| Hemoptysis | 1 (0.08%) | 0 | 1 (0.04%) | 1.0000 | |
| **Injuries, poisoning, and operational complications** | | | | |  |
| Total | 1 (0.08%) | 1 (0.08%) | 2 (0.08%) | 1.0000 | |
| Tibia fracture | 1 (0.08%) | 0 | 1 (0.04%) | 1.0000 | |
| Head injury | 0 | 1 (0.08%) | 1 (0.04%) | 0.4998 | |
| **Heart diseases** | | | | |  |
| Total | 2 (0.15%) | 1 (0.08%) | 3 (0.11%) | 1.0000 | |
| Myocardial infarction | 1 (0.08%) | 1 (0.08%) | 2 (0.08%) | 1.0000 | |
| Angina | 1 (0.08%) | 0 | 1 (0.08%) | 1.0000 | |
| **Metabolic diseases** | | | | |  |
| Total | 1 (0.08%) | 0 | 1 (0.08%) | 1.0000 | |
| Diabetes mellitus type 2 | 1 (0.08%) | 0 | 1 (0.08%) | 1.0000 | |
| **Infectious and invasive diseases** | | | | | |
| Total | 2 (0.15%) | 0 | 2 (0.08%) | 1.0000 | |
| Infectious pneumonia | 1 (0.08%) | 0 | 1 (0.04%) | 1.0000 | |
| Viral myocarditis | 1 (0.08%) | 0 | 1 (0.04%) | 1.0000 | |

^*^ The p-value is calculated by the Fisher exact probability method.

# **Table S6. Neutralizing antibodies to SARS-CoV-2 XBB.1.5 before and after a heterogeneous boost vaccination.**

|  | **LVRNA012 group** | **Placebo group** | **P-value** |
| --- | --- | --- | --- |
| **Day 0** | | |  |
| **N** | 49 | 51 |  |
| **GMT** | 16.7 (10.9, 25.6) | 15.0 (9.8, 22.8) | 0.7127 |
| **Day 7** | | |  |
| **N** | 45 | 50 |  |
| **GMT** | 113.2 (83.1, 154.1) | 12.5 (8.4, 18.6) | <0.0001 |
| **Seroconversion rate (%)** | 75.6 (61.3, 85.8) | 4.0 (1.1, 13.5) | <0.0001 |
| **GMFI** | 6.5 (4.4, 9.8) | 0.8 (0.7, 1.1) | <0.0001 |
| **Day 14** | | |  |
| **N** | 42 | 48 |  |
| **GMT** | 132.3 (99.8, 175.4) | 12.5 (8.4, 18.7) | <0.0001 |
| **Seroconversion rate (%)** | 76.2 (61.5, 86.5) | 2.1 (0.4, 10.9) | <0.0001 |
| **GMFI** | 7.5 (5.0, 11.2) | 0.8 (0.7, 1.0) | <0.0001 |
| **Day 28** | | |  |
| **N** | 42 | 48 |  |
| **GMT** | 84.7 (64.4, 111.5) | 10.5 (7.1, 15.7) | <0.0001 |
| **Seroconversion rate (%)** | 57.1 (42.2, 70.9) | 2.1 (0.4, 10.9) | <0.0001 |
| **GMFI** | 4.8 (3.1, 7.3) | 0.7 (0.6, 0.8) | <0.0001 |
| **Day 90** | | |  |
| **N** | 42 | 48 |  |
| **GMT** | 44.5 (34.9, 56.8) | 7.7 (5.4, 11.0) | <0.0001 |
| **Seroconversion rate (%)** | 42.9 (29.1, 57.8) | 2.1 (0.4, 10.9) | <0.0001 |
| **GMFI** | 2.5 (1.6, 3.9) | 0.5 (0.4, 0.7) | <0.0001 |
| **Day 180** | | |  |
| **N** | 38 | 40 |  |
| **GMT** | 56.3 (36.7, 86.6) | 36.1 (20.5, 63.6) | 0.2130 |
| **Seroconversion rate (%)** | 47.4 (32.5, 62.7) | 42.5 (28.5, 57.8) | 0.6657 |
| **GMFI** | 2.9 (1.7, 5.1) | 1.9 (1.0, 3.5) | 0.2902 |

Data are GMT (95% CI), GMFI (95% CI) or the number of participants (%, 95%CI). N= the number of participants included in the intention-to-treat cohort. Measurements on day 0 were taken immediately before vaccination. GMT = geometric mean titer. GMFI = geometric mean fold increase. The Student-t test was used for comparison of GMTs and GMFIs.

# **Table S7. SARS-CoV-2 spike-specific cytokine T cells responses before and after a heterogeneous boost vaccination**

|  |  | **LVRNA012 group** | **Placebo group** | **P-value** |
| --- | --- | --- | --- | --- |
| **IFN-γ** |  |  |  |  |
| Day0 | N | 49 | 51 |  |
|  | Median (IQR) | 24.4 (15.6, 41.1) | 26.7 (18.9, 46.7) | 0.7018 |
| Day7 | N | 45 | 50 |  |
|  | Median (IQR) | 245.6 (113.3, 430.6) | 49.4 (20, 101.7) | <0.0001 |
| Day14 | N | 45 | 49 |  |
|  | Median (IQR) | 201.1 (118.3, 259.4) | 106.7 (65.0, 142.8) | <0.0001 |
| Day28 | N | 42 | 48 |  |
|  | Median (IQR) | 208.9 (113.1, 321.7) | 57.8 (39.2, 131.4) | <0.0001 |
| Day90 | N | 42 | 48 |  |
|  | Median (IQR) | 195.6 (97.8, 304.5) | 52.2 (31.7, 177.5) | <0.0001 |
| **IL-2** |  |  |  |  |
| Day0 | N | 49 | 51 |  |
|  | Median (IQR) | 13.3 (9.4, 23.3) | 12.2 (7.8, 21.1) | 0.2605 |
| Day7 | N | 45 | 50 |  |
|  | Median (IQR) | 180.0 (91.7, 387.5) | 45.0 (23.1, 185.0) | 0.0002 |
| Day14 | N | 45 | 49 |  |
|  | Median (IQR) | 117.8 (68.1, 203.3) | 57.8 (32.8, 107.8) | 0.0001 |
| Day28 | N | 42 | 48 |  |
|  | Median (IQR) | 307.8 (192.2, 395.0) | 173.9 (83.9, 264.2) | 0.0003 |
| Day90 | N | 42 | 48 |  |
|  | Median (IQR) | 165.6 (57.8, 271.1) | 57.8 (33.3, 145.8) | 0.0063 |
| **IL-4** |  |  |  |  |
| Day0 | N | 49 | 51 |  |
|  | Median (IQR) | 3.3 (1.1, 8.3) | 3.3 (1.1, 7.8) | 0.9778 |
| Day7 | N | 45 | 50 |  |
|  | Median (IQR) | 120.0 (52.2, 188.9) | 24.4 (10.8, 43.9) | <0.0001 |
| Day14 | N | 45 | 49 |  |
|  | Median (IQR) | 71.1 (35.6, 97.8) | 26.7 (16.1, 44.4) | <0.0001 |
| Day28 | N | 42 | 48 |  |
|  | Median (IQR) | 72.2 (45.0, 136.1) | 21.1 (10.0, 63.1) | <0.0001 |
| Day90 | N | 42 | 48 |  |
|  | Median (IQR) | 30.0 (16.7, 49.4) | 9.4 (4.4, 29.7) | <0.0001 |
| **IL-13** |  |  |  |  |
| Day0 | N | 49 | 51 |  |
|  | Median (IQR) | 16.7 (8.3, 43.3) | 18.9 (10.0, 64.4) | 0.5056 |
| Day7 | N | 45 | 50 |  |
|  | Median (IQR) | 47.2 (23.6, 96.4) | 42.8 (17.5, 69.2) | 0.4448 |
| Day14 | N | 45 | 49 |  |
|  | Median (IQR) | 51.7 (26.4, 78.6) | 40.0 (16.1, 53.3) | 0.0636 |
| Day28 | N | 42 | 48 |  |
|  | Median (IQR) | 18.9 (9.4, 39.4) | 9.4 (4.4, 21.1) | 0.0086 |
| Day90 | N | 42 | 48 |  |
|  | Median (IQR) | 8.9 (3.3, 19.4) | 4.4 (1.1, 10.8) | 0.0112 |
| **Th1/Th2 ratio** |  |  |  |  |
| Day0 | N | 49 | 51 |  |
|  | Median (IQR) | 1.6 (0.8, 4.0) | 1.8 (0.7, 4.0) | 0.3819 |
| Day7 | N | 45 | 50 |  |
|  | Median (IQR) | 2.8 (1.6, 3.7) | 2.0 (1.0, 3.7) | 0.1144 |
| Day14 | N | 45 | 49 |  |
|  | Median (IQR) | 2.8 (2.0, 3.5) | 2.5 (1.6, 4.0) | 0.5235 |
| Day28 | N | 42 | 48 |  |
|  | Median (IQR) | 5.1 (4.1, 8.0) | 7.0 (3.9, 11.2) | 0.2688 |
| Day90 | N | 42 | 48 |  |
|  | Median (IQR) | 9.0 (4.7, 12.5) | 8.2 (4.4, 15.2) | 0.2090 |

Samples of PBMCs were collected from the first 50 participants in the two treatment groups and included in the analysis. PBMCs=peripheral blood mononuclear cells. Th1/Th2 ratios were calculated by summing IFN-γ and IL-2 cytokine levels and then dividing by the sum of IL-4 and IL-13 cytokine levels.

# **Figure S1. Cumulative Incidence of COVID-19 Incident Cases (1 – Kaplan-Meier Estimate) 7 days following the vaccination of the LVRNA012 vaccine or the placebo.**


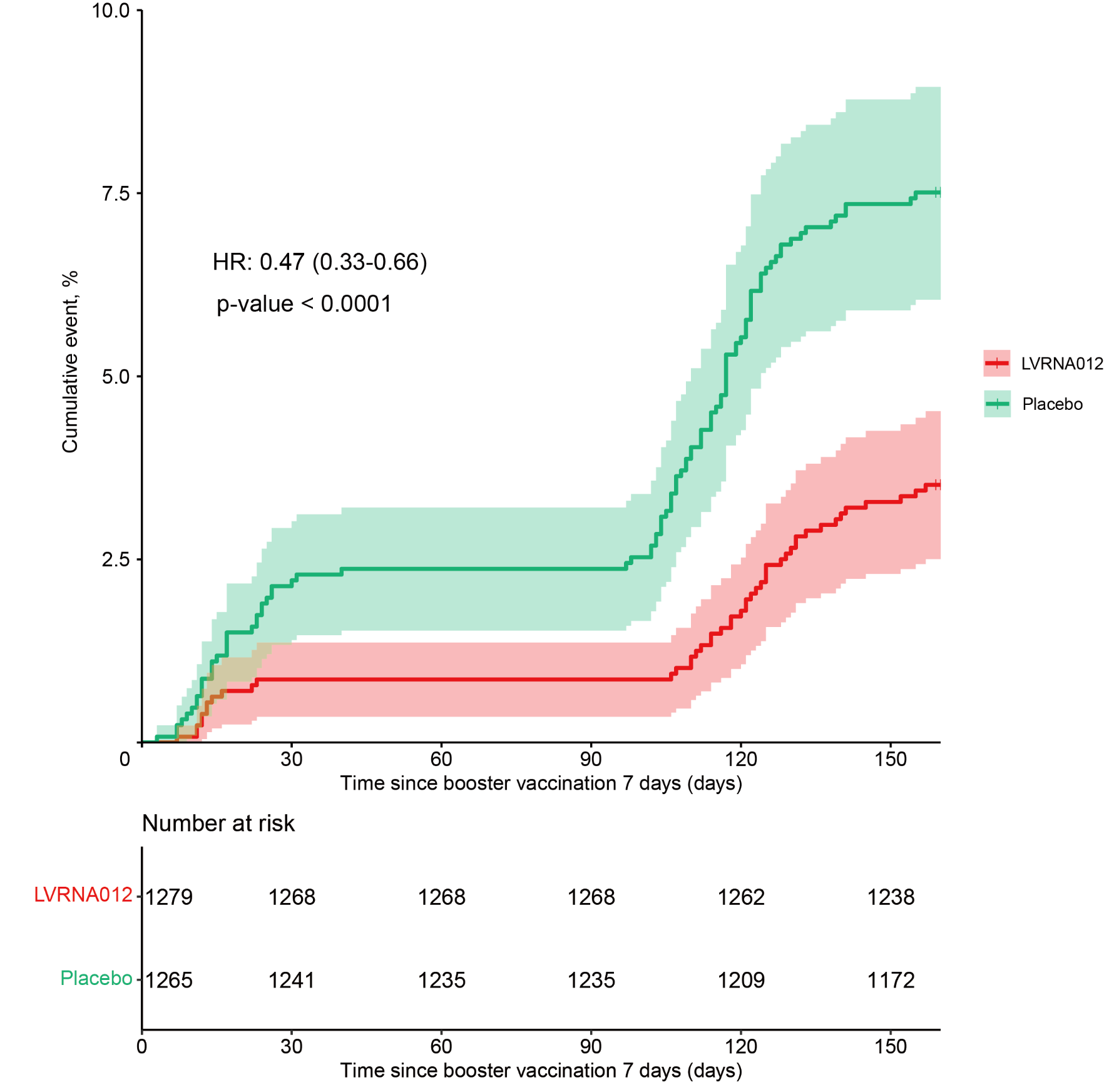


Shown is the cumulative incidence curve of the first COVID-19 occurrence after the vaccination of the LVRNA012 vaccine or the placebo, as calculated employing the Kaplan–Meier method. The shading represents 95% confidence intervals. Each symbol represents the onset of a COVID-19 case.

# **Figure S2.** **Cumulative Incidence of COVID-19 Incident Cases (1 – Kaplan-Meier Estimate) 7 days following the vaccination of the LVRNA012 vaccine or the placebo in participants who had previously received two or three doses of inactivated vaccine.**


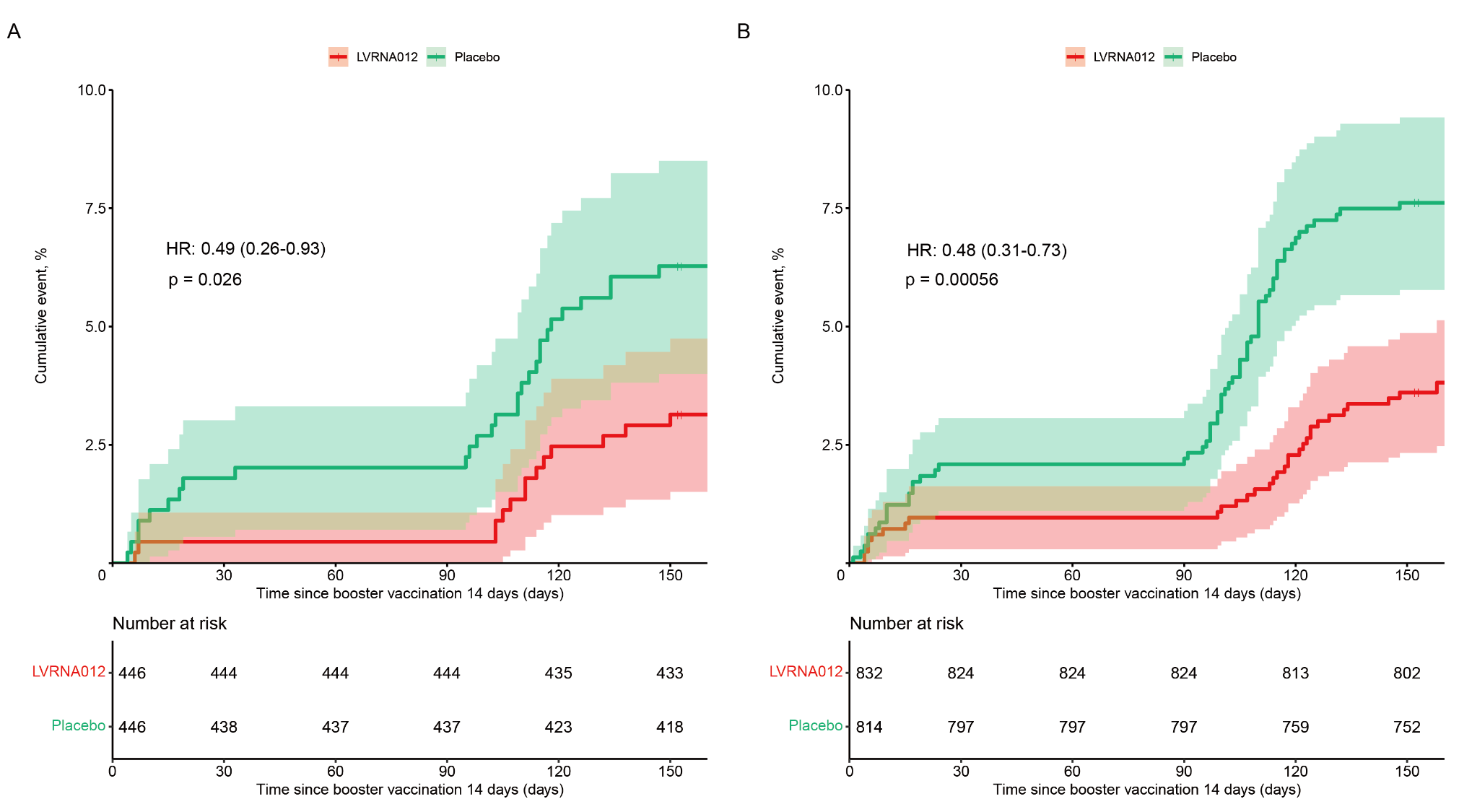


(A) Cumulative Incidence of COVID-19 Incident Cases (1 – Kaplan-Meier Estimate) 7 days following the vaccination of the LVRNA012 vaccine or the placebo in participants who had previously received two doses of inactivated vaccine. (B) Cumulative Incidence of COVID-19 Incident Cases (1 – Kaplan-Meier Estimate) 7 days following the vaccination of the LVRNA012 vaccine or the placebo in participants who had previously received three doses of inactivated vaccine. Shown is the cumulative incidence curve of the first COVID-19 occurrence after the vaccination of the LVRNA012 vaccine or the placebo, as calculated employing the Kaplan–Meier method. The shading represents 95% confidence intervals. Each symbol represents the onset of a COVID-19 case.

# **Figure S3. Trends of mutant strains in native cases of COVID-19 infections in China.**


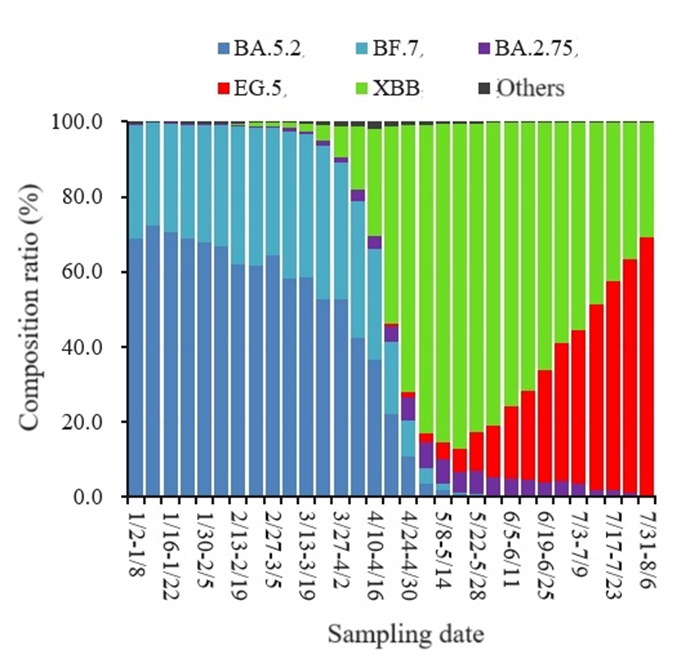


Data from China CDC's "National Epidemic of Novel Coronavirus Infections" (Accessed 2023-12-12). Website: https://www.chinacdc.cn/jkzt/crb/zl/szkb_11803/jszl_13141/202312/t20231212_271320.html.

# **Figure S4. Trends in COVID-19 of influenza-like illnesses and influenza virus positivity rates in China.**


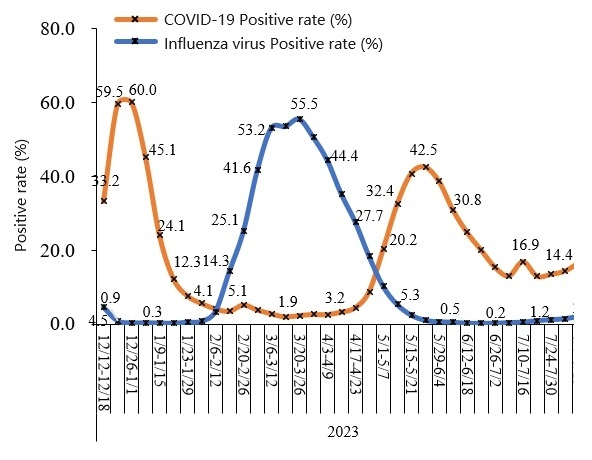


Data from China CDC's "National Epidemic of Novel Coronavirus Infections" (Accessed 2023-12-12). Website: https://www.chinacdc.cn/jkzt/crb/zl/szkb_11803/jszl_13141/202312/t20231212_271320.html.
