# Supplementary material for: Efficacy, safety, and immunogenicity of SARS-CoV-2 mRNA vaccine (Omicron BA.5) LVRNA012: a randomized, double-blind, placebo-controlled phase 3 trial
Source: Front Immunol. 2024 Jun 6;15:1407826. doi: 10.3389/fimmu.2024.1407826 (PMC11187303; doi:10.3389/fimmu.2024.1407826)
Supplement: Supplementary file 2 [file DataSheet_2.pdf]

A Single-Center, Randomized, Blinded, Placebo-Controlled Clinical Study to Evaluate the Efficacy, Safety, and Immunogenicity of a SARS-CoV-2 Variant (Omicron BA.5) mRNA Vaccine (LVRNA012) Vaccine Administered as 1 Dose in adults Aged 18 Years and Older Who Have Completed 2 or 3 Doses of SARS-CoV-2 Inactivated Vaccine.

**Study Vaccine:**	SARS-CoV-2 mRNA vaccine (Omicron BA.5) LVRNA012
**Protocol Number:**	LVRNA012-IIT-04
**Version Number:**	2.1
**Version Date:**	08 February 2023
**Research institution:**	The First Affiliated Hospital of Bengbu Medical College
**Funder：**	AIM Vaccine Co., Ltd.<br>Ningbo Rongan Biological Pharmaceutical Co., Ltd.

Content

Signature Page of Principal Investigator 5
Abbreviations and Acronyms 6
Glossary of terms 8
Protocol Synopsis 9
Research Team 29
1. Study Title 31
2. Introduction 31
3. Background and rationale 33
3.1. Pathogenetic Background 33
3.2. Disease Background 34
3.3. Vaccine Brief 37
4. Background of the SARS-CoV-2 variant (Omicron BA.5) mRNA vaccine (LVRNA012) study 39
4.1. Theoretical foundations 39
4.2. Risk Assessment 40
4.3. Benefits assessment 42
4.4. Benefit/risk assessment 43
4.5. Summary 44
5. Participants 44
6. Study Design 44
6.1. Study objective(s) 44
6.2. Study endpoint(s) 46
6.3. Overall Design 49
6.4. Study hypothesis 49
6.5. Sample size and groups 49
6.6. Observation of protective efficacy 51
6.7. Safety observations 54
6.8. Immunogenicity observation (for immunogenicity subgroup only) 54
6.9. Follow-up plan 54
6.10. Data collection 55
6.11. Randomization and Blinding 55
6.12. Study suspension or termination criteria 58
6.13. Confidentiality agreement and privacy of the participants 58
6.14. Study Duration 59
7. Study population 59
7.1. Inclusion criteria 59
7.2. Exclusion criteria 60
7.3. The criteria for early termination of the study: 61
7.4. Participant withdrawal from the study: 62
8. Lost to follow-up, protocol deviation/violation, pregnancy 63
9. Vaccines, vaccine transportation, storage and administration 65

9.1. Investigational technology 65
9.2. Storage and Transportation of Vaccines 66
9.3. Route of Vaccination and Immunization Procedure 66
9.4. Backup vaccine 66
9.5. Vaccine number. 66
9.6. Vaccine allocation 67
9.7. Vaccine packaging and labeling 67
9.8. Lost to follow-up 63
9.9. Protocol deviation/protocol violation 64
9.10. Pregnancy 64
10. Concomitant medication: 67
11. Study methods and procedures 68
11.1. Participant Recruitment 68
11.2. Study procedure 69
11.3. Methods of observation of protective efficacy 74
11.4. Safety observation method 77
11.5. Immunogenicity observation method 83
11.6. Biospecimen management 84
11.7. Data management 85
11.8. Statistical analysis 87
12. Subject Safety and adverse event management 92
12.1.Adverse Event Definition 92
12.2. Security precaution 93
12.3. Discovery and Collection of Adverse Events 94
12.4. Treatment and Management of Adverse Events 94
12.5. Serious Adverse Event Reporting 95
12.6. Reporting of AESI 99
13. Quality assurance and control of Clinical research 100
13.1.Investigator 100
13.2.Funder 101
13.3.Site Monitor 102
13.4.Blood sample management 103
13.5.Vaccine management 104
13.6.Research data 105
13.7.Publication of research results 106
14. Clinical medical research ethics committee 106
14.1. Ethical standards 106
14.2. Ethical review 106
15. Confidentiality 107
Appendix 1 108
1. Secondary efficacy end point severe/critical COVID-19 case definition and diagnostic criteria 109
2. Diagnosis and Treatment Protocol for COVID-19 (Trial Version 10) issued by NMPA110
16. References 111

Signature Page of Principal Investigator

**Protocol Number**	LVRNA021-IIT-04
**Version Number**	2.1
**Version Date**	08 February 2023
**Study Title**	A Single-Center, Randomized, Blinded, Placebo-Controlled Clinical Study to Evaluate the Efficacy, Safety, and Immunogenicity of a SARS-CoV-2 Variant (Omicron BA.5) mRNA Vaccine (LVRNA012) Vaccine Administered as 1 Dose in adults Aged 18 Years and Older Who Have Completed 2 or 3 Doses of SARS-CoV-2 Inactivated Vaccine.

Signature of Principal Investigator

Institution: The First Affiliated Hospital of Bengbu Medical College

Signature:

Date:

Abbreviations and Acronyms

Abbreviation	Definition
ADE	Antibody-Dependent Enhancement
AE	Adverse Event
AESI	Adverse Event of Special Interest
CI	Confidence Interval
CoV	Coronavirus
COVID-19	Coronavirus Disease 2019
CRO	Contract Research Organization
eCRF	Electronic Case Report Form
ECMO	Extracorporeal Membrane Oxygenation
EDC	Electronic Data Capture
ELISA	Enzyme-linked Immunosorbent Assay
ELISpot	Enzyme-linked Immunosorbent Spot
EOS	End of Study
FiO_2	Fraction of inspired oxygen
GMI	Geometric Mean Increase
GMT	Geometric Mean Titer
IFN-γ	Interferon-γ
IgG	Immunoglobulin G
IgM	Immunoglobulin M
IL	Interleukin
ITT	Intent to treat
LLOQ	Lower Limit of Quantification
MedDRA	Medical Dictionary for Regulatory Activities
MERS	Middle East Respiratory Syndrome
NMPA	National Medical Products Administration
PaO_2	Partial pressure of oxygen

Abbreviation	Definition
PCR	Polymerase Chain Reaction
RT-PCR	Reverse Transcription-Polymerase Chain Reaction
SAE	Serious Adverse Event
SARS-CoV-2	Severe Acute Respiratory Syndrome Coronavirus 2
SBP	Systolic Pressure
SCR	Seroconversion Rate
SOP	Standard Operational Procedure
SUSAR	Suspected Unexpected Serious Adverse Reaction
VE	Vaccine Effectiveness
VED	Vaccine-Enhanced Disease
VOC	Variants of Concern
VUM	Variant Under Monitoring
WHO	World Health Organization

Glossary of terms

Adverse Event (AE): it refers to all adverse medical events that occur after participants receive the vaccine/drug for the study, which can be manifested as symptoms, signs, diseases, or laboratory test abnormalities, but are not necessarily related to the study vaccine/drug.

Serious Adverse Event (SAE): it refers to the adverse medical event that causes death, is life-threatening, leads to permanent or serious disability or incapability, requires hospitalization or prolonged hospitalization, and results in congenital abnormalities or birth defects after the participants receive the study drug/vaccine.

Investigational Vaccine: Investigational vaccines, control vaccines for clinical studies.

Audit: audit refers to the systematic and independent inspection of clinical trial-related activities and documents to assess and determine whether the implementation of clinical trial-related activities, recording, analyzing, and reporting of trial data comply with the trial protocols, standard operating procedures, and relevant laws and regulations.

Participant: a person, including a patient, a healthy subject, who participates in a clinical trial and is inoculated as a drug for the trial.

Solicited Adverse Event: A solicited adverse event is an adverse event that is collected as a safety endpoint in a clinical study and refers to information on an adverse event that is collected on the investigator's or participant's initiative during a specific follow-up time after vaccination; it is usually related to the investigational vaccine and is listed in advance in the protocol and CRF.

Unsolicited Adverse Event: unsolicited adverse events are adverse events other than solicited adverse events reported in a clinical study. Also includes solicited symptoms reported outside of the designated solicitation time.

Protocol Synopsis

**Study Title**	A Single-Center, Randomized, Blinded, Placebo-Controlled Clinical Study to Evaluate the Efficacy, Safety, and Immunogenicity of a SARS-CoV-2 Variant (Omicron BA.5) mRNA Vaccine (LVRNA012) Vaccine Administered as 1 Dose in adults Aged 18 Years and Older Who Have Completed 2 or 3 Doses of SARS-CoV-2 Inactivated Vaccine.
**Technical Introduction**	This vaccine consists of mRNA molecules encoding the full length of the SARS-CoV-2 mutant Omicron BA.5 echinocandin S protein combined with lipids to form lipid nanoparticles. After thawing, the vaccine is a clear, slightly milky white liquid.
**Indications**	Prevention of COVID-19 caused by SARS-CoV-2
**population**	People aged 18 years and older who have completed 2 or 3 doses of SARS-CoV-2 inactivated vaccine.
**Sample Size**	Approximately 3,200 participants
**Introduction**	COVID-19 has continued to rage around the world since its outbreak in December 2019. The outbreak was declared a Public Health Emergency of International Concern on 30 January 2020. On 11 February 2020, the virus was officially named severe acute respiratory syndrome coronavirus-2 (SARS-CoV-2), and the WHO officially named the disease caused by SARS-CoV-2 as coronavirus disease 2019 (COVID-19). SARS-CoV-2 infections and the resulting disease, COVID-19, have spread globally, and on 11 March 2020, the WHO declared the COVID-19 outbreak as a pandemic. WHO data showed that as of 28 October 2022, 224 countries or regions worldwide have reported a cumulative total of more than 625 million confirmed cases and more than 6.5 million deaths. The outbreak and epidemic of COVID-19 have put heavy economic pressure and medical burden on people worldwide and pose a serious threat to human survival and health.

	Vaccination is considered one of the most effective interventions to prevent COVID-19, as it reduces all types of complications and disease burden, and interrupts the transmission of the virus. WHO data showed that as of 13 Dec 2022, 374 vaccine candidates against SARS-CoV-2 are under development worldwide (about 175 are in clinical study) including various technical routes such as nucleic acid-based vaccines (mRNA and DNA), inactivated vaccines, viral vector vaccines (replicating or non-replicating), recombinant subunit vaccines and live attenuated vaccines. Numerous COVID-19 vaccines are currently in development globally, and several candidate COVID-19 vaccines are efficacious in the prevention of COVID-19 in clinical studies and are now available under temporary or emergency authorizations. However, no self-developed COVID-19 mRNA vaccine has been approved for use in China yet.<br><br>Further booster vaccinations for vaccinated populations have brought widespread global attention as the emergence of SARS-CoV-2 variants and the decline in antibody levels over time after vaccination. According to a joint release from Pfizer and BioNTech on July 09, 2022, their ongoing trial of the third dose of booster vaccination with a SARS-CoV-2 mRNA vaccine showed positive results. The results showed that subjects who received a third dose of booster vaccine six months after receiving two doses of the COVID-19 mRNA vaccine (BNT162b2) showed high neutralizing titers in sera against the wild-type strain and Beta variant strain, which is 5- to 10-fold increases in neutralizing antibody titers compared to subjects who received only two doses of the vaccine, also showed prior consistent tolerability profile and safety profile. Sequential vaccination using different technology pathways is expected to increase the strength, breadth, and durability of the immune response through complementarity between different mechanisms, resulting in more comprehensive and potent protection. A study in Turkey showed that booster immunization with mRNA vaccine after completion of 2 doses of inactivated vaccine induced 27 times higher levels of IgG antibodies compared with booster immunization with the 3rd dose of inactivated vaccine homologue.

	Because the Omicron variant is highly transmissible, has a greater ability to evade immunization despite its reduced pathogenicity. That resulted in most of the currently marketed vaccines developed by the original strain having low protective efficacy against the disease caused by this variant. The development of new vaccines against the variant, as well as new vaccination protocols, has become particularly important.<br><br>Since the outbreak of COVID-19, the CPC Central Committee and the State Council have optimized the prevention and control measures according to the characteristics of the virus mutation and the development of the epidemic. Recently, they have optimized and put forward "20" and "10" targeted measures to continuously improve the level of scientific precision in prevention and control, maximize the protection against the disease, minimize the impact of the epidemic on people's production and living order and economic and social development. With the gradual liberalization of epidemic prevention and control as well as the acceleration of economic construction, the movement of people, and the increase of social activities, China facing greater pressure in epidemic prevention and control work especially in the medical resources. Therefore, in the period after three doses of the new crown vaccine to strengthen the vaccination of the fourth dose has become a necessary measure for the control of the new crown epidemic. Meanwhile, based on the vaccination status of the marketed SARS-CoV-2 vaccine in China and the consensus on the advantages of sequential booster immunization in both domestic and international studies, it is of great significance to carry out a clinical study on sequential booster immunization in the population that has already completed 2 or 3 doses of inactivated SARS-CoV-2 vaccine.<br><br>This study is a clinical research of a SARS-CoV-2 variant mRNA vaccine (LVRNA012). The study was single-center, randomized, blinded, and placebo-controlled. The protective efficacy, safety, and immunogenicity of the study vaccine were evaluated with 1 dose of the mRNA vaccine (LVRNA021) as a booster in participants aged 18 years and older who had completed 2 or 3 doses of

	the vaccine.
**Investigational Vaccine**	**Study vaccine: SARS-CoV-2 mRNA vaccine (LVRNA021)**<br>Provided by: AIM Vaccine Co., Ltd.<br>Dosage: 100 μg /1.0 mL/dose<br>Appearance: colorless or slightly milky white liquid<br>Dosage form: Injection<br>Route of administration: intramuscular injection into the lateral deltoid muscle of the upper arm<br>Vaccination procedure: 1 dose<br>Storage and transport conditions: Should be stored and transported at -20°C ± 5°C, protected from light.<br>**Placebo: Saline** (0.9% sodium chloride solution)<br>Provided by: AIM Vaccine Co., Ltd.<br>Dosage: 0.5 mL/dose<br>Appearance: colorless clear liquid<br>Dosage form: Injection<br>Route of administration: intramuscular injection into the lateral deltoid muscle of the upper arm<br>Vaccination procedure: 1 dose<br>Storage and transport conditions: Should be stored and transported at room temperature.
**Study Objective(s)**	**Primary objective(s)**<br>➢ The primary objective of protective efficacy<br>1)To evaluate the protective efficacy of the investigational vaccine or placebo in the prevention of symptomatic COVID-19 cases of any severity (Appendix 1) occurring from 14 days after booster vaccination in adults aged 18 and older who have

	completed 2 or 3 doses of inactivated COVID-19 vaccines.<br><br>**Secondary objective(s)**<br><br>➢ Secondary objective(s) of protective efficacy<br><br>1)To observe the protective efficacy of the investigational vaccine or placebo in the prevention of severe and critical COVID-19 cases (Appendix 1) from 14 days after booster vaccination in adults aged 18 and older who have completed 2 or 3 doses of inactivated COVID-19 vaccines.<br><br>2)To observe the protective efficacy of the investigational vaccine or placebo in the prevention of COVID-19 cases leading to death (Appendix 1) from 14 days after booster vaccination in adults aged 18 and older who have completed 2 or 3 doses of inactivated COVID-19 vaccines.<br><br>➢ Secondary objective(s) of safety<br><br>1)To evaluate the safety of the investigational vaccine or placebo within 28 days after booster vaccination in adults aged 18 and older who have completed 2 or 3 doses of inactivated COVID-19 vaccines.<br><br>2)To observe the safety of the investigational vaccine or placebo within 6 months after booster vaccination in adults aged 18 and older who have completed 2 or 3 doses of inactivated COVID-19 vaccines.<br><br>➢ Secondary objective(s) of immunogenicity<br><br>1)To evaluate the SARS-CoV-2 virus-neutralizing antibody against the main epidemic strains among the immunogenicity subgroup 7 days, 14 days, 28 days, 3 months, and 6 months after booster vaccination of the investigational vaccine or placebo.<br><br>2)To evaluate the SARS-CoV-2 S-protein IgG antibody against the main epidemic strains among the immunogenicity subgroup 7 days, 14 days, 28 days, 3 months, and 6 months after booster vaccination of the investigational vaccine or placebo.

	**Exploratory objective(s)**<br>➢ Exploratory objective(s) of protective efficacy<br>1)To observe the protective efficacy of the investigational vaccine or placebo in the prevention of symptomatic COVID-19 cases of any severity (Appendix 1) occurring from 7 days after booster vaccination in adults aged 18 and older who have completed 2 or 3 doses of inactivated COVID-19 vaccines.<br>➢ Exploratory objective(s) of immunogenicity<br>1)To observe the specific cellular immune response among the immunogenicity subgroup 7 days, 14 days, 28 days, and 3 months after booster vaccination of the investigational vaccine or placebo in the adults aged 18 and older who have completed 2 or 3 doses of inactivated COVID-19 vaccines.<br>To explore the relationship between neutralizing antibody, S-protein IgG antibody, and/or cellular immunity and vaccine protective efficacy after vaccination.
**Study endpoint(s)**	**Primary endpoint(s)**<br>➢ The primary endpoint(s) of protective efficacy<br>1)The person-year incidence rate of symptomatic COVID-19 cases of any severity (Appendix 1) occurring from 14 days after booster vaccination of the investigational vaccine or placebo in adults aged 18 and older who have completed 2 or 3 doses of inactivated COVID-19 vaccines.<br>**Secondary endpoint(s)**<br>➢ Secondary endpoint(s) of protective efficacy<br>1)The person-year incidence rate of severe and critical COVID-19 cases (Appendix 1) occurring from 14 days after booster vaccination of the investigational vaccine or placebo in the adults aged 18 and older who have completed 2 or 3 doses of inactivated COVID-19 vaccines.<br>2)The person-year incidence rate of COVID-19 cases leading to death (Appendix 1)

	occurring from 14 days after booster vaccination of the investigational vaccine or placebo in adults aged 18 and older who have completed 2 or 3 doses of inactivated COVID-19 vaccines.<br><br>➢ Secondary endpoint(s) of immunogenicity<br><br>1)The geometric mean titer (GMT), geometric mean increase (GMI), and seroconversion rate (SCR) of SARS-CoV-2 virus neutralizing antibody against the main epidemic strains among the immunogenicity subgroup 7 days, 14 days, 28 days, 3 months and 6 months after booster vaccination of the investigational vaccine or placebo.<br><br>2)The geometric mean titer (GMT), geometric mean increase (GMI), and seroconversion rate (SCR) of SARS-CoV-2 S-protein IgG antibody against the main epidemic strains among the immunogenicity subgroup 7 days, 14 days, 28 days, 3 months and 6 months after booster vaccination of the investigational vaccine or placebo.<br><br>➢ Secondary endpoint(s) of safety<br><br>1)The incidence of AEs within 30 mins, 14 days, and 28 days after booster vaccination of the investigational vaccine or placebo in adults aged 18 and older who have completed 2 or 3 doses of inactivated COVID-19 vaccines.<br><br>2)The incidence of SAEs and AESIs within 6 months after booster vaccination of the investigational vaccine or placebo in adults aged 18 and older who have completed 2 or 3 doses of inactivated COVID-19 vaccines.<br><br>3)The pregnancy events (including the pregnancy outcome, the delivery characteristics, the condition of the newborn, and the growth and development within 1 month after birth) within 6 months after booster vaccination of the investigational vaccine or placebo in the adults aged 18 and older who have completed 2 or 3 doses of inactivated COVID-19 vaccines.<br><br>**Exploratory endpoint(s)**

	➢ Exploratory endpoint(s) of protective efficacy<br>1)The person-year incidence rate of symptomatic COVID-19 cases of any severity (Appendix 1) occurring from 7 days after booster vaccination of the investigational vaccine or placebo in the adults aged 18 and older who have completed 2 or 3 doses of inactivated COVID-19 vaccines.<br>➢ Exploratory endpoint(s) of immunogenicity<br>1)The specific cellular immune response detected via cytokines IL-2, IL-4, IL-13, IFN-γ (ELISpot) among the immunogenicity subgroup 7 days, 14 days, 28 days, and 3 months after booster vaccination of the investigational vaccine or placebo.<br>2)The correlation coefficient between neutralizing antibody, S-protein IgG antibody, and/or cellular immunity and vaccine protective efficacy after vaccination.
**Study suspension or termination criteria**	The sponsor reserves the right to suspend/terminate the study at any time. After full consultation with the sponsor and the sponsor's consent, the investigator could suspend or terminate the work of the research center. Reasons for suspending or terminating the study may be, but are not limited to, the followings:<br>(5)It is clear that the investigational vaccine lacks efficacy;<br>(6)The incidence or severity of adverse reactions indicate that there may be potential dangers that may endanger the life of the participants;<br>(7)The sponsor suspends or terminates the research voluntarily;<br>(8)Suspend or terminate due to regulatory agency requirements.<br>During the study, if any of the criteria triggering the study suspension occurs, the safety of the investigational vaccine will be assessed by the investigator.
**Study Design**	**Overall Design**<br>A single-center, randomized, blinded, placebo-controlled design will be used for this study.<br>**Sample Size Allocation**

This study plans to enroll approximately 3,200 participants who have completed 2 or 3 doses of inactivated COVID-19 vaccine for ≥ 6 months (Study No. 0001~3200). All participants with informed consent, physical examination, and screening will be 1:1 randomly assigned into the experimental group or the control group (1,600 participants in each group) and followed up for safety and protective efficacy.

In this study, the first 50 participants enrolled in each group (a total of 100 participants) will be set as the immunogenicity subgroup (Study No. 0001~0100). All participants enrolled need to be followed up for safety and protective efficacy after vaccination. The participants in the immunogenicity subgroup also need to collect blood for the detection of immunogenicity-related indicators before vaccination and 7 days, 14 days, 28 days, 3 months, and 6 months after vaccination. Extra blood will be collected for cellular immunity test before vaccination and at days 7, 14, 28, and month 3 after vaccination.

The groups, vaccines, and planned sample size are detailed in the table below:

<table>
<tr><th>Participants</th><th>Groups</th><th>Vaccines</th><th>Immunogenicity subgroup</th><th>Non-immunogenicity subgroup</th><th>Sample size(no.)</th></tr>
<tr><td rowspan="3">Adults aged 18 and older who have been inoculated with 2 or 3 doses of inactivated COVID-19 vaccines for ≥6 months</td><td>Study group</td><td>LVRNA012</td><td>50</td><td>1550</td><td>1,600</td></tr>
<tr><td>Control group</td><td>Placebo</td><td>50</td><td>1550</td><td>1,600</td></tr>
<tr><td colspan="2">Total</td><td>100</td><td>3100</td><td>3,200</td></tr>
</table>

	**Observation of protective efficacy**<br><br>**7 days after the booster vaccination for every participant, the investigators need to carry out the following "routine monitoring" on the participants through a combination of remote visits and on-site visits:**<br><br>➢ The investigators remotely visit the participants to see if they have symptoms related to COVID-19;<br><br>➢ When the investigator learns that the participant has any one or more of the following conditions through remote visits, it is necessary to collect the participant’s throat swab sample A1 (for SARS-CoV-2 nucleic acid or antigen detection), and further inquiry, record, and guidance should be done to the participant to continue to observe and record symptom types, start and end time, severity, etc. If an on-site visit is conducted to the participant (the on-site visit could be carried out by the participant going to the study site or the investigators' visit), another nucleic acid throat swab sample B1 (backup sample) needs to be collected:<br><br>*① Any possible signs or symptoms related to COVID-19 appear(fever or chills, malaise/fatigue, headache, dry throat, muscle pain, sore throat, nasal congestion/runny nose, anorexia/nausea/vomiting, diarrhea, new sense of smell/ dysgeusia, conjunctivitis);*<br>*② Any one or more respiratory-related symptoms (cough, shortness of breath, or dyspnea) appear and last for any time;*<br>*③ Clinical or imaging evidence of pneumonia.*<br><br>**If the result of SARS-CoV-2 nucleic acid or antigen detection of the participant's throat swab sample A1 is positive, the investigators need to continue to carry out the following "case monitoring" on the participant:**<br>➢ Send the participant’s nucleic acid throat swab sample B1 (if collected) to

	the central laboratory for SARS-CoV-2 variant strain type detection, and guide the participant to go to the designated hospital for chest imaging examination (chest CT) ;<br>➢ Provide necessary medical supplies to the participant, and continue to remotely visit him/her twice a week. Instruct the participant to observe and record symptoms and self-medication at home, or guide him/her to go to the designated hospitals for medical treatment according to the situation. If the participant has symptoms such as shortness of breath or dyspnea, guide him/her to detect blood oxygen saturation according to the situation. According to the symptoms recovery of the participant, conduct an on-site visit to collect a throat swab sample or antigen nasopharyngeal swab sample for SARS-CoV-2 nucleic acid or antigen detection, until the result of SARS-CoV-2 nucleic acid or antigen turns negative or the symptoms disappear or stabilize under the investigators' evaluation.<br>➢ Collect the participant's treatment and medical history to judge the severity of COVID-19, and make the records about the type, starting time, and reporting time of the participant's symptom, and the collection time, detection time, and reporting time of each nucleic acid sample, and the diagnosis time and outcome of the case, to form relevant records;<br>➢ Establish a COVID-19 case file for the participant, and fill in and report the first/follow-up COVID-19 case report according to the remote visit;<br>➢ On-site monitoring and medical monitoring teams need to conduct a preliminary review of the completeness, rationality, and logic of the COVID-19 case report, and raise necessary inquiries and requirements of information supplementary, then submit them to the investigators for diagnosis and judgment.<br>➢<br>**If the result of SARS-CoV-2 nucleic acid or antigen detection in the participant's throat swab sample A1 is negative, the following retest should be continued:**

	➢ The participant's throat swab sample A2 (for SARS-CoV-2 nucleic acid or antigen detection) and sample B2 (backup sample) need to be collected again within 48 hours;<br>➢ If the result of SARS-CoV-2 nucleic acid or antigen detection of the participant's throat swab sample A2 is positive, the above "case monitoring" process should be followed to carry out relevant work;<br>➢ If the result of SARS-CoV-2 nucleic acid or antigen detection of the participant's throat swab sample A2 is still negative, the above "routine monitoring" process should be continued to carry out relevant work.<br><br>**Frequency and methods of "visits":**<br>➢ From the 7th day after the booster vaccination, remote visits will be conducted at a frequency of twice a week (2-3 days' intervals is recommended), until the 90th day after vaccination: (1) If the number of confirmed primary endpoint cases reaches 162, the frequency of visits will be adjusted to once a month, until the 180th day after vaccination; (2) If the number of confirmed primary endpoint cases does not reach 162, remote visits will still be conducted twice a week. When the number reaches 162, the visit frequency will be adjusted to once a month, until the 180th day after vaccination;<br>➢ If the participant has a positive result of SARS-CoV-2 nucleic acid or antigen detection, no matter what stage he/she is in, he/she will be visited twice a week (2-3 days' interval is recommended), until his/her nucleic acid or antigen detection result turns negative or the symptoms disappear or stabilize under the investigator's assessment;<br>➢ Remote follow-up methods include text messages, phone calls, videos, photos, etc. On-site visits are carried out according to the above monitoring process and actual conditions.<br><br>**Safety observations**<br>All participants need to be followed up for safety:

	➢ **Safety observation from 0 to 28 days after booster vaccination**<br>All participants will collect all adverse events occurring within 30 minutes after vaccination, solicited inoculation site (local) and non-inoculation site (systemic) adverse events within 0-14 days, and unsolicited adverse events within 0 to 28 days.<br>➢ **Long-term safety observation**<br>All serious adverse events (SAEs), adverse events of special interest (AESIs), and pregnancy-related events within 6 months after vaccination will be collected from all participants.<br><br>**Immunogenicity observation (for immunogenicity subgroup only)**<br>For participants in the immunogenicity subgroup (100 cases), venous blood samples of approximately 15 mL will be collected before booster vaccination, 7 days, 14 days, 28 days, 3 months, and 6 months after booster (a total of 6 times). Separated serum will be detected for the virus-neutralizing antibody against the current main epidemic strain and specific S-protein IgG antibody.<br><br>Extra venous blood samples of the participants in the immunogenicity subgroup (100 cases) will be collected before vaccination, 7 days, 14 days, 28 days, and 3 months after booster (5 times in total) to detect cytokines of IL-2, IL-4, IL-13, and IFN-γ (ELISpot).
**Follow-up plan**	Within 28 days after the booster vaccination, all participants need to conduct at least 3 on-site visits to complete enrollment (visit 1), vaccination (visit 1), distribution, and collection of diary cards and contact cards (visit 1, visit 3, visit 4). The participants in the immunogenicity subgroup also need to collect blood for immunogenicity (visit 1-6).<br>From the 7th day to the 180th day after the booster vaccination, all participants need

	to conduct remote visits at least 22 times (about 8 times per month from the 7th day to the 90th day after the vaccination; about once per month from the 90th day to the 180th day after the vaccination), to carry out routine monitoring of VE and/or case monitoring after booster vaccination. At the same time, AEs, SAEs, AESIs, and pregnancy events (including pregnancy outcomes, delivery characteristics, the condition of the newborn, and the growth and development within 1 month after birth) need to be collected.<br>In addition to the on-site visits and remote visits mentioned above, the investigators may increase the on-site visits or remote visits to the participants to an appropriate frequency according to actual needs, and make follow-up records.
**Data collection**	The necessary data for statistical analysis will be collected using an electronic data capture (EDC) system.
**Inclusion criteria**	Participants must meet all the following inclusion criteria:<br>(1)Both male and female adults aged 18 years and above who can provide their identification;<br>(2)Understand the contents of the ICF and the vaccine for this vaccination, sign the ICF, and have the ability to use a thermometer, scale, and fill in the diary card and contact card as required;<br>(3)Be able to communicate well with the researcher and understand and comply with the requirements of this study;<br>(4)Participants must have received 2 or 3 doses of SARS-CoV-2 inactivated vaccine, with the last dose received at least 6 months before enrolment.<br>(5)Healthy participants or participants with mild underlying disease [in a stable state without exacerbation (no admission to hospital or no major adjustment to treatment regimen, etc.) for at least 3 months before enrollment in this study].<br>(6)For female participants: without childbearing potential (amenorrhea for at least 1 year or documented surgical sterilization) or not in pregnancy or lactation and have

	used effective contraception (e.g., intrauterine or implantable contraceptive devices, oral contraceptives, injectable or buried contraceptives, sustained-release topical contraceptives, intrauterine devices (IUDs), condoms (for males), diaphragms, cervical caps, etc.) for the past 14 days before the first dose of vaccine (provide negative pregnancy certificate within 48 hours);<br>(7)Negative results within 48 hours for SARS-CoV-2 pathogenicity testing (RT-PCR).
**Exclusion criteria**	Participants meeting any of the following criteria will be excluded from the study.<br>(1)Abnormal vital signs that are clinically significant (e.g., blood pressure that is still abnormal after control);<br>(2)COVID-19 infection in the last 6 months or use of any COVID-19 prophylactic medication other than 3 doses of inactivated SARS-CoV-2 inactivated vaccine (e.g., a history of vaccination with any other non-inactivated SARS-CoV-2 inactivated vaccine, marketed or unlisted, or vaccination with 1 or 4 doses of inactivated SARS-CoV-2 vaccine)<br>(3)A history of Severe Acute Respiratory Syndrome (SARS), Middle East Respiratory Syndrome (MERS), or other coronavirus infections at any time.<br>(4)Axillary temperature ≥37.3°C detected on the day of vaccination or fever within the last 24 hours (axillary temperature ≥37.3°C/oral temperature ≥37.5°C);<br>(5)A history of severe allergic or allergic reactions to vaccines or drugs, e.g., urticaria, severe skin eczema, dyspnea, laryngeal edema, angioneurotic edema, etc.<br>(6)Any other licensed vaccines given within 28 days before study vaccination;<br>(7)Participation in a clinical study of other drugs within 28 days before vaccination or planned participation within 6 months after vaccination;<br>(8)Have an inherited bleeding tendency or coagulation disorder (eg: Cytokine deficiency, coagulopathy, or thrombocytopenia), or a history of severe bleeding;

	(9)Diseases affecting the functioning of the immune system (e.g., cancer, except basal cell carcinoma of the skin), congenital or acquired immunodeficiencies (e.g., HIV infection), uncontrolled autoimmune diseases, etc., based on a known medical history or diagnosis;<br>(10)Asplenia or functional asplenia;<br>(11)Long-term use (continuous use for ≥14 days) of immunosuppressants or other immunomodulatory drugs (eg, corticosteroids: prednisone or equivalent; interferon, etc.) within 6 months before study vaccination. Topical medications (e.g., ointments, eye drops, inhalers, or nasal sprays) are permitted and should not exceed the dose recommended in the instructions;<br>(12)Receipt of immunoglobulins and/or blood products within 3 months before study vaccination;<br>(13)Suspected or known alcohol dependence or drug abuse;<br>(14)Participants deemed unsuitable for participation in this study based on the investigator's assessment.
**The criteria for early termination of the study**	(1)The participant has a disability, life-threatening adverse events, or serious adverse events, and needs to withdraw from the study in advance due to other reasons such as the need for treatment;<br>(2)The participant's health condition is at risk for safety and does not allow him to continue to participate in this study;<br>(3)Female participants are pregnant (if the vaccination has been completed, the participant does not need to be withdrawn from the study, and follow-up observation is required);<br>(4)The participant voluntarily requests to withdraw from the clinical study;<br>(5)The investigator considers the subject unsuitable for further participation in the clinical study.

**Study hypothesis**	The lower limit of the confidence interval for the protection efficacy (vaccine efficacy, VE) against symptomatic COVID-19 cases occurring from 14 days after booster vaccination in adults aged 18 and older is more than 30%.<br>Null hypothesis(H0): the lower limit of 95% confidence interval for the VE ≤30%<br>Alternative hypothesis(H1): the lower limit of 95% confidence interval for the VE ＞30%<br>The overall test level of the study is α=0.025(one-sided).
**Sample size estimation**	This study is a randomized, blinded, placebo-controlled clinical study. The sample size for this study is estimated as follows:<br>Sample size of protective efficacy: Assume that the incidence rate of COVID-19 in the study area will be approximately 9% and the expected VE of the vaccine will be at least 60%. The superiority margin of VE is 30%, the allocation ratio of the experimental group to the placebo control group is 1:1, the test level is 0.025 on one side, and the power of the test is 90%. The sample size is calculated using the exact conditional method under the large-sample Poisson distribution based on Chan and Bohidar. At least 162 confirmed cases of COVID-19 with clinical symptoms should be collected in the study, and a sample size of 1,286 participants is required for each group. Considering about 20% non-evaluable rate, 1,600 participants are planned to be enrolled in each group, with a total of 3,200 participants.<br>Due to the unpredictability of the number of cases in some areas and the change in the incidence rate in different regions, the number of COVID-19 cases will be monitored blindly during the research. The number of participants planned to be enrolled can be increased during the study according to the changes of the incidence in different regions, but the required number of clinically symptomatic COVID-19 cases of any severity cannot be changed, thus not causing inflation of the overall Type I error of the study.
**Relevant**	1. 18 years of age: 18 years of age (i.e., the day of the 18th birthday), but not yet 19

**definitions**	years of age (i.e., the day before the 19th birthday). Calculation of age: Each year of age is incremented by one year on the day of the subject's calendar birthday. For example, a subject born on January 01, 2002, will be 20 years old on January 01, 2022, and so on.<br><br>2. "X days apart" is defined as a period of x days between two dates, e.g., 3 days between January 1 and January 5, 3 days between day 0 and day 4, etc. 3.<br><br>3. "Month" is defined as "30" days. 4.<br><br>4. Seropositive conversion rate (SCR) is defined as pre-immunization neutralizing antibody <lower limit of detection, post-immunization neutralizing antibody ≥4 times lower limit of detection; pre-immunization neutralizing antibody >lower limit of detection, post-immunization neutralizing antibody ≥4 times higher than pre-immunization.<br><br>Person-year incidence rate = (number of cases/number of person-years of exposure of the subject) × 100%.

Table 1 Schedule of Activities (SOA)

On-site Visit[#]		Vaccination until 6 months after vaccination					
		V1	V2	V3	V4	V5	V6
		D0	D7	D14	D28	D90	D180
Time Window		/	+2	+7	+7	+15	+30
1	Screening, Informed consent, signing of ICF, allocation of screening number	X	/	/	/	/	/
2	Demographic data collection	X	/	/	/	/	/
3	Height, weight, respiration, general condition, head and neck, lymph nodes, skin, etc.	X	/	/	/	/	/
4	Pulse and blood pressure measurement	X	/	/	/	/	/
	Axillary temperature	X	/	/	/	/	/
5	Inclusion and exclusion criteria*	X	/	/	/	/	/
6	Enrollment, assigning randomization number	X	/	/	/	/	/
7	Immunogenicity tests (immunization subgroups only)[a]	X	X	X	X	X	X
8	Cellular immunity assays (immunization subgroup only)[b]	X	X	X	X	X	/
9	Vaccination	X	/	/	/	/	/
10	Observation for 30 minutes after vaccination, train participants to perform safety observations and recordings	X	/	/	/	/	/
11	Dispensation of diary cards	X	/	/	/	/	/
12	Distribution of soft measuring tape and thermometers to participants	X	/	/	/	/	/
13	Review and collect diary card	/	/	X	/	/	/

On-site Visit#		Vaccination until 6 months after vaccination					
		V1	V2	V3	V4	V5	V6
		D0	D7	D14	D28	D90	D180
Time Window		/	+2	+7	+7	+15	+30
14	Distribution of contact cards	/	/	X	/	/	/
15	Review and Recall Contact Cards	/	/	/	X	/	/
16	Adverse events	X	X	X	X	/	/
17	Concomitant medication	X	X	X	X	/	/
18	Protective Effectiveness Visits<br>Routine and case monitoring	X	X	X	X	X	X
19	SAE、AESI	X	X	X	X	X	X
20	Pregnancy-related events	X	X	X	X	X	X
21	EOS&	/					X

*: The participant's vaccination record information for the COVID-19 vaccine was confirmed by checking the health code, etc.

#: When the on-site visit coincides with the required telephone visit, no additional telephone visit will be conducted.

a: Immunogenic blood collection for immunized subgroup participants only.

b: Additional cellular immunity blood collection for immunized subgroup participants only.

&: EOS form: all participants should complete the EOS form at the V6 visit and subjects will be contacted via EOS phone call to obtain the most safety data in the event the subject decides to terminate early.

Research Team

**Protocol Number**	LVRNA012-IIT-04
**Version Number**	2.1
**Version Date**	February 8th, 2023
**Study Title**	A Single-Center, Randomized, Blinded, Placebo-Controlled Clinical Study to Evaluate the Efficacy, Safety, and Immunogenicity of a SARS-CoV-2 Variant (Omicron BA.5) mRNA Vaccine (LVRNA012) Vaccine Administered as 1 Dose in adults Aged 18 Years and Older Who Have Completed 2 or 3 Doses of SARS-CoV-2 Inactivated Vaccine.
**Institutions responsible for clinical research**	
Unit: The first affiliated hospital of Bengbu Medical College<br>Address: No. 287, Changhuai Road, Bengbu, Anhui Province, China<br>Principal Investigator: Huan Zhou、Qiang Wu<br>Telephones: 13665527160 18055207028<br>E-mail: zhouhuanbest@163.com	
**Funder**	
Funder 1: AIM Vaccine Co. Ltd.<br>Address: Room 218, 2nd Floor, Xinghai Building, No. 16 Yingshun Road, Yinghai Town, Daxing District, Beijing<br>Funder 2: Ningbo Rong'an Biopharmaceutical Co., Ltd<br>Address: No. 21 Chuangye Avenue, Ningbo Free Trade Zone, Zhejiang Province<br>Sponsor Contact: Fan Zhang<br>Telephones: 13671828804	

E-mail: fan.zhang@aimbio.com
**CRO**
Unit: Beijing Kekuen Technology Development Co., Ltd<br>Contacts: Lu Jiao<br>Telephones: 18952675300
**Data Management and Statistical Analysis Unit**
Unit: Beijing Kekuen Technology Development Co., Ltd<br>Contacts: Chao Gao<br>Telephones: 17612408686

1. Study Title

A Single-Center, Randomized, Blinded, Placebo-Controlled Clinical Study to Evaluate the Efficacy, Safety, and Immunogenicity of a SARS-CoV-2 Variant (Omicron BA.5) mRNA Vaccine (LVRNA012) Vaccine Administered as 1 Dose in adults Aged 18 Years and Older Who Have Completed 2 or 3 Doses of SARS-CoV-2 inactivated Vaccine.

2. Introduction

Coronavirus Disease 2019 (COVID-19) has continued to rage around the world since its outbreak in December 2019; the World Health Organization (WHO) declared COVID-19 as a Public Health Emergency of International Concern (PHEIC) on January 30, 2020, and upgraded it to a global pandemic on March 11, 2020. WHO data show that as of December 15, 2022, a total of 224 countries or regions around the world have reported a cumulative total of more than 646 million confirmed cases and 6.63 million deaths. The outbreak and epidemics of COVID-19 have put heavy economic pressure and medical burdens on people worldwide and pose a serious threat to the survival and health of human beings.

Vaccination is the best intervention to prevent COVID-19, as it reduces all types of complications and disease burden, and interrupts the transmission of the virus. Currently, more than 374 vaccine candidates against SARS-CoV-2 are under development worldwide (about 175 are in a clinical study) including various vector vaccines (replicative or non-replicative), nucleic acid-based vaccines (mRNA and DNA), inactivated vaccines, recombinant subunit vaccines, and live attenuated vaccines. Some of these vaccines have been conditionally approved or emergency use in different countries or WHO. However, China's independently developed COVID-19 mRNA vaccine has not yet been approved as of now.

Further booster vaccinations for vaccinated populations have brought widespread global attention as the emergence of SARS-CoV-2 variants and the decline in antibody levels over time after vaccination. According to a joint release from Pfizer and BioNTech on July 09, 2022, their ongoing trial of the third dose of booster vaccination with a SARS-CoV-2 mRNA vaccine showed positive results. The results showed that subjects who received a third dose of booster vaccine six months after receiving two doses of the COVID-19 mRNA vaccine (BNT162b2) showed high neutralizing titers in sera against the wild-type strain and Beta variant strain, which is 5- to 10-fold increases in neutralizing antibody titers compared to subjects who received only two doses of the vaccine, also showed prior consistent tolerability profile and safety profile. Sequential vaccination using different technology pathways is expected to increase the strength, breadth, and durability of the immune response through complementarity between different mechanisms, resulting in more comprehensive and potent protection. A study in Turkey showed that booster immunization with mRNA vaccine after completion of 2 doses of inactivated vaccine induced 27 times higher levels of IgG antibodies compared with booster immunization with the 3rd dose of inactivated vaccine homologue.

Because the Omicron variant is highly transmissible, has a greater ability to evade immunization despite its reduced pathogenicity. That resulted in most of the currently marketed vaccines developed by the original strain having low protective efficacy against the disease caused by this variant. The development of new vaccines against the variant, as well as new vaccination protocols, has become particularly important.

Since the outbreak of COVID-19, the CPC Central Committee and the State Council have optimized the prevention and control measures according to the characteristics of the virus mutation and the development of the epidemic. Recently, they have optimized and put forward "20" and "10" targeted measures to continuously improve the level of scientific precision in prevention and control, maximize the protection against the disease, minimize the impact of the epidemic on people's production and

living order and economic and social development. With the gradual liberalization of epidemic prevention and control as well as the acceleration of economic construction, the movement of people, and the increase of social activities, China facing greater pressure in epidemic prevention and control work especially in the medical resources. Therefore, in the time after three doses of the new crown vaccine to strengthen the vaccination of the fourth dose has become a necessary measure for the control of the new crown epidemic. Meanwhile, based on the vaccination status of the marketed SARS-CoV-2 vaccine in China and the consensus on the advantages of sequential booster immunization in both domestic and international studies, it is of great significance to carry out a clinical study on sequential booster immunization in the population that has already completed 2 or 3 doses of inactivated SARS-CoV-2 vaccine.

This study is a clinical research of a SARS-CoV-2 variant mRNA vaccine (LVRNA012). The study was single-center, randomized, blinded, and placebo-controlled. The protective efficacy, safety, and immunogenicity of the study vaccine were evaluated with 1 dose of the mRNA vaccine (LVRNA021) as a booster in participants aged 18 years and older who had completed 2 or 3 doses of the vaccine.

3. Background and rationale

3.1. Pathogenetic Background

The novel coronavirus (SARS-CoV-2) belongs to the β genus of coronaviruses with an envelope and round or oval viral particles, 60-140nm in diameter. The virus particles contain four structural proteins: spiking protein (spike, S), envelope protein (envelope, E), membrane protein (membrane, M), and nucleocapsid protein (nucleocapsid (N). Nucleocapsid protein N wrapped around the viral RNA to form the virus particle core structure- nucleocapsid, nucleocapsid then wrapped by the

bilayer lipid membrane, bilayer lipid membrane embedded with the SARS-CoV-2 S, M, N protein. After invading the human respiratory tract, neocoronaviruses rely on the receptor binding domain (RBD) on the S protein on their surface to recognize the host cell receptor angiotensin-converting enzyme 2 (ACE2) and bind to it to infect the host cell.

SARS-CoV-2 is sensitive to ultraviolet light, organic solvents (ether, 75% ethanol, peroxyacetic acid and chloroform, etc.), and chlorine disinfectants. 75% ethanol and chlorine disinfectants are commonly used for clinical and laboratory inactivation of neocoronaviruses, but chlorhexidine is not effective in inactivating the virus.

3.2. Disease Background

3.2.1. Epidemiology

COVID-19 has been included as a Category B infectious disease under the Law of the People's Republic of China on Prevention and Control of Infectious Diseases, and preventive and control measures for Category B infectious diseases have been taken.

According to the Diagnostic and Treatment Program for COVID-19 (Trial 10th Edition), COVID-19 has the following transmission characteristics.

Source of infection: The source of infection is mainly the SARS-CoV-2 infected people, in the incubation period is infectious, with in 3 days of onset is the most contagious.

Transmission: 1. Transmission through respiratory droplets and close contact is the main means of transmission. 2. Transmission through aerosols in relatively closed environments. 3. Contact with objects contaminated by the virus can also cause infection.

Susceptible population: The population is generally susceptible. A certain degree of immunity can be obtained after infection or vaccination against the new coronavirus. The elderly and patients with serious underlying medical conditions have higher rates of severe illness and death after infection than the general population, and vaccination can reduce the risk of severe illness and death.

Variation: Like other viruses, the genome of SARS-CoV-2 mutates. During epidemics and transmission in the population, mutations occur in the genes of SARS-CoV-2 frequently. When different subtypes or daughter branches of SARS-CoV-2 infect humans at the same time will result in recombinant strains of the virus. Some of these mutations or recombinations affect the biological properties of the virus. As of the end of 2022, the World Health Organization (WHO) has proposed five variant of concern (VOC) strains: Alpha (B.1.1.7), Beta (B.1.351), Gamma (P.1), Delphi (P.1), and P.1 (P.1). P.1), Delta (Delta, B.1.617.2), and Omicron (Omicron, B.1.1.529). The Omicron variant appeared in the population in November 2021, with significantly enhanced transmission and immune escape compared to other VOC variants such as Delta, rapidly replacing the Delta variant in early 2022 as the absolute dominant prevalent strain globally.

To date, the five subtypes of omicron (BA.1, BA.2, BA.3, BA.4, and BA.5) have evolved into a series of 709 daughter sub-branches, including 72 recombinant branches. New omicron sub-branches will continue to emerge as the SARS-CoV-2 continues to spread globally. BA.5.2 has been the predominant omicron variant globally for several months, but since October 2022, sub-branches BF.7, BQ.1, and BQ.1.1 and recombinant variants (XBB), which are more immune evasive and transmissible, have rapidly increased in predominance, replacing BA.5.2 as the predominant prevalent strain in some countries and regions.

Domestic and international evidence shows that the pulmonary pathogenicity of the Omicron variant has been significantly reduced, and the clinical manifestations have changed from pneumonia to upper respiratory tract infections. The diagnostic

accuracy of the PCR test routinely used in China has not been affected, but the neutralizing effect of some monoclonal antibody drugs that have been developed and marketed has been significantly reduced.

3.2.2. Clinical Manifestation

The incubation period is 2-4 days.

The main manifestations are dry throat, sore throat, cough, fever, etc. The fever is mostly low to medium, and in some cases, it may be high, and the duration of fever is not more than 3 days; some patients may have muscle pain, loss of smell and taste, nasal congestion, runny nose, diarrhea, conjunctivitis, etc. A few patients continue to develop the disease and develop symptoms related to pneumonia. In a few patients, the disease continues to develop, the fever persists, and pneumonia-related manifestations appear. Severe patients tend to develop respiratory distress and/or hypoxemia 5 to 7 days after the onset of illness. In severe cases, the disease may progress rapidly to acute respiratory distress syndrome, septic shock, uncorrectable metabolic acidosis and hemorrhagic coagulopathy, and multi-organ failure. Very few patients may also have central nervous system involvement and other manifestations. The clinical manifestations of infection in children are similar to those of adults, and high fever is relatively common. Most patients have a good prognosis, and those in critical condition are mostly seen in the elderly, those with chronic underlying diseases, late pregnancy and perinatal women, and obese people.

3.2.3. Morbidity levels and vaccine use

WHO data show that as of December 15, 2022, a total of 224 countries or regions around the world have cumulatively reported more than 646 million confirmed cases and more than 6.63 million deaths; the cumulative number of SARS-CoV-2 vaccines reported to have been administered is about 12,814,704,622 doses. Official data from the National Health Commission shows that as of December 18th, China has accumulated 8,578,991 confirmed cases of SARS-CoV-2 infection and 27,666

deaths; the cumulative number of vaccines reported to have been administered is about 3,457,953,632 doses.

3.3. Vaccine Brief

Current nucleic acid vaccines include DNA vaccines and messenger RNA (mRNA) vaccines. DNA vaccines use genetic engineering to clone the gene coding for a novel coronavirus protein into an expression vector, and then transfer this recombinant plasmid into the organism, so that the gene coding for the antigenic protein can be expressed with the help of transcription and translation in the host, and then present the antigen to the immune cells through the antigen-presenting cells. The antigen is then presented to the immune cells through the antigen-presenting cells to activate the specific cellular and humoral immune response of the organism.

An mRNA vaccine utilizes host (vaccinator) cells to express viral proteins from mRNA injected into the body and then induce antibody production. Compared to traditional technology platforms such as inactivated and attenuated vaccines, which have long vaccine development cycles, difficult screening of pathogenic strains, and expensive consumables for the required equipment and other constraints, mRNA vaccines show great advantages in the field of newly discovered viral vaccine research and development because they do not require pathogenic strains, do not introduce mutations, can be degraded by normal cells, have a high efficiency of expression, and have a short production cycle.

Usually, nucleic acid molecules cannot freely pass through biological membranes and are easily degraded by ribonuclease (RNase) in plasma and tissues, rapidly cleared by the liver and kidney, and recognized by the immune system, which impedes their smooth entry into the body to play a role. To ensure the metabolic stability and immune activity of mRNA vaccines, the delivery method and formulation design of mRNA are the key to vaccine development. The mRNA vaccine delivery vectors

reported so far mainly include viral vectors and non-viral vectors. Although viral vectors, mainly lentivirus, adeno-associated virus, Sendai virus, etc., are capable of delivering nucleic acids, the viral vectors themselves may induce immune responses or cause infections, thus limiting the application of vaccines. Non-viral vectors mainly include liposomes, dendritic cells, inorganic nanoparticles, and cationic cell-penetrating peptides. Among them, lipid nanoparticles have unique advantages as the most effective carriers to deliver mRNA: 1) lipid nanoparticles are spherical vesicles that can encapsulate mRNA and resist the action of nuclease; 2) lipid nanoparticles usually contain ionizable cationic lipids with a pK of around 6.5, which are positively charged under the acidic pH conditions of endosomes (around 5) and thus interact with the negatively charged ectodomain and trigger cell fusion, thus the transfection efficiency is high; 3) lipid nanoparticles can deliver mRNAs of different sized fragments; 4) lipid nanoparticles are not restricted by the host as a delivery vehicle.

From the development principle and action mechanism, mRNA vaccines have advantages in safety, efficacy, and production convenience. In terms of safety, since mRNA does not infect or integrate into the genome, mRNA vaccination does not pose a risk of infection or mutation. In terms of efficacy, various modification methods can make mRNA more stable and more efficient in translation to ensure accurate expression of target antigenic proteins; Also the development of delivery methods can enable mRNA to be rapidly delivered to the cell to perform its function. In terms of production convenience, current in vitro transcription technology can produce mRNA vaccines on a large scale in a very fast and inexpensive manner. Compared with the production cycle of traditional vaccines, which takes 5-6 months, it is expected that mRNA vaccines can complete the production of vaccine samples in a shorter time, which is more suitable for coping with the rapidly growing and widely spreading epidemics at present. Up to now, two mRNA vaccines developed by Pfizer/BioNTech and Moderna have been approved overseas. in addition, a SARS-CoV-2 mRNA

developed by Watson Biologicals based on the receptor-binding region (RBD) design of the prototype strain has been granted emergency use authorization in Indonesia.

4. Background of the SARS-CoV-2 variant (Omicron BA.5) mRNA vaccine (LVRNA012) study

4.1. Theoretical foundations

The funders conducted extensive and in-depth research on the DNA template construction, in vitro enzyme transcription synthesis, purification, and other processes of the mRNA vaccine, conducted systematic comparative research on the quality control of the vaccine, established the production process, and carried out large-scale trial production in a GMP-compliant workshop. The vaccine has passed the tests of sterility test, identification test, and potency test. Self-inspection was done before the clinical trial was formally carried out, and all the indexes of the product meet the quality standard requirements in the "Manufacturing and Testing Regulations for SARS-CoV-2 mRNA Vaccine (Declaration)".

LVRNA012 is a SARS-CoV-2 mRNA vaccine constructed and produced with linearized vector by genetic recombination technology, mRNA molecules are synthesized by in vitro enzyme catalytic technology, purified, and obtained by preparation process. The funders have conducted systematic research on the process and quality control of the mRNA vaccine and established the production process and quality standards. The results of non-clinical studies, such as acute toxicity, long-term toxicity, allergy tests, and vaccine efficacy studies in animals, show that the product is safe and effective.

According to current research, among the four known structural proteins of coronaviruses, the spiking protein is the one that induces a protective immune response. Some scholars have confirmed that the C-terminal end of the spiking

protein of coronaviruses is the antigenic determinant of coronaviruses. At the same time, SARS-associated coronaviruses are infected through the respiratory tract, and there is currently no vaccine to prevent SARS. There is no vaccine against SARS.

This product is an adjuvant-free mRNA vaccine made by producing a messenger RNA (mRNA) molecule that can be translated into a SARS-CoV-2 spiking protein (S protein) through the mRNA technology platform and encapsulated by nanoparticles. The mechanism of action is that the mRNA molecules synthesized in vitro and coding for the specific antigenic proteins of SARS-CoV-2 are delivered into the cells, making the cells become factories for the production of antigenic proteins and triggering an immune response, which in turn serves as a vaccine for the protection of the vaccine.

LVRNA021 is a PBS aqueous end product consisting of mRNA molecule API combined with lipid excipients to form lipid nanoparticles. mRNA molecule is 4072 nucleotide sequence length and its theoretical molecular weight is 1321 kDa. The product components include mRNA molecule encoding the full length of the SARS-CoV-2 mutant Omicron BA.5 echinocandin S protein, Dlin-MC3-DMA, cholesterol, distearoyl phosphatidylcholine, PEG2000-DMG, phosphate buffer.

4.2. Risk Assessment

4.2.1. Risk of injecting study vaccines

As with any vaccination, there is a potential risk of anaphylactic reaction to the vaccine administered in this study. Following vaccination, all subjects will be asked to remain in the hospital for at least 30 minutes to identify and address potential safety risks.

The potential risks to subjects during the clinical study of this product are mainly due to the common adverse events of vaccine injection, which are localized adverse events: pain, hardness, erythema, rash, swelling, itching, and cellulitis at the site of

vaccination; and systemic adverse events: fever (axillary warmth), diarrhea, nausea, vomiting, headache, myalgia (not at the site of vaccination), arthralgia, chills, anorexia, fatigue/weakness, and acute Allergic reaction. Generally resolves on its own without treatment. A few reactions are strong and will be closely monitored by the investigator and treated symptomatically.

4.2.2. Antibody-mediated (infection) potentiation or vaccine-enhanced disease

In some cases, antibodies may play a role in enhancing viral infection during viral infection by assisting viral entry into target cells and increasing the rate of infection, that phenomenon known as Antibody-Dependent Enhancement (ADE) or Vaccine Enhanced Disease (VED). Fc fragment-mediated ADE increases viral invasion. Both virus-antibody immune complexes and Th2 immune-biased responses can occur in cases of VED. Such phenomena have been observed in preclinical animal testing of a SARS vaccine (rhesus monkeys), and a vaccine that prevents respiratory syncytial virus-infected diseases in children.

To date, no ADEs and/or VEDs have been observed in preclinical or clinical studies with the study vaccine. All subjects, especially suspected or confirmed cases of COVID-19, will still be closely monitored and followed throughout the study to ensure subject safety. If a subject reports a suspected or confirmed case during the study, he/she will be required to go to the study-designated research center or hospital for hospitalization.

4.2.3. Other potential risks

According to the Safety Platform for Emergency vACcines (SPEAC) public information, several rare diseases are associated with the COVID-19 vaccine, including neurological disorders, vascular disorders, and cardiac disorders. These diseases can be categorized as adverse events of special interest (AESI).

Neurologic, vascular, and cardiac risks will be closely monitored in this study. Based on the data from the pre-study vaccine trial, no evidence of the above risks of special

concern was observed in the subjects. Subjects will be closely monitored throughout this study from the first dose to the end of the study to ensure subject safety.

4.2.4. Risk of blood collection

Venipuncture is a routine clinical procedure for obtaining blood samples. Venepuncture may cause mild pain. Skin/soft tissue infections at the puncture site or in the vein are very rare. The total volume of blood collected in this study will not be detrimental to the health of the subjects.

4.2.5. Risk of disclosure of personal medical information

The personal information and privacy of all subjects will be kept strictly confidential with the following safeguards: 1) access to study documents and personal information will be limited to investigators, clinical medical research ethics committees, regulatory agencies, and funders; 2) study information materials will be kept in a locked room; and 3) all information or samples delivered to the outside of the study site will be labeled with a unique study identifier number, and personally identifiable information will be declassified.

4.3. Benefits assessment

Subjects' personal information will be kept confidential.

l All subjects will be examined (including but not limited to routine physical examination, vital signs, etc.) and the results will be provided free of charge. Adverse events following vaccination will be closely monitored by the investigator throughout the study. Appropriate and adequate medical treatment will be provided to subjects if needed.

l Subjects may not directly benefit from receiving the study vaccine, but by participating in this study, they can contribute to the marketing of mRNA vaccines,

which will help more people prevent diseases associated with SARS-CoV-2 infections.

4.4. Benefit/risk assessment

Because the preventive effect of the study vaccine against disease caused by SARS-CoV-2 infection, same as any other study vaccine, may not be 100%. Because it is possible that subjects may enter the placebo group and may not develop protection against SARS-CoV-2 infection after vaccination, there remains a risk of morbidity from natural infection with SARS-CoV-2.

Based on the following considerations, participation in this study subjects exposed to a low risk of developing unknown adverse reactions.

1)According to the animal toxicity test of this product suggests that the safety risks associated with it can be minimized during clinical studies by following the vaccination dose and vaccination protocol specified in the study protocol and by close monitoring.

2) The clinical study protocol establishes strict criteria for termination/suspension of the study.

3) During the study, the investigator will focus on the safety evaluation and the solicited local adverse events, solicited systemic adverse events, and other adverse events after vaccination. Risk control measures, including appropriate enrollment and exclusion criteria, close monitoring of subjects, and suspension/early termination criteria, will be established before the study begins to ensure maximum benefit to the subjects.

Based on these safeguards, the potential risks associated with the study vaccine are reasonable concerning the benefits that could be realized for the prevention of COVID-19 in subjects.

4.5. Summary

In summary, this product demonstrated good safety and immunogenicity in non-clinical animal studies. With the continuous discovery of SARS-CoV-2 mutant strains, the effectiveness of the existing marketed vaccines against the mutant strains has declined. Moreover, as the vaccination rate of SARS-CoV-2 vaccines has increased in various countries, domestic and foreign experts have gradually begun to explore the impact of booster vaccination with multiple technical routes of SARS-CoV-2 vaccines on immunogenicity. Considering the relatively good safety of the inactivated vaccine and the decline in neutralizing antibodies over time, the sponsor plans to administer a booster dose of the SARS-CoV-2 variant (Omicron BA.5) mRNA vaccine (LVRNA012) or a placebo to those who have already received 2 or 3 doses of the novel inactivated coronavirus vaccine to assess the effect of the SARS-CoV-2 variant (Omicron BA.5 mRNA vaccine (LVRNA012), and also to observe the immunogenicity of some of the subjects, to provide a confirmatory basis for the efficacy and safety of the sequential booster vaccination with the SARS-CoV-2 mRNA vaccine.

5. Participants

The participants of this study are adults aged 18 and older who have been inoculated with 2 or 3 doses of inactivated COVID-19 vaccines ≥6 months.

6. Study Design

6.1. Study objective(s)

6.1.1. Primary objective(s)

- **The primary objective of protective efficacy**

1)To evaluate the protective efficacy of the investigational vaccine or placebo in the prevention of symptomatic COVID-19 cases of any severity (Appendix 1) occurring from 14 days after booster vaccination in adults aged 18 and older who have completed 2 or 3 doses of inactivated COVID-19 vaccines.

6.1.2. Secondary objective(s)

- Secondary objective(s) of protective efficacy

1)To observe the protective efficacy of the investigational vaccine or placebo in the prevention of severe and critical COVID-19 cases (Appendix 1) from 14 days after booster vaccination in adults aged 18 and older who have completed 2 or 3 doses of inactivated COVID-19 vaccines.

2)To observe the protective efficacy of the investigational vaccine or placebo in the prevention of COVID-19 cases leading to death (Appendix 1) from 14 days after booster vaccination in adults aged 18 and older who have completed 2 or 3 doses of inactivated COVID-19 vaccines.

- Secondary objective(s) of safety

1)To evaluate the safety of the investigational vaccine or placebo within 28 days after booster vaccination in adults aged 18 and older who have completed 2 or 3 doses of inactivated COVID-19 vaccines.

2)To observe the safety of the investigational vaccine or placebo within 6 months after booster vaccination in adults aged 18 and older who have completed 2 or 3 doses of inactivated COVID-19 vaccines.

- Secondary objective(s) of immunogenicity

1)To evaluate the SARS-CoV-2 virus-neutralizing antibody against the main epidemic strains among the immunogenicity subgroup 7 days, 14 days, 28 days, 3 months, and 6 months after booster vaccination of the investigational vaccine or placebo.

2)To evaluate the SARS-CoV-2 S-protein IgG antibody against the main epidemic strains among the immunogenicity subgroup 7 days, 14 days, 28 days, 3 months, and 6 months after booster vaccination of the investigational vaccine or placebo.

6.1.3. Exploratory objective(s)

- Exploratory objective(s) of protective efficacy

1)To observe the protective efficacy of the investigational vaccine or placebo in the prevention of symptomatic COVID-19 cases of any severity (Appendix 1) occurring from 7 days after booster vaccination in adults aged 18 and older who have completed 2 or 3 doses of inactivated COVID-19 vaccines.

- Exploratory objective(s) of immunogenicity

1)To observe the specific cellular immune response among the immunogenicity subgroup 7 days, 14 days, 28 days, and 3 months after booster vaccination of the investigational vaccine or placebo in the adults aged 18 and older who have completed 2 or 3 doses of inactivated COVID-19 vaccines.

To explore the relationship between neutralizing antibody, S-protein IgG antibody, and/or cellular immunity and vaccine protective efficacy after vaccination.

6.2. Study endpoint(s)

6.2.1. Primary endpoint(s)

- The primary endpoint(s) of protective efficacy

1)The person-year incidence rate of symptomatic COVID-19 cases of any severity (Appendix 1) occurring from 14 days after booster vaccination of the investigational

vaccine or placebo in adults aged 18 and older who have completed 2 or 3 doses of inactivated COVID-19 vaccines.

6.2.2. Secondary endpoint(s)

- Secondary endpoint(s) of protective efficacy

1)The person-year incidence rate of severe and critical COVID-19 cases (Appendix 1) occurring from 14 days after booster vaccination of the investigational vaccine or placebo in the adults aged 18 and older who have completed 2 or 3 doses of inactivated COVID-19 vaccines.

2)The person-year incidence rate of COVID-19 cases leading to death (Appendix 1) occurring from 14 days after booster vaccination of the investigational vaccine or placebo in adults aged 18 and older who have completed 2 or 3 doses of inactivated COVID-19 vaccines.

- Secondary endpoint(s) of immunogenicity

1)The geometric mean titer (GMT), geometric mean increase (GMI), and seroconversion rate (SCR) of SARS-CoV-2 virus neutralizing antibody against the main epidemic strains among the immunogenicity subgroup 7 days, 14 days, 28 days, 3 months and 6 months after booster vaccination of the investigational vaccine or placebo.

2)The geometric mean titer (GMT), geometric mean increase (GMI), and seroconversion rate (SCR) of SARS-CoV-2 S-protein IgG antibody against the main epidemic strains among the immunogenicity subgroup 7 days, 14 days, 28 days, 3 months and 6 months after booster vaccination of the investigational vaccine or placebo.

- Secondary endpoint(s) of safety

1)The incidence of AEs within 30 mins, 14 days, and 28 days after booster vaccination of the investigational vaccine or placebo in adults aged 18 and older who have completed 2 or 3 doses of inactivated COVID-19 vaccines.

2)The incidence of SAEs and AESIs within 6 months after booster vaccination of the investigational vaccine or placebo in adults aged 18 and older who have completed 2 or 3 doses of inactivated COVID-19 vaccines.

3)The pregnancy events (including the pregnancy outcome, the delivery characteristics, the condition of the newborn, and the growth and development within 1 month after birth) within 6 months after booster vaccination of the investigational vaccine or placebo in the adults aged 18 and older who have completed 2 or 3 doses of inactivated COVID-19 vaccines.

6.2.3. Exploratory endpoint(s)

➢ Exploratory endpoint(s) of protective efficacy

1)The person-year incidence rate of symptomatic COVID-19 cases of any severity (Appendix 1) occurring from 7 days after booster vaccination of the investigational vaccine or placebo in the adults aged 18 and older who have completed 2 or 3 doses of inactivated COVID-19 vaccines.

➢ Exploratory endpoint(s) of immunogenicity

1)The specific cellular immune response detected via cytokines IL-2, IL-4, IL-13, IFN-γ (ELISpot) among the immunogenicity subgroup 7 days, 14 days, 28 days, and 3 months after booster vaccination of the investigational vaccine or placebo.

2)The correlation coefficient between neutralizing antibody, S-protein IgG antibody, and/or cellular immunity and vaccine protective efficacy after vaccination.

6.3. Overall Design

A single-center, randomized, blinded, placebo-controlled design will be used for this study.

6.4. Study hypothesis

The lower limit of the confidence interval for the protection efficacy (vaccine efficacy, VE) against symptomatic COVID-19 cases occurring from 14 days after booster vaccination in adults aged 18 and older is more than 30%.

Null hypothesis(H0): the lower limit of 95% confidence interval for the VE ≤30%

Alternative hypothesis(H1): the lower limit of 95% confidence interval for the VE ＞ 30%

The overall test level of the study is α=0.025(one-sided).

6.5. Sample size and groups

6.5.1. Sample size estimation

This study is a randomized, blinded, placebo-controlled clinical study. The sample size for this study is estimated as follows:

Sample size of protective efficacy: Assume that the incidence rate of COVID-19 in the study area will be approximately 9% and the expected VE of the vaccine will be at least 60%. The superiority margin of VE is 30%, the allocation ratio of the experimental group to the placebo control group is 1:1, the test level is 0.025 on one side, and the power of the test is 90%. The sample size is calculated using the exact conditional method under the large-sample Poisson distribution based on Chan and Bohidar. At least 162 confirmed cases of COVID-19 with clinical symptoms should be collected in the study, and a sample size of 1,286 participants is required for each

group. Considering about 20% non-evaluable rate, 1,600 participants are planned to be enrolled in each group, with a total of 3,200 participants.

Due to the unpredictability of the number of cases in some areas and the change in the incidence rate in different regions, the number of COVID-19 cases will be monitored blindly during the research. The number of participants planned to be enrolled can be increased during the study according to the changes of the incidence in different regions, but the required number of clinically symptomatic COVID-19 cases of any severity cannot be changed, thus not causing inflation of the overall Type I error of the study.

6.5.2. Sample Size Allocation

This study plans to enroll approximately 3,200 participants who have completed 2 or 3 doses of inactivated COVID-19 vaccine for ≥ 6 months (Study No. 0001~3200). All participants with informed consent, physical examination, and screening will be 1:1 randomly assigned into the experimental group or the control group (1,600 participants in each group) and followed up for safety and protective efficacy.

In this study, the first 50 participants enrolled in each group (a total of 100 participants) will be set as the immunogenicity subgroup (Study No. 0001~0100). All participants enrolled need to be followed up for safety and protective efficacy after vaccination. The participants in the immunogenicity subgroup also need to collect blood for the detection of immunogenicity-related indicators before vaccination and 7 days, 14 days, 28 days, 3 months, and 6 months after vaccination. Extra blood will be collected for cellular immunity test before vaccination and at days 7, 14, 28, and month 3 after vaccination.

The groups, vaccines, and planned sample size are detailed in the table below:

Participants	Groups	Vaccines	Immunogenicity subgroup	Non-immunogenicity subgroup	Sample size(no.)
Adults aged 18 and older who have been	Study group	LVRNA012	50	1550	1,600
	Control group	Placebo	50	1550	1,600

inoculated with 2 or 3 doses of inactivated COVID-19 vaccines for ≥6 months	Total	100	3100	3,200

6.6. Observation of protective efficacy

7 days after the booster vaccination for every participant, the investigators need to carry out the following "routine monitoring" on the participants through a combination of remote visits and on-site visits:

- The investigators remotely visit the participants to see if they have symptoms related to COVID-19;
- When the investigator learns that the participant has any one or more of the following conditions through remote visits, it is necessary to collect the participant's throat swab sample A1 (for SARS-CoV-2 nucleic acid or antigen detection), and further inquiry, record, and guidance should be done to the participant to continue to observe and record symptom types, start and end time, severity, etc. If an on-site visit is conducted to the participant (the on-site visit could be carried out by the participant going to the study site or the investigators' visit), another nucleic acid throat swab sample B1 (backup sample) needs to be collected:

④ Any possible signs or symptoms related to COVID-19 appear(fever or chills, malaise/fatigue, headache, dry throat, muscle pain, sore throat, nasal congestion/runny nose, anorexia/nausea/vomiting, diarrhea, new sense of smell/ dysgeusia, conjunctivitis);

⑤ Any one or more respiratory-related symptoms (cough, shortness of breath, or dyspnea) appear and last for any time;

⑥ Clinical or imaging evidence of pneumonia.

If the result of SARS-CoV-2 nucleic acid or antigen detection of the participant's throat swab sample A1 is positive, the investigators need to

continue to carry out the following "case monitoring" on the participant:

- Send the participant's nucleic acid throat swab sample B1 (if collected) to the central laboratory for SARS-CoV-2 variant strain type detection, and guide the participant to go to the designated hospital for chest imaging examination (chest CT) ;
- Provide necessary medical supplies to the participant, and continue to remotely visit him/her twice a week. Instruct the participant to observe and record symptoms and self-medication at home, or guide him/her to go to the designated hospitals for medical treatment according to the situation. If the participant has symptoms such as shortness of breath or dyspnea, guide him/her to detect blood oxygen saturation according to the situation. According to the symptoms recovery of the participant, conduct an on-site visit to collect a throat swab sample or antigen nasopharyngeal swab sample for SARS-CoV-2 nucleic acid or antigen detection, until the result of SARS-CoV-2 nucleic acid or antigen turns negative or the symptoms disappear or stabilize under the investigators' evaluation.
- Collect the participant's treatment and medical history to judge the severity of COVID-19, and make the records about the type, starting time, and reporting time of the participant's symptom, and the collection time, detection time, and reporting time of each nucleic acid sample, and the diagnosis time and outcome of the case, to form relevant records;
- Establish a COVID-19 case file for the participant, and fill in and report the first/follow-up COVID-19 case report according to the remote visit;
- On-site monitoring and medical monitoring teams need to conduct a preliminary review of the completeness, rationality, and logic of the COVID-19 case report, and raise necessary inquiries and requirements of information supplementary, then submit them to the investigators for diagnosis and judgment.

If the result of SARS-CoV-2 nucleic acid or antigen detection in the participant's throat swab sample A1 is negative, the following retest should be continued:

- The participant's throat swab sample A2 (for SARS-CoV-2 nucleic acid or antigen detection) and sample B2 (backup sample) need to be collected again within 48 hours;
- If the result of SARS-CoV-2 nucleic acid or antigen detection of the participant's throat swab sample A2 is positive, the above "case monitoring" process should be followed to carry out relevant work;
- If the result of SARS-CoV-2 nucleic acid or antigen detection of the participant's throat swab sample A2 is still negative, the above "routine monitoring" process should be continued to carry out relevant work.

Frequency and methods of "visits":

- From the 7th day after the booster vaccination, remote visits will be conducted at a frequency of twice a week (2-3 days' intervals is recommended), until the 90th day after vaccination: (1) If the number of confirmed primary endpoint cases reaches 162, the frequency of visits will be adjusted to once a month, until the 180th day after vaccination; (2) If the number of confirmed primary endpoint cases does not reach 162, remote visits will still be conducted twice a week. When the number reaches 162, the visit frequency will be adjusted to once a month, until the 180th day after vaccination;
- If the participant has a positive result of SARS-CoV-2 nucleic acid or antigen detection, no matter what stage he/she is in, he/she will be visited twice a week (2-3 days' interval is recommended), until his/her nucleic acid or antigen detection result turns negative or the symptoms disappear or stabilize under the investigator's assessment;
- Remote follow-up methods include text messages, phone calls, videos, photos, etc. On-site visits are carried out according to the above monitoring process and actual conditions.

6.7. Safety observations

All participants need to be followed up for safety:

- **Safety observation from 0 to 28 days after booster vaccination**

All participants will collect all adverse events occurring within 30 minutes after vaccination, solicited inoculation site (local) and non-inoculation site (systemic) adverse events within 0-14 days, and unsolicited adverse events within 0 to 28 days.

- **Long-term safety observation**

All serious adverse events (SAEs), adverse events of special interest (AESIs), and pregnancy-related events within 6 months after vaccination will be collected from all participants.

6.8. Immunogenicity observation (for immunogenicity subgroup only)

For participants in the immunogenicity subgroup (100 cases), venous blood samples of approximately 15 mL will be collected before booster vaccination, 7 days, 14 days, 28 days, 3 months, and 6 months after booster (a total of 6 times). Separated serum will be detected for the virus-neutralizing antibody against the current main epidemic strain and specific S-protein IgG antibody.

Extra venous blood samples of the participants in the immunogenicity subgroup (100 cases) will be collected before vaccination, 7 days, 14 days, 28 days, and 3 months after booster (5 times in total) to detect cytokines of IL-2, IL-4, IL-13, and IFN-γ (ELISpot).

6.9. Follow-up plan

Within 28 days after the booster vaccination, all participants need to conduct at least 3 on-site visits to complete enrollment (visit 1), vaccination (visit 1), distribution, and collection of diary cards and contact cards (visit 1, visit 3, visit 4). The participants in the immunogenicity subgroup also need to collect blood for immunogenicity (visit 1-6).

From the 7th day to the 180th day after the booster vaccination, all participants

need to conduct remote visits at least 22 times (about 8 times per month from the 7th day to the 90th day after the vaccination; about once per month from the 90th day to the 180th day after the vaccination), to carry out routine monitoring of VE and/or case monitoring after booster vaccination. At the same time, AEs, SAEs, AESIs, and pregnancy events (including pregnancy outcomes, delivery characteristics, the condition of the newborn, and the growth and development within 1 month after birth) need to be collected.

In addition to the on-site visits and remote visits mentioned above, the investigators may increase the on-site visits or remote visits to the participants to an appropriate frequency according to actual needs, and make follow-up records.

6.10. Data collection

The necessary data for statistical analysis will be collected using an electronic data capture (EDC) system.

6.11. Randomization and Blinding

6.11.1. Randomization

The randomization statistician will use SAS statistical software and generate randomization blind code with the method of "block randomization". After the investigator confirms that the participant is eligible for inclusion and signs the informed consent, the participant will be assigned a randomized study number (randomization number). The investigator will choose the investigational vaccine or placebo with the same number as the randomization number and give the injection to the participant. For the randomized participants who withdraw from the clinical study for any reason, regardless of whether they have been vaccinated with the investigational vaccines before, their randomization numbers will be retained and not be distributed again, and the participants will not be permitted to participate in this study.

Back-up vaccines: The block randomization method will be used to generate a randomization list of back-up vaccines, with a total of 320 vaccines (160 study

vaccines and 160 placebos). When the backup vaccine is used, the investigator should log into the back-up vaccine system to obtain the corresponding number of the back-up vaccine.

6.11.2. Blinding

An observer-blinded design will be used for this study. Before the study, the randomization statistician will provide the randomization table to the unblinded investigators who are authorized to perform vaccination-related work, and the unblinded investigators will give the injections according to the groups corresponding to the randomization numbers in the randomization table. Because the LVRNA021 vaccine and placebo are different in physical appearance, the investigators should set up blinding measures, including but not limited to syringes shading, room partitions, eye masks, etc., to prevent the participants from identifying the type of investigational vaccines based on their appearances.

The duties of drug dispensing and administration in the unblinded team must be assigned to one or more investigators who are not involved in any study of participants' evaluation. Unblinded teams and managers should avoid touching the participants as much as possible. Blind must be maintained during the generation of randomization numbers and the preparation of blind codes, the random allocation of vaccines, the vaccination of the participants, the results recording and efficacy evaluations of the investigators, the monitoring of the monitors, and data management.

Due to the inherent difference in liquid color between the study vaccine and the placebo, to ensure the feasibility of blinded design, it is necessary to appoint unblinded study staff outside the investigator's observation team for vaccination, and they are required not to disclose the participant grouping information to other relevant staff involved in the trial. To ensure that the participants are blinded, during the trial, the risk of the subjects guessing the group from the appearance of the investigational vaccine should be avoided, which may cause bias in the evaluation of safety or efficacy. Investigators who have been authorized to perform vaccination shall not be authorized to engage in tasks that may lead to unblinding such as subject blood

sample collection, efficacy follow-up, or safety follow-up, to ensure the blind maintenance during subject follow-up and immunogenicity blood sampling. For other specific operation instructions, please refer to the Pharmacy Manual.

6.11.3. Vaccine blinding

The vaccines used in this study will not be blinded.

6.11.4. Emergency unblinding

Only in emergencies or when serious adverse events occur, and the information of the investigational product is critical to the clinical treatment or health of the participants, it is necessary to conduct individual emergency unblinding and make relevant records, after the confirmation by the principal investigator and sponsor. The participant with this study number will terminate the study as a dropout, and the investigator will record the reason for termination in the "Vaccination and Follow-up Record Book".

If group adverse events occur or the study needs to be interrupted for any reason, the unblinding could be done in advance with the approval of the sponsor.

6.11.5. Unblinding regulations

If the primary objectives are achieved or the sponsor decides to terminate the research, the data manager will clean up the data and then lock the database. Unblinding can only be performed after the database is locked and the group of participants corresponding to the study number can be revealed.

6.11.6. Blind maintenance

During the implementation of this clinical research, observer blindness should be maintained, that is, the participants, the observers(researchers) of safety or efficacy, and the staff of laboratory test are all blinded. The staff responsible for vaccine management and vaccination are unblinded.

Unblinded staff who are authorized for vaccine management, vaccination, unblinded data management, unblinded quality control(QC), and unblinded monitoring are required to sign a confidentiality agreement and promise not to disclose any information that may cause blindness breaking to other personnel (including the investigators involved, the participants, the staff for serum sample

detection, the blinded monitors, QC staffs, and data management staffs, etc.). At the same time, unblinded staff are no longer authorized to participate in other works that may lead to blindness breaking, such as safety observation.

The monitoring and QC works involving vaccine management and vaccination will be completed by unblinded monitors and QC staff.

When the vaccines are transported to the clinical research site, they will be properly managed by the vaccine/drug administrators on site.

Other biological samples such as blood are only marked with the code related to the study number, to ensure that the detection is blinded.

After the study is unblinded, the research at the clinical site will enter into the open stage, but the detection remain blinded. Anyone is forbidden to disclose any information that may cause blindness breaking to the detection agencies to ensure the objectivity of the testing data.

6.12. Study suspension or termination criteria

The sponsor reserves the right to suspend/terminate the study at any time. After full consultation with the sponsor and the sponsor’s consent, the investigator could suspend or terminate the work of the research center. Reasons for suspending or terminating the study may be, but are not limited to, the following:

(1) It is clear that the investigational vaccine lacks efficacy;

(2) The incidence or severity of adverse reactions indicate that there may be potential dangers that may endanger the life of the participants;

(3) The sponsor suspends or terminates the research voluntarily;

(4) Suspend or terminate due to regulatory agency requirements.

During the study, if any of the criteria triggering the study suspension occurs, the safety of the investigational vaccine will be assessed by the investigator.

6.13. Confidentiality agreement and privacy of the participants

The investigators shall undertake to keep confidential to third parties any confidential information obtained from the sponsors or investigational products, or

provided or disclosed in the current contract, and to use such information within the agreed scope of the agreement.

As long as the sponsor has a reasonable and justifiable reason to require the investigator to maintain the confidentiality of the agreement, the agreement should be independent and valid for the duration of the contractual relationship between the two parties.

The investigators must ensure the privacy of the participants in the clinical research. In all the documents submitted to the sponsor, the identity of the participants can only be determined by the subject codes of the clinical research, and the names of the participants cannot be indicated. The investigators must properly keep the names and addresses of the participants and the enrollment form corresponding to the subject codes. These enrollment forms are kept strictly confidential by the investigator and cannot be submitted to the sponsor.

6.14. Study Duration

All participants will be in the study for about 6-7 months.

7. Study population

7.1. Inclusion criteria

Participants must meet all the following inclusion criteria:

(1) Both male and female adults aged 18 years and above who can provide their identification;

(2) Understand the contents of the ICF and the vaccine for this vaccination, sign the ICF, and have the ability to use a thermometer, scale, and fill in the diary card and contact card as required;

(3) Be able to communicate well with the researcher and understand and comply with

the requirements of this study;

(4) Participants must have received 2 or 3 doses of SARS-CoV-2 inactivated vaccine, with the last dose received at least 6 months before enrolment.

(5) Healthy participants or participants with mild underlying disease [in a stable state without exacerbation (no admission to hospital or no major adjustment to treatment regimen, etc.) for at least 3 months before enrollment in this study].

(6) For female participants: without childbearing potential (amenorrhea for at least 1 year or documented surgical sterilization) or not in pregnancy or lactation and have used effective contraception (e.g., intrauterine or implantable contraceptive devices, oral contraceptives, injectable or buried contraceptives, sustained-release topical contraceptives, intrauterine devices (IUDs), condoms (for males), diaphragms, cervical caps, etc.) for the past 14 days before the first dose of vaccine (provide negative pregnancy certificate within 48 hours);

(7) Negative results within 48 hours for SARS-CoV-2 pathogenicity testing (RT-PCR).

7.2. Exclusion criteria

Participants meeting any of the following criteria will be excluded from the study.

(1) Abnormal vital signs that are clinically significant (e.g., blood pressure that is still abnormal after control);

(2) COVID-19 infection in the last 6 months or use of any COVID-19 prophylactic medication other than 3 doses of inactivated SARS-CoV-2 vaccine (e.g., a history of vaccination with any other non-inactivated SARS-CoV-2 inactivated vaccine, marketed or unlisted, or vaccination with 1 or 4 doses of inactivated SARS-CoV-2 vaccine)

(3) A history of Severe Acute Respiratory Syndrome (SARS), Middle East Respiratory Syndrome (MERS), or other coronavirus infections at any time.

(4) Axillary temperature ≥37.3°C detected on the day of vaccination or fever within the last 24 hours (axillary temperature ≥37.3°C/oral temperature ≥37.5°C);

(5) A history of severe allergic or allergic reactions to vaccines or drugs, e.g., urticaria, severe skin eczema, dyspnea, laryngeal edema, angioneurotic edema, etc.

(6) Any other licensed vaccines given within 28 days before study vaccination;

(7) Participation in a clinical study of other drugs within 28 days before vaccination or planned participation within 6 months after vaccination;

(8) Have an inherited bleeding tendency or coagulation disorder (eg: Cytokine deficiency, coagulopathy, or thrombocytopenia), or a history of severe bleeding;

(9) Diseases affecting the functioning of the immune system (e.g., cancer, except basal cell carcinoma of the skin), congenital or acquired immunodeficiencies (e.g., HIV infection), uncontrolled autoimmune diseases, etc., based on a known medical history or diagnosis;

(10) Asplenia or functional asplenia;

(11) Long-term use (continuous use for ≥14 days) of immunosuppressants or other immunomodulatory drugs (eg, corticosteroids: prednisone or equivalent; interferon, etc.) within 6 months before study vaccination. Topical medications (e.g., ointments, eye drops, inhalers, or nasal sprays) are permitted and should not exceed the dose recommended in the instructions;

(12) Receipt of immunoglobulins and/or blood products within 3 months before study vaccination;

(13) Suspected or known alcohol dependence or drug abuse;

(14) Participants were deemed unsuitable for participation in this study based on the investigator's assessment.

7.3. The criteria for early termination of the study:

(1) The participant has a disability, life-threatening adverse events, or serious adverse events, and needs to withdraw from the study in advance due to other reasons such as the need for treatment;

(2) The participant's health condition is at risk for safety and does not allow him to continue to participate in this study;

(3) Female participants are pregnant (if the vaccination has been completed, the

participant does not need to be withdrawn from the study, and follow-up observation is required);

(4) The participant voluntarily requests to withdraw from the clinical study;

(5) The investigator considers the subject unsuitable for further participation in the clinical study.

7.4. Participant withdrawal from the study:

Participant withdrawal from the study: the participant cannot continue to complete the visits specified in the protocol due to reasons such as meeting the criteria for early termination of the study or being unable to be contacted. Including but not limited to:

(1) The participant could not complete the follow-up visit because they met the 7.3 criteria for early termination;

(2) The participant could not be contacted within the visit time scheduled in the study protocol, and it is confirmed that the subject cannot attend all subsequent visits;

(3) The participant permanently left the original place of residence and lost contact.

The investigator should inform the participant of the right to withdraw from the study at any time. The withdrawn participants will not be replaced, and the investigator should try to contact the participants who failed to return to follow-up at the scheduled time. All data collected before withdrawal could be used for analysis. When a participant withdraws before study completion, the date and reason for withdrawal is to be documented in the eCRF and the source document, with a detailed description of the situation:

(1) Serious adverse events

(2) Adverse events (but not serious adverse events)

(3) Protocol violations

(4) Voluntary withdrawal, but not due to adverse events

(5) Going out, migrating, or moving away from the study site

(6) Loss to follow-up

(7) Death

(8) Other

A clear distinction must be made between subjects who withdraw from the study due to an adverse event and those who withdraw for other reasons. After withdrawal or termination of the study for the participants who have received the study vaccine, the investigator shall provide necessary treatment for the study-related clinical conditions of the participants, and follow up on the adverse events/serious adverse events until the definite diagnosis/stable disease/recovery.

Definition of withdrawal time: the time when the investigator decides to terminate the participant from the study or the time when the participant voluntarily withdraws from the study.

Definition of completion of clinical study: The clinical study is considered to be completed within 6 months after the subject completes the protective efficacy and safety visit after the booster immunization.

The study population was 18 years of age and older who had completed 2 or 3 doses of New Crown inactivated vaccine for ≥6 months.

8. Lost to follow-up, protocol deviation/violation, pregnancy

8.1. Lost to follow-up

When the subject can not return on time for a study visit, with full respect for the participant's rights, the investigator should make an effort to contact or recall the participant, or at least determine the participant's health status, and document the efforts made (e.g., phone call and text message logs).

8.2. Protocol deviation/protocol violation

Protocol deviation: refers to any change and non-compliance with the clinical research protocol design or process and not approved by the Clinical Medical Research Ethics Committee. The behavior that did not affect the participant's benefits, safety, completeness, accuracy, and reliability of the study data was a mild protocol deviation. The behavior that affects the benefits, safety, and safety of the participants, or the completeness, accuracy, and reliability of the study data, is a serious protocol deviation (protocol violation)

If it is determined to be a deviation, and the deviation may affect the safety of participants, participant eligibility, or data reliability after assessment, the CRC, investigator, or designee shall report to the Clinical Research Ethics Committee and the sponsor as soon as possible; If the deviation has little or no impact on the above matters, it can be reported to the clinical medical research ethics and the sponsor after the end of the study.

Refer to the Medical Monitoring Plan and Protocol Deviation List for details.

8.3. Pregnancy

The investigator shall collect the pregnancy events within 6 months after booster vaccination in female participants. Complete the Pregnancy Event Report Form within 24 hours after learning that the participant is pregnant. and report by mail to the PV department of the CRO team representing the sponsor. All pregnant women within the collection period of pregnancy events will be followed up until the end of pregnancy or 1 month after the birth of the newborn, and the records will include pregnancy outcome, delivery mode, and clinical characteristics (duration of pregnancy, outcome), birth status (gender, weight, height, newborn score) and growth and development within 1 month after birth (such as congenital anomaly or birth defect of

newborn shall be regarded as SAE). Pregnancy is not considered an SAE in this study, but any complication during pregnancy will be considered as an AE and in some cases can be considered as an SAE, such as spontaneous abortion, stillbirth, stillbirth, and congenital anomalies of infants. When no abnormality is found in the fetus, the induced abortion due to the mother's personal decision is not considered an adverse event. If the induced abortion event meets the SAE criteria, it is necessary to report the SAE.

9. Vaccines, vaccine transportation, storage and administration

9.1. Investigational technology

Study vaccine: SARS-CoV-2 variant mRNA vaccine (LVRNA012)

Provided by: AIM Vaccine Co., Ltd.

Dosage: 100 μg, 1.0 mL/dose

Appearance: colorless or slightly milky white liquid

Dosage form: Injection

Route of administration: intramuscular injection into the lateral deltoid muscle of the upper arm

Immunization schedule: 1 dose

Storage and transport conditions: Store and transport at-20℃±5℃.

Control vaccine:

Placebo: Saline

Provided by: AIM Vaccine Co., Ltd.

Dosage: 0.5mL/dose

Dosage form: Injection

Appearance: Clarified colorless liquid

Route of administration: intramuscular injection into the lateral deltoid muscle of upper arm

Immunization schedule: 1 dose

Storage and transportation conditions: Store and transport at-20℃±5℃.

9.2. Storage and Transportation of Vaccines

The study vaccine and placebo should be stored and transported at 20 (±5) ℃. The storage temperature needs to be monitored and recorded daily. On the premise of maintaining automatic temperature monitoring and alarm, the temperature shall be recorded according to the specific conditions of the study site on holidays. If the storage and transport process is out of the specified temperature range, the site study pharmacist should immediately isolate the over-temperature study vaccine at -20 (±5) ℃, contact the sponsor and the study monitor, and not use the study vaccines which experienced temperature excursion until advice is received from the sponsor on how to proceed.

9.3. Route of Vaccination and Immunization Procedure

Site and route of administration: The site of administration is the lateral deltoid muscle of the upper arm, and the route of administration is intramuscular injection.

Immunization procedure: The participant assigned to the study vaccine group received 1 dose of the study vaccine on Day 0. The participants assigned to the placebo group received 1 dose of placebo on day 0.

9.4. Backup vaccine

When the backup vaccine is needed in case of vaccine damage in the study, please refer to the corresponding SOP.

9.5. Vaccine number.

The vaccine in this clinical study has a unique number in the format of 0001 and 0002.

9.6. Vaccine allocation

The study number will be assigned according to the enrollment order of the screened participants, and the screening number and the initials of the participants will be filled in the Randomization Table. The study vaccine or placebo will be obtained and administered according to the study number.

9.7. Vaccine packaging and labeling

The study label shall include the protocol number, the name of the study vaccine, the name of the company, the expiry date and batch number, the words For clinical research only, and the vaccine number, such as 0001,0002.

10. Concomitant medication:

Concomitant medication: Concomitant medication refers to all drugs other than investigational vaccines which are used from the day of vaccination to 28 days after vaccination, including antibiotics, antivirals, antipyretic analgesics, anti-allergy drugs, biological products (including vaccine, immunoglobulin, blood products, etc.), traditional (patent) medicine (except vitamins and/or food additives for non-therapeutic purposes), etc. Information on concomitant medications includes the name of the drug, dosage, administration date, and indication.

Permitted medication: During the study, participants should be allowed to take necessary medication in case of adverse events.

Prophylactic drugs: mean the drug given when there are no symptoms or anticipated occurrence of vaccination reactions. For example, If antipyretics are given for fever

prevention in the subjects without fever during the recruitment period, antipyretics are considered a type of preventive drug. If treatment with drugs is required due to the occurrence of an adverse event, there is no restriction. At enrollment, the participants are inquired about the ongoing drugs to confirm that antipyretics, analgesics, or anti-allergic drugs are not given.

Permissible vaccines: Only vaccines for emergency use (e.g., tetanus vaccine or rabies vaccine) are allowed within 28 days after vaccination. If any other vaccine is administered, it will be recorded and reported as a protocol violation. After the completion of the investigational vaccination, in case of emergency vaccination, the vaccine can be used according to the product instructions, and the safety should be closely monitored and recorded in detail. If the vaccination interval is less than 28 days, record and report a protocol violation.

Recording of medication: To assess the impact of medications used during the study on vaccine safety, or to collect all the adverse events that may be related to vaccination, the investigator must take measures to collect the medications taken by participants during the observation period. Safety information and concomitant medications will be collected from all participants of this study. Participants should be instructed to record all clinic visits and medications from the day of vaccination to 28 days after vaccination in the Diary Card and Contact Card, and the medications recorded by participants should be reviewed.

11. Study methods and procedures

11.1. Participant Recruitment

After the study sites are confirmed and approved by the IRB, IRB-approved advertisements will be distributed to any population who meets protocol requirements for age, vaccination history, and health condition.

11.2. Study procedure

11.2.1. Visit 1 (Day 0)

(1) Informed consent and assignment of screening number

The researcher needs to fully inform the participants about the clinical research information. If the participants accept written informed consent, then need to sign the ICF. The ICF shall be made in duplicate, one for the study site and one for the participant. After signing the ICF, the researcher will assign a screening number to the participant and make copies of the participant's identification materials.

(2) Physical examination and screening.

The researchers need to perform a routine physical examination of the participant who has signed ICR, observing the general condition, respiration, head and neck, lymph node, skin, etc., and take measures of their height, weight, axillary temperature, pulse, and blood pressure. Participants are required to provide a negative nucleic acid test report within 48 hours, and female participants of childbearing age are required to provide a negative pregnancy test certificate within 48 hours. Confirm the vaccination record of SARS-CoV-2 vaccines of the participant by checking the participant's health code, etc., and then determine whether the participant can be included in the clinical study according to the inclusion and exclusion criteria in combination with the inquiry records and measurement results.

(3) Assignment of random numbers

Participants who meet the inclusion criteria and do not meet the exclusion criteria as determined by the researcher through physical examination and consultation can be enrolled. The researcher shall fill in the Randomization Form with the initials and screening number of the enrolled participants, and sign the name and date; At the same time, assign a study number to the participant according to the enrollment sequence of the participant, and fill the study number in the Vaccination and

Follow-up Record.

(4) Blood collection before vaccination (only the immunity subgroup)

Blood collection for humoral immunogenicity test: about 5.0 mL venous blood is collected from the participant with a procoagulant tube (containing only procoagulant), and the serum is separated according to the operating procedures and sub-packaged into cryopreservation tubes.

Blood collection for cellular immunity test: about 15.0 mL venous blood is collected from the participant with a heparin anticoagulant tube

(5) Vaccination

The vaccine preparation personnel shall obtain the vaccine with the corresponding number according to the study number of the subject, check that the information on the label of the vaccine box and the inner label is correct and consistent, and confirm that the vaccine status is normal. The vaccinator shall check the study number in the participant's Vaccination and Follow-up Record to confirm that it is correct.

The vaccinator shall check the participant information again before vaccination for each subject. After checking, the skin of the lateral deltoid muscle of the upper arm of the subject shall be disinfected with 75% medical alcohol, and the vaccine after the skin is slightly dry. After completion of vaccination, the investigator shall fill in the vaccination time, vaccination site, and other information in the corresponding position in the Vaccination and Follow-up Record, and sign the name and time.

(6) Safety observation after vaccination

All participants will be observed at the study site for at least 30 minutes after vaccination and adverse events will be recorded. Investigator should distribute the Diary Card, thermometer, and scale to the participants, train the participants on how to use the thermometer and scale to observe adverse events, and how to fill in the

Diary Card and make an appointment for the participants to return the Diary Card.

The investigator should highlight the investigator's phone number on the copy of the ICF and Diary Card to the participants and instruct them to contact the investigator immediately if they experience any signs, symptoms, or events requiring hospitalization that they consider serious after vaccination.

11.2.2. Visit 2 (Day 7+2 after vaccination) (only the immunity subgroup)

(1) Blood collection

Blood collection for humoral immunogenicity test: about 5.0 mL venous blood is collected from the participant with a procoagulant tube (containing only procoagulant), and the serum is separated according to the operating procedures and sub-packaged into cryopreservation tubes.

Blood collection for cellular immunity test: about 15.0 mL of venous blood is collected from the participant with a heparin anticoagulant tube.

(2) Safety observation

Collect concomitant medications, AE, SAE, AESI, and pregnancy events.

(3) COVID-19 case observation

Determine whether the participant presents with COVID-19-related signs or symptoms.

11.2.3. Visit 3 (Day 14+7 after vaccination)

(1) Collection and review of Diary Card

Participants return the Diary Card. The investigator should review and confirm whether the AE or concomitant medications are completely and correctly recorded in the Diary Card together with the participant, and instruct the participants to make corrections and supplements, then recycle the Diary Card.

(2) Blood collection

Blood collection for humoral immunogenicity test: about 5.0 mL venous blood is collected from the participant with a procoagulant tube (containing only procoagulant), and the serum is separated according to the operating procedures and sub-packaged into cryopreservation tubes.

Blood collection for cellular immunity test: about 15.0 mL of venous blood is collected from the participant with a heparin anticoagulant tube.

(3) Training and Issuance of Contact Card

Participants will be given a Contact Card and trained to record all AE and concomitant medications (if applicable) from 15 to 28 days after vaccination.

(4) Safety observation

Collect concomitant medications, AE, SAE, AESI, and pregnancy events.

(5) COVID-19 case observation

Determine whether the participant presents with COVID-19-related signs or symptoms.

11.2.4. Visit 4 (Day 28+7 after vaccination)

(1) Collection and review of Contact Card

Participants return the Contact Card. The investigator should review and confirm whether the AE or concomitant medications are completely and correctly recorded in the Contact Card together with the participant, and instruct the participants to make corrections and supplements, then recycle the Diary Card.

(2) Blood collection

Blood collection for humoral immunogenicity test: about 5.0 mL venous blood is collected from the participant with a procoagulant tube (containing only procoagulant),

and the serum is separated according to the operating procedures and sub-packaged into cryopreservation tubes.

Blood collection for cellular immunity test: about 15.0 mL of venous blood is collected from the participant with a heparin anticoagulant tube.

(3) Safety observation

Collect concomitant medications, AE, SAE, AESI, and pregnancy events.

(4) COVID-19 case observation

Determine whether the participant presents with COVID-19-related signs or symptoms.

11.2.5. Visit 5 (Day 90+15 after vaccination) (only the immunity subgroup)

(1) Blood collection

Blood collection for humoral immunogenicity test: about 5.0 mL venous blood is collected from the participant with a procoagulant tube (containing only procoagulant), and the serum is separated according to the operating procedures and sub-packaged into cryopreservation tubes.

Blood collection for cellular immunity test: about 15.0 mL of venous blood is collected from the participant with a heparin anticoagulant tube.

(2) Safety observation

Collect SAE, AESI, and pregnancy events.

(3) COVID-19 case observation

Determine whether the participant presents with COVID-19-related signs or symptoms.

11.2.6. Visit 6 (Day 180+30 after vaccination) (only the immunity subgroup)

(1) Blood collection

Blood collection for humoral immunogenicity test: about 5.0 mL venous blood is collected from the participant with a procoagulant tube (containing only procoagulant), and the serum is separated according to the operating procedures and sub-packaged into cryopreservation tubes.

(2) Safety observation

Collect SAE, AESI, and pregnancy events.

(3) COVID-19 case observation

Determine whether the participant presents with COVID-19-related signs or symptoms.

11.2.7. Protective efficacy visits (from the day of vaccination to the 180th day after vaccination)

The routine surveillance and case surveillance are carried out according to the procedure of *6.6 observation of protective efficacy.*

11.2.8. End of study

The site closure report will be issued when the data entry in EDC is completed, the queries are resolved, the database is locked and all the materials are reviewed.

11.3. Methods of observation of protective efficacy

11.3.1. routine monitoring

7 days after booster vaccination in all subjects, the researchers were required to conduct the following"Routine monitoring" in the form of a combination of remote and on-site visits:

- Whether the subjects had COVID-19-related symptoms during the remote visit by the researchers;
- A throat swab sample a1(for SARS-CoV-2 nucleic acid or antigen detection) is

taken when the researcher is informed through a remote visit that the subject has one or more of the following conditions, at the same time further inquiry, record and guide the subject to continue observation, record the type of symptoms, starting and ending time, the severity of information; If an on-site interview is conducted (the on-site interview can be conducted by visiting the study site or by visiting the researcher), a nucleic acid throat swab B 1(backup) :

① any possible signs or symptoms of COVID-19(fever or chills, fatigue, headache, dry throat, muscle pain, sore throat, nasal congestion/runny nose, anorexia/nausea/vomiting, diarrhea, new onset of smell/taste disorder, conjunctivitis) ;

② any or more respiratory symptoms (cough, shortness of breath, or dyspnea), lasting at any time;

③ clinical or imaging evidence of pneumonia.

11.3.2. Case surveillance

If the test result for SARS-CoV-2 nucleic acid or antigen in the throat swab sample A1 of the subject is positive, the researcher needs to continue to carry out the following "case monitoring" work:

- Send the throat swab sample B1 of the subject's nucleic acid (if collected) to the central laboratory for SARS-CoV-2 variant typing testing, guide and coordinate the subject to go to a designated hospital for chest imaging examination (chest CT);
- Provide necessary medical supplies to the subjects, continue to conduct remote visits twice a week, guide them to observe and record symptoms and self-medication at home, or guide them to seek medical treatment at designated hospitals according to the situation; If the subject has symptoms such as Tachypnea or dyspnea, guide and coordinate the subject to detect blood oxygen saturation according to the situation; According to the recovery of symptoms of the subjects, conduct on-site visits, collect samples of nucleic acid oropharyngeal swabs or antigen Nasopharyngeal swab for SARS-CoV-2 nucleic acid or antigen detection until the results of SARS-CoV-2 nucleic acid or antigen turn negative

or the symptoms disappear or stabilize according to the evaluation of the investigator;

- Collect the treatment status and medical history of the subjects to determine the severity of COVID-19, record the type of symptoms, occurrence time, reporting time, collection time, testing time, reporting time, diagnosis time, and outcome information of each nucleic acid sample, and form relevant records;
- Establish a COVID-19 case file for the subjects, fill out and report the first/follow-up COVID-19 case report based on remote visits;
- The on-site and medical monitoring teams need to conduct a preliminary review of the completeness, rationality, and logicality of the COVID-19 case report, raise necessary questions and information supplement requirements, and submit it to the researchers for diagnosis and judgment.

If the test result of the throat swab sample A1 for SARS-CoV-2 nucleic acid or antigen is negative, the following retesting work needs to be continued:

- Within 48 hours, the throat swab sample A2 (for SARS-CoV-2 nucleic acid or antigen testing) and sample B2 (backup sample) need to be collected again;
- If the test result for SARS-CoV-2 nucleic acid or antigen in the throat swab sample A2 of the subject is positive, the relevant work shall be carried out according to the above case monitoring process;
- If the test result for SARS-CoV-2 nucleic acid or antigen in the throat swab sample A2 of the subject is still negative, the relevant work will continue according to the routine monitoring process mentioned above.

Frequency and method of "visit":

- Starting from 7 days after the booster vaccination, remote visits will be conducted twice a week (with a recommended interval of 2-3 days) until the 90th day after vaccination: (1) If the number of confirmed primary endpoint cases reaches 162, the visit video rate will be adjusted to once a month until the 180th day after vaccination; (2) If the number of confirmed primary endpoint cases does not

reach 162, remote visits will continue to be conducted twice a week until the number of primary endpoint cases reaches 162, and the video visit rate will be adjusted to once a month until the 180th day after vaccination;

- If the subject's SARS-CoV-2 nucleic acid or antigen test results are positive, regardless of the stage mentioned above, they will be visited twice a week (with a recommended interval of 2-3 days) until their nucleic acid or antigen test results turn negative or symptoms disappear or stabilize after evaluation by the researcher;
- Remote follow-up methods include text messages, phone calls, videos, and photos, and on-site visits are conducted based on the monitoring process and actual situation mentioned above.

11.4. Safety observation method

11.4.1. Safety observation time and method

(1) Within 28 days after vaccination

Participants will be observed at the vaccination site for 30 minutes after vaccination. Investigators who have been trained and authorized need to systematically observe and measure the body temperature of each participant 30 minutes after vaccination, and record all AEs within 30 minutes after vaccination in detail. AEs will be observed and recorded through the Diary Card from 0 to 14 days after vaccination, and AEs will be collected and recorded through the Contact Card from 15 to 28 days after vaccination.

Investigators should check the original safety information of the Diary Card and Contact Card in time and input it into EDC after checking. Investigators should verify the name, duration, and severity of any AE and evaluate and determine its relationship to vaccination. Measures taken in response to the AE, combined treatment, and outcome of the AE, whether the event was a serious adverse event, and whether the event led to study discontinuation, must also be documented.

(2) Long-term safety observation

SAE, AESI, and pregnancy-related events should be collected and recorded for all participants 6 months after vaccination through a combination of remote visits and participant active reporting. SAE, AESI, and pregnancy-related events collected need to be followed up, recorded, and reported following SAE and pregnancy-related event reporting in this protocol.

11.4.2. Safety observation content and indicators

Routine safety observation content includes all solicitation and non-solicitation, as well as any medical events related to or unrelated to vaccination that occur during the clinical study observation period.

Injection site (local) AE: Pain, induration, redness, rash, swelling, pruritus, cellulitis.

Non-injection site (systemic) AE: fever (axillary temperature), diarrhea, nausea, vomiting, headache, myalgia (non-injection site), arthralgia, chills, anorexia, fatigue/asthenia, acute allergic reaction

Other events: any medical event other than the above, such as acute illness, accidental injury, etc.

11.4.3. Safety observation grading standards.

Adverse events at the injection site and non-injection site (Table 2 and Table 3) are judged regarding the Guidelines for Grading Criteria of Adverse Events in Clinical Trials of Preventive Vaccines issued by NMPA. For adverse events not mentioned in the grading table, the intensity will be assessed according to Table 4.

Table 2 Grading of Injection Site (Local) Adverse Events

Symptoms/Signs	Grade 1	Grade 2	Grade 3	Grade 4
Pain	No influence or mild influence on physical	Influence on physical activities	Influence on daily activities	Loss of basic self-care ability, or

	activities			hospitalization
Induration*, Swelling** #	Diameter of 2.5 - < 5 cm or area of 6.25 - < 25 cm^2 and no influence or slight influence on daily living	Diameter of 5 - < 10 cm or area of 25 - < 100 cm^2 or influence on daily living	Diameter of ≥ 10 cm or area of ≥ 100 cm^2 or ulceration or secondary infection or phlebitis or aseptic abscess or wound drainage or serious influence on daily living	Abscess, exfoliative dermatitis, dermis, or deep tissue necrosis
Rash*, redness** #	Diameter of 2.5 - < 5 cm or area of 6.25 - < 25 cm^2, and no influence or slight influence on daily living	Diameter of 5 - < 10 cm or area of 25 - < 100 cm^2, or influence on daily living	Diameter of ≥ 10 cm or area of ≥ 100 cm^2 or ulceration or secondary infection or phlebitis or aseptic abscess or wound drainage or serious influence on daily living	Abscess, exfoliative dermatitis, dermis, or deep tissue necrosis
Pruritus	Injection site pruritis, relieved spontaneously or within 48 h after treatment	Injection site pruritis is not relieved within 48 h after treatment	Influence on daily living	NA
Cellulitis	NA	Non-injection therapy indicated (e.g., oral anti-bacterial, anti-fungal, anti-viral drug therapy)	Intravenous injection therapy indicated (e.g., intravenous anti-bacterial, anti-fungal, antiviral drug therapy)	Sepsis, tissue necrosis, etc.

Note: *In addition to direct measurement of diameter for grading evaluation, changes in measurement results are also recorded.

**Use the maximum measured diameter or area.

#Evaluation and grading of induration and swelling, rash, and redness are based on functional level and actual measurement results, the parameters with higher grades are selected.

Table 3 Grading of (Systemic) AEs at Non-injection Site

Signs	Grade 1	Grade 2	Grade 3	Grade 4
Fever [axillary temperature (℃)]	37.3~<38.0	38.0~<38.5	38.5~<39.5	≥ 39.5, lasts for longer than 3 days
Diarrhea	Slight or transient, 3-4 times/day, abnormal stool appearance, or slight diarrhea lasting less than 1 week	Moderate or persistent, 5-7 times/day, abnormal stool appearance, or diarrhea > 1 week	> 7 times/day, abnormal stool appearance, or bloody diarrhea, orthostatic hypotension, electrolyte imbalance, with > 2 L intravenous infusion indicated	Shock due to hypotension, requiring hospitalization
Nausea	Transient (< 24 h) or intermittent and normal food intake	Persistent nausea resulting in food intake decreased (24 - 48 h)	Persistent nausea resulting in almost no food intake (> 48 h) or intravenous fluids replacement required	Life-threatening (e.g., hypotensive shock)
Vomiting	1-2 times/24 h and no influence on activities	3-5 times/24 h or limited activities	> 6 times/24 h or intravenous fluids replacement required	Shock due to hypotension, requiring hospitalization or other routes of nutrition
Anorexia	Decreased appetite, but no reduction in food intake	Decreased appetite, and decreased food intake, but no significant weight loss	Decreased appetite, with significant weight loss	Requiring intervention measures (such as intragastric tube feeding and parenteral nutrition)

Headache	No influence on daily activities, treatment not indicated	Transient, slight influence on daily activities, treatment, or intervention indicated possibly	Serious influence on daily activities, treatment, or intervention indicated	Stubborn, emergency treatment or hospitalization indicated
Myalgia (non-injection site)	No influence on daily activities	Slightly influence daily activities	Severe muscle pains, seriously influence daily activities	Emergency treatment or hospitalization
Arthralgia	Mild pain, without impairing function	Moderate pain; requiring analgesics and/or impairing function, but no influence on daily activities	Severe pain; requiring analgesics and/or influence on daily activities	Disabling pain
chill	A mild feeling of cold, teeth chattering	The whole body shivers moderately	Severe/persistent tremor	Emergency treatment or hospitalization
Fatigue, asthenia	No influence on daily activities	Influence on normal daily activities	Serious influence on daily activities, unable to work	Emergency treatment or hospitalization
Acute allergic reaction*	Urticaria localized (blister), treatment not indicated	Urticaria localized, treatment indicated or mild angioedema, treatment not indicated	Extensive urticaria or angioedema, treatment indicated, or mild bronchospasm	Allergic shock or life-threatening bronchospasm or laryngeal edema

* refers to type I hypersensitivity.

Table 4 Grading of Other Adverse Events

Grade 1	Grade 2	Grade 3	Grade 4	Grade 5

Mild: Short-term (< 48 h) or mild discomfort, no influence on activities, treatment not indicated	Moderate: mildly or moderately restricted activity, medical attention indicated possibly, no treatment or mild treatment indicated	Severe: significantly restricted activity, medical attention and treatment indicated, hospitalization indicated possibly	Critical: may be life-threatening, severely restricted activity, intensive care indicated	Death

11.4.4. Relationship between adverse events and vaccine

Investigators should have measures to determine the causality of all AE and abnormal laboratory indicators to vaccination promptly, to identify SAEs and groups, convergent AE related to vaccination during clinical studies, and to suspend and terminate clinical studies on time to minimize harm to participants. Whether the adverse event is related to vaccination and the degree of correlation shall be determined according to the following principles.

Not related: the participant did not use the investigational vaccine, the AE occurred in an illogical chronological sequence with the administration of the investigational vaccine, or other significant causes could have led to the AE.

Unlikely related: evidence of investigational vaccination; AE more likely to be due to other causes; repeat investigational vaccination negative or inconclusive

Possibly related: evidence of investigational vaccination; temporal sequence of AE to investigational vaccination is reasonable; AE cannot be ruled out as being caused by

the investigational vaccine but may also be due to other causes.

Probably related: evidence of investigational vaccination; the chronology of AE with investigational vaccination is reasonable; AE is more plausibly explained by investigational vaccine than other causes.

Related: evidence of vaccination with the investigational vaccine; the temporal sequence of the AE with the administration of the investigational vaccine is reasonable; the AE is more plausibly explained by the investigational vaccine than by other causes; repeated positive vaccination with the investigational vaccine; the AE profile is consistent with previous knowledge of this or this type of vaccine.

Of these, "definitely related", "probably related", and "possibly related" were classified as "vaccine-related". "Unlikely related" and "Not related" are classified as "not vaccine-related"

11.4.5. Outcome of AE

Outcome of AEs included: Recovered, Recovering, Unchanged, Recovered with sequelae, and Other (Death, Unknown).

11.5. Immunogenicity observation method

Participants in the immunity subgroup: about 5.0 mL of venous blood will be collected and placed in a procoagulant tube (containing only procoagulant) before vaccination, 7 days, 14 days, 28 days, 3 months, and 6 months after vaccination for detection of live virus neutralizing antibody and specific IgG antibody of current epidemic strain.

Participants in the immunity subgroup: An additional 15mL venous blood will be

collected before vaccination, 7 days, 14 days, 28 days, and 3 months after vaccination to detect IL-2, IL-4, IL-13, and IFN-γ cytokine levels (ELISpot method).

11.6. Biospecimen management

11.6.1. Handling, storage, and transportation of blood samples

Blood samples for humoral immunoassays: Venous blood is collected and waited for adequate clotting, then centrifuged to separate the serum within 4 hours. If the blood sample cannot be centrifuged on the same day, it needs to be stored in a refrigerator at 2~8℃ and the storage temperature needs to be recorded. The serums to be tested and the backup serums should be locked, managed by special personnel, and temperature recorded according to SOP requirements, stored at -20 ° C or below. The transport temperature should be kept below -20 ° C. The cold chain was abnormal when the temperature of serum was > -20℃ and < 0℃ during storage and transportation, and broken when the temperature was ≥0 ℃.

Blood samples for cellular immunity subgroup test: After venous blood is collected by anticoagulant tube, place it in a refrigerator at 2-8℃ for storage, record the storage temperature, and send it for testing as soon as possible.

11.6.2. Numbering rules of blood samples

The numbering rules of humoral immunity test blood sample labels are "random number-time point of blood collection-letter of serum submitted for test", for example, "0001-D0-A" means that tube A of the serum sent for testing which was collected by participant No. 0001 for testing the humoral immunogenicity tests before vaccination.

11.6.3. Sample detection

The humoral immunoassay is entrusted to a qualified third party. The specific IgG antibody titer will be detected by ELISA and the neutralizing antibody titer will be

detected by live virus neutralization assay. The testing reference material and quality control standards will be provided by the testing laboratory.

The cellular immunoassay is entrusted to a qualified third party. All relevant assays are detected using enzyme-linked immunospot assay (ELISpot). The testing reference material and quality control standards will be provided by the testing laboratory.

11.7. Data management

Data management plan: The data administrator writes the data management plan according to the clinical research protocol, and implements it after being reviewed and approved by the sponsor.

Establishment of the database: the eCRF is designed by the data administrator according to the clinical research plan, and the EDC is constructed by the database builder. The data administrator writes the data review plan (DVP) according to the plan and eCRF, and the database builder sets up logic verification according to the DVP. Before the EDC system goes online, it conducts a system test (UAT) and writes a test report. The EDC system that has passed the test can only be put into operation after being reviewed and approved by the sponsor.

Data entry: Data administrators write data entry guidelines and provide training to researchers and/or EDC entry personnel, who then enter EDCs by researchers and/or EDC entry personnel.

Authority distribution: After the EDC system is put into operation, according to the training records and account application status, the system administrator will create accounts and grant different permissions.

Data query: The data manager writes a data verification plan, which is implemented after approval by the funder. After the data is entered into the EDC, the system will check the data according to the Edit Check built into the data verification plan, and the data with doubts will be automatically sent to the system; the data that cannot be set to be questioned by the system will be sent to manual questions through the EDC, The input personnel or researchers confirm and answer the manual and system queries until the queries are resolved. When the answer fails to resolve the query, the data administrator and clinical monitor can re-query the data point, and all traces are stored in the EDC database.

External data: The data administrator writes an external data transmission agreement according to the plan and implementation, and it will take effect after being signed and approved by the sponsor and the designated laboratory of this project. transmission. The data administrator will conduct a consistency check, and the data with problems will be notified to the sponsor by email. After each party modifies the corresponding data or verifies that the data is correct, it will be retransmitted until the relevant data is consistent.

Medical coding: The data management unit is responsible for the medical coding of this study. Coded using the version of MedDRA specified in the project DMP.

Blind review: After data cleaning, question answering, and database closure (before locking), relevant parties (funders, researchers, data administrators, statisticians) will perform data clarification on the database and biological samples before unblinding The results are reviewed to form a population classification decision.

Locking of the database: After the data is confirmed to be correct through a blind review, the database is locked with written approval, and approved by the co-signature (signature and date) of the unblinding relevant personnel (statistician, researcher, sponsor). The unlocking and re-locking of the database can only be carried out with

the written consent of the above-mentioned personnel, and records should be made. After the database is locked, the data manager needs to submit the locked database to the statistician for statistical analysis.

Data management report: The data steward writes the data management report after unblinding.

11.8. Statistical analysis

11.8.1. Analysis Sets

- **Analysis set of efficacy**

Full analysis set of efficacy(E-FAS): Include all subjects who followed the intention-to-treat (ITT) principle, entered randomization and completed vaccination, and had at least one case surveillance follow-up after vaccination.

Modified full analysis set of efficacy(E-mFAS): It is a subset of E-FAS and includes all subjects who completed vaccination, had negative baseline nucleic acid results, and had at least 1 case surveillance follow-up 14 days after vaccination. Among them, COVID-19 cases with positive nucleic acid or antigen test or confirmed diagnosis within 14 days from enrollment to vaccination will also be excluded from this analysis set.

Per protocol set of efficacy(E-PPS): It is a subset of E-mFAS, including all subjects who did not violate the inclusion/exclusion criteria, completed vaccination, had negative baseline nucleic acid results, and had at least one case surveillance follow-up 14 days after vaccination, and did not violate the protocol. Among them, COVID-19 cases with positive nucleic acid or antigen test or confirmed diagnosis within 14 days from enrollment to vaccination will also be excluded from this analysis set.

Cases of E-mFAS and E-PPS are counted from 14 days after vaccination and are

mainly used for the evaluation of the main protective efficacy of the vaccine. Among them, E-mFAS is the main analysis set for the evaluation of protective efficacy in this study. E-FAS was used to evaluate the protective efficacy after vaccination.

➢ **Analysis set of immunogenicity**

Full analysis set of immunogenicity(I-FAS): Include all subjects in the immunization subgroup who follow the ITT principle, enter randomization and complete vaccination, and have valid antibody data before vaccination.

Per protocol set of immunogenicity(I-PPS): Include all subjects in the immunization subgroup who did not violate the inclusion/exclusion criteria, completed the vaccination, had blood samples collected at the evaluation time points, and had valid antibody data. The immunogenicity analysis sets were defined for the immunogenicity evaluation of each group at 7 days, 14 days, and 28 days after vaccination respectively.

Analysis set of immune persistence(IPS): Include subjects whose blood samples were collected at all immune persistence evaluation time points and have valid antibody data. The immune persistence analysis set was defined for the immunogenicity evaluation at 3 and 6 months post-vaccination, respectively.

I-FAS and I-PPS were used for immunogenicity analysis. IPS was used for immune persistence analysis.

➢ **Analysis set of safety**

Safety analysis set(SS): Include all subjects vaccinated with the study vaccine.

All the above analysis sets will be discussed and decided by the main investigators, funders, statisticians, and data managers in a blinded data review meeting before the database is locked.

11.8.2. Statistical Analysis Methods

11.8.2.1. General principles

Measurement data will be described statistically with mean, median, standard deviation, maximum value, and minimum value; counting data or grade data will be expressed with frequency.

All statistical analysis will be performed using the statistical software SAS EG 8.3.

11.8.2.2. Subject distribution, demographic information, and baseline characteristics

The study screening, the number of subjects enrolled in each group, the number of subjects who completed the study, and the number of cases in each analysis set were summarized, and the dropout subjects and reasons for dropout were analyzed. The demographic characteristics of the subjects in each group were statistically described.

11.8.2.3. Protection effectiveness analysis

The person-year incidence rates and 95% confidence interval(CI) of COVID-19 confirmed by the central laboratory with clinical symptoms will be calculated among the test group and the control group 14 days after the vaccination, and the Poisson regression model will be used to analyze the statistical differences between the groups. VE and 95% CI based on the person-year incidence rates will be estimated with the model. The Poisson regression model takes the number of patients as the dependent variable, grouping as the fixed effect, and the exposure person-years of the participants as the offset, using the log connection function.

Person-year incidence rate=(number of cases)/(exposure person-years of the participants)×100%. In the calculation of the exposure person-years, the starting time is the 14th day after the vaccination, and the ending time is the day when a COVID-19 case was first confirmed. If the RT-PCR or antigen test was positive but not confirmed as a COVID-19 case, the person-year incidence rate was calculated using the RT-PCR or antigen test date as the termination time. VE rate=1- (person-year incidence rate of the test group)/(person-year incidence rate of the control group).

The VE will be evaluated in the prevention of clinically symptomatic COVID-19 confirmed by central laboratory virology 14 days after vaccination based on the sets of E-mFAS and E-PPS. The evaluation of VE based on E-mFAS is the main analysis result of this study.

In addition, the VE of the vaccine against clinically symptomatic COVID-19 confirmed by central laboratory virology 7 days after vaccination will be evaluated based on E-mFAS, and the statistical method is the same as the primary efficacy endpoint.

11.8.2.4. Immunogenicity analysis

The positive conversion rates of SARS-CoV-2 neutralizing antibodies in the test group and the control group will be calculated respectively in the immunization subgroup, and the Clopper-Pearson method will be used to calculate the 95% CI. The chi-square test/Fisher's precision probability test will be used to compare the statistical differences in the positive conversion rates between the test group and the control group.

The GMT and GMI of SARS-CoV-2 neutralizing antibodies in the test group and the control group in the immunogenicity subgroup will be statistically described with geometric mean and two-sided 95% CI. The paired t-test of the logarithmically transformed results will be used to perform statistical tests on the difference between the test group and the control group.

The degree inverse distribution graph of the antibody titers of anti-SARS-CoV-2 neutralizing antibody and IgG antibody in the test group and control group in the immunogenicity subgroup will be plotted before immunization and 7 days, 14 days, 28 days, 3 months, and 6 months after the vaccination.

11.8.2.5. Safety Analysis

MedDRA will be used for the medical coding of adverse events and serious adverse events, classified and counted according to the SOC and PT. The solicited adverse events for the reactogenic subgroup will be classified and counted according to the inoculation site and non-inoculation site (systemic) adverse events specified in the protocol. TEAE will be mainly analyzed in this study and the adverse events that occurred before vaccination will be listed in the form of a list. Unless otherwise specified, the following adverse events are TEAEs.

The frequency, number, and incidence of all adverse events and AESIs, adverse events and AESIs related to the study vaccine, adverse events and AESIs unrelated to the study vaccine, adverse events and AESIs of grade 3 or above, and adverse events and AESIs of grade 3 or above related to the study vaccine will be calculated respectively in the test group and control group. Fisher's precision probability test will be used to compare the differences between groups in the incidence of the above adverse events. The occurrence time and severity of adverse events will be statistically described. The adverse events and AESIs related to the study vaccine or unrelated to the study vaccine will be listed respectively.

The frequency, number, and incidence of all serious adverse events, serious adverse events related to the study vaccine, and serious adverse events not related to the study vaccine will be calculated respectively in the test group and control group. Fisher's precision probability test will be used to compare the differences between groups in the incidence of the above adverse events. The serious adverse events will be listed.

11.8.3. Interim Analysis

Not applicable.

11.8.4. Handling of missing data

In the statistical analysis of I-FAS, the last observation carried forward (LOCF) method will be used to carry forward the missing data of antibody results after vaccination, and derive the corresponding immunogenicity endpoints. Missing data in the safety evaluation endpoint and immune persistence endpoint will not be processed

in this study. Details on the treatment of missing data are provided in the Statistical Analysis Plan.

11.8.5. Multiplicity Analysis

Not applicable.

12. Subject Safety and adverse event management

12.1.Adverse Event Definition

Adverse Event (AE): it refers to all adverse medical events that occur after participants receive the vaccine/drug for the study, which can be manifested as symptoms, signs, diseases, or laboratory test abnormalities, but are not necessarily related to the study vaccine/drug.

Adverse Vaccine Reaction (AVR): it refers to any harmful or unexpected reactions that may occur in clinical trials and be related to the study vaccine. There is at least a reasonable possibility of a causal relationship between the study vaccine and the AE, i.e., the correlation cannot be ruled out.

Serious Adverse Event (SAE): it refers to the adverse medical event that causes death, is life-threatening, leads to permanent or serious disability or incapability, requires hospitalization or prolonged hospitalization, and results in congenital abnormalities or birth defects after the participants receive the study drug/vaccine.

Advent Events of Special Interesting (AESI): refers to the adverse event of special scientific and medical interest to a product or project, which may or may not be a serious event. The investigator should be able to monitor them on an ongoing basis and report them promptly to the sponsor. For applicable AE, the investigator should investigate further to describe and understand them. Depending on the nature of the event, the sponsor may also need to communicate and/or report it to other interested parties.

Suspected Unexpected Serious Adverse Reaction (SUSAR): it refers to suspected unexpected serious adverse reactions whose clinical manifestations are beyond the

Investigator's Brochure for the study drug/vaccine, package inserts for marketed drugs/vaccines, summary of product characteristics, and other existing information in terms of their nature and severity.
Adverse Events Following Immunization (AEFI): It refers to the reaction or event suspected to be related to vaccination after vaccination.

12.2. Security precaution

The first-aid facilities and equipment in the first-aid room are effective, the first-aid drugs are within the validity period, and the first-aid doctors have corresponding qualifications and capabilities. When the participant has an adverse event at the study site, he/she will be treated in the on-site emergency room on time, and follow-up hospitalization will be arranged according to the arrangement. During the enrollment of the participants, the agreement department shall be notified to prepare for timely treatment. A strict SOP is required to specify the responsibilities of the investigator, the contact telephone number of the investigator, the route of the ambulance, etc. to ensure that the unexpected adverse events can be dealt with on time. When the participant needs to be hospitalized for emergency treatment after serious adverse events, the agreed department can provide green channel services such as medical treatment, hospitalization, and medical security to ensure that the participant can be treated in time.
The sponsor shall designate full-time personnel to be responsible for the safety information monitoring and SAE reporting management of the clinical study. Both the sponsor and the investigator should develop standard operating procedures for safety information monitoring and SAE reporting in the clinical study, and train all relevant personnel. The monitoring and reporting of adverse events in vaccine clinical study shall be completed jointly by participants, adverse event investigators, and investigators at different observation time points in stages.

12.3. Discovery and Collection of Adverse Events

During the training of participants, it should be emphasized that adverse events need to be reported promptly, and investigators should be highly alert to such events and investigate and handle them promptly.

For solicited AEs and unsolicited AEs, ask the participants if they have received hospitalization and outpatient treatment or taken drugs, and record this information.

When a serious adverse event occurs, the investigator is responsible for reviewing all documents related to the event (e.g.: hospital course, medical order records, laboratory reports, and diagnostic reports), or to clarify the nature and relevance of SAEs, the investigator arranges clinical examinations/tests as required. If a participant is confirmed dead during study participation or the follow-up period, the hospital's conclusion on the deceased person should be collected, and a copy of the results, including histopathology results, should be obtained if an autopsy is performed.

The investigator should try to collect complete copies of medical records, but cannot replace study records with copies of medical records of participants. All information related to serious adverse events should be recorded on the original records and serious adverse event report pages.

If medical records are to be released for medical evaluation purposes, all fields identifying the participant should be masked before release.

12.4. Treatment and Management of Adverse Events

If AE occurs in the participants in the study, the investigator and specialist should closely monitor the changes of AE in the participants and give corresponding treatment if necessary.

The investigator should establish an emergency plan for SAE handling in the clinical study, train all relevant personnel, and take measures to know any clinically significant disease/event after the vaccination of subjects. By the relevant national regulations and current medical management practices, the participants will be promptly sent to the designated hospital for appropriate treatment. Medications used to treat AEs should be recorded in the participant's source records and EDC.

In case of disagreement and dispute in the handling of adverse events, the investigator shall be obliged to cooperate with the sponsor in handling and assisting the participants in medical identification.

The sponsor has the obligation and responsibility to guarantee the safety of the participants unconditionally. For the confirmed adverse reactions related to the vaccine, the sponsor shall compensate the participants under the requirements of the Vaccine Administration Law of the People's Republic of China, the Regulations on the Administration of Vaccine Circulation and Vaccination, and the Measures for Compensation of Abnormal Reactions to Vaccination (Trial) of each province.

The investigator should pay continuous attention to the adverse events that persist due to study termination or the end of the visit. The adverse events related to vaccination should be followed up until the end of the event. The follow-up can be stopped after the unrelated events are diagnosed by the doctor.

12.5. Serious Adverse Event Reporting

12.5.1. Reporting Procedure

Investigators should fill out the initial report of the Serious Adverse Event Report

Form within 24 hours after learning SAE and report it to the sponsor and CRO on behalf of the sponsor by e-mail. After the initial report, the investigator should continue to follow up the SAE and report new information or changes to the previous report, event outcomes, etc. in the form of follow-up/summary reports, to pay attention to the serious adverse events that have not recovered/have not recovered or are recovering/are recovering until recovery/condition is stable, and complete the summary report. If the participant is diagnosed with COVID-19 and meets SAE standards, they may not report SAE; If at this point, the combined underlying disease worsens and meets SAE standards, then the exacerbation of the underlying disease needs to be reported as SAE. For reports involving fatalities, the investigator should provide the sponsor and the IRB/EC or other parties as required with any additional information requested, such as autopsy reports and final medical reports.

Start/end dates of AE/SAE

(1) AE/SAE start date: for a new disease, the date of appearance of first relevant signs/symptoms is defined as the AE/SAE start date; for previous diseases (defined as the medical history before enrollment and still present at vaccination), the date of first exacerbation of signs/symptoms is used as the AE/SAE start date, not the date of participant reporting or investigator learning of them; if the AE/SAE is an abnormal laboratory test after vaccination with clinical significance, the start date is recorded as the sampling date.

AE/SAE end date: for a new disease, the last day that the relevant signs/symptoms lasted is defined as the AE/SAE end date; for the AE/SAE or abnormal laboratory test value caused by exacerbation of previous diseases, the date when the signs and symptoms return to the baseline level or the state is better than the baseline level, and the investigator evaluates that the event has been stable, is defined as the AE/SAE end date; if the AE/SAE is an abnormal laboratory test after vaccination with clinical significance, the end date is the sampling date that the result return to normal.

It should be noted that the start/end date of SAE resulting in

hospitalization/prolongation of hospitalization is also based on the above judgment rules, with the date of appearance of first relevant signs/symptoms as the start date of SAE, rather than the date of hospitalization/prolongation of hospitalization (unless the date of hospitalization/prolongation of hospitalization is the same as the date of appearance of first relevant signs/symptoms); the last day that the relevant signs/symptoms lasted is defined as the SAE end date, rather than the discharge date (unless the discharge date is the same as the last day of relevant signs/symptoms presence); The end date of SAE resulting in death is defined as the date of death. When recording AEs in the eCRF, use accepted medical terminology whenever possible. To improve the quality and accuracy of AE information collection, the investigator should follow the following guidelines.

- AE records are recorded in eCRF using accepted medical terminology whenever possible. Record and report SAEs.
- Document the diagnosis (i.e., the disease or syndrome) rather than the associated signs, symptoms, and laboratory findings (e.g., document congestive heart failure rather than dyspnea, rales, and cyanosis).
- Document and report SAEs that result in death.
- For patients hospitalized for a surgical or diagnostic procedure, the disease that led to the surgical or diagnostic procedure should be documented as an SAE, not the procedure itself. The process should be documented in the measures for disease treatment section of the case narrative.
- Pregnancies that occur in participants during the study period are not considered AEs per se but should be recorded on a separate pregnancy record form, and the pregnancy report should be sent to the sponsor and the sponsor’s designated CRO within 24 hours. if the outcome of the pregnancy meets SAE criteria (including spontaneous abortion, stillbirth, or any congenital anomalies, etc.), the investigator should report it according to the SAE reporting process.

The investigator should sign off and read the relevant safety information provided by the sponsor for the clinical study promptly and consider the participant's treatment,

whether to adjust it accordingly, communicate with the participant as early as possible if necessary, and report suspected and unintended SAE provided by the sponsor to the IRB/EC or other parties if needed.

After being informed of the SAE report, the sponsor is required to conduct a comprehensive analysis and evaluation of the SAE as soon as possible; the initial report is required to be submitted to all investigators participating in the clinical study, the clinical study institution, the IRB/EC, the drug regulatory authority, and the health care authority in accordance according to the following timelines:

(1) For SUSAR that are fatal or life-threatening, the sponsor shall report them as soon as possible after the initial notification, but not later than 7 calendar days, and report the relevant follow-up information within the following 8 calendar days.

(2) For SUSAR that are not fatal or life-threatening, the sponsor shall report them as soon as possible after first becoming aware of them, but no later than 15 calendar days.

(3) For other potentially serious safety risk information, the sponsor shall report it as soon as possible after first becoming aware of it, but no later than 15 calendar days.

The Sponsor has a duty and responsibility to ensure the safety of participants, to provide humane care to participants who experience AE during participation in clinical studies, and to treat and compensate participants whose AE is determined to be related to vaccination.

12.5.2. Report content

- Type of report and reporting time;
- Participant information;
- Reporter information;
- Information on investigational vaccines;

- Study-related information;
- Information on co-morbidities and treatment;
- SAE details;
- Time the investigator was informed;
- Investigator signature.

12.6. Reporting of AESI

All AESIs, whether serious AE or not, and whether causally related to the investigational vaccine or not, need to be recorded in an AESI Report Form within 24 hours after being informed and sent to the sponsor and delegated CRO.

AESIs in this study included:

- Immune system: exacerbation of disease following immunization;
- Respiratory system: acute respiratory distress syndrome;
- Cardiovascular system: acute coronary syndrome, intracranial aneurysm, arrhythmia, vascular endothelial dysfunction, cardiac failure, myocarditis, pericarditis, myocardial infarction, sudden death, microangiopathy and stress cardiomyopathy;
- Hematologic disorders: coagulation disorders, idiopathic thrombocytopenic purpura, pulmonary embolism, cerebral hemorrhage, limb ischemia, thrombocytopenia, thromboembolism;
- Nervous system: acute disseminated encephalomyelitis, Guillain-Barre syndrome, general convulsion, meningoencephalitis, peripheral neuropathy, multiple sclerosis, transverse myelitis and other demyelination lesions, neuritis optica, neuromyelitis, optic nerve spectrum disorders, myasthenia gravis, Bell's palsy;
- Skin manifestations: pernio-like lesions, cutaneous vasculitis, erythema multiforme.

13. Quality assurance and control of Clinical research

13.1.Investigator

Standard operating procedures should be developed before the study is initiated, and the SOP strictly to ensure quality.

The primary investigator and clinical research quality control specialist shall be appointed before the initiation of the clinical trial by the research site.

All clinical research staff shall be designated by the primary investigator and accept appropriate training to understand their respective duties before joining the clinical work.

The vaccine clinical research center should carry out quality control activities promptly, check the work of all links to find problems and risks in time, and follow up on the problems until they are closed.

The research site of vaccine clinical research qualifies preventive vaccination approved by the Administrative Department of Health and Family Planning, has relatively fixed and sufficient clinical research researchers, is equipped with standard operating procedures related to vaccine clinical research, conducts training, and has training records. According to the vaccination and visit process of vaccine clinical research, there are a reception area, informed consent room, consultation and physical examination screening room, biological specimen collection room, vaccination room, first aid room, medical observation room, vaccine storage room, archive room, sample

processing and preservation room and temporary storage place for medical wastes, etc., and the research site is equipped with relevant ambulance personnel and first-aid items to ensure the safety of the subjects during the vaccination and observation period safety.

13.2.Funder

Funders have the ultimate responsibility for the quality of clinical studies. It shall establish a perfect quality management system for vaccine clinical research, formulate corresponding SOPs, organize audits of clinical research, and conduct systematic inspections of activities and documents related to clinical research, including research centers, laboratories, CRO companies, etc., to evaluate whether the research is carried out per the requirements of the research protocols and SOPs and whether the data of the research are recorded in a timely, truthful, accurate and complete manner.

The funder shall conduct a comprehensive on-site assessment of the clinical research centers and determine the research centers based on the assessment results according to the implementation conditions and requirements of the clinical research before the study is carried out.

The funder shall organize the supervision of the clinical research and assign a sufficient number of supervisors to carry out the whole supervision of the clinical research.

The funder shall designate a full-time staff to be responsible for the monitoring of clinical study safety information and the management of SAE reports, to keep abreast of the latest status of the safety information of the whole clinical study, and to inform all the participating investigators and regulatory authorities on time.

Participate in the investigation and treatment of adverse reactions and adverse events,

and be responsible for providing medical treatment or related compensation costs for cases of adverse reactions and clinically proven vaccine-related adverse events by relevant regulations.

13.3.Site Monitor

The number of monitors should be sufficient to meet the needs of the work, and the supervisors should have educational background and working experience in medicine, pharmacy, or related professions.

The on-site supervisors are responsible for supervising and inspecting the whole process of clinical research to ensure that the clinical research process meets the requirements of the clinical research program. The supervisor assumes the following responsibilities:

(1) Confirming before the study that the research undertaking unit has appropriate conditions, including staffing and training, complete and well-functioning laboratory equipment, and various conditions related to the study.

(2) Supervisors should provide appropriate guidance and training to the researchers involved in the site, and instruct the researchers to familiarize themselves with the research protocol and the operational procedures of each link.

(3) Verify that the qualifications and authorizations of the researchers in the study meet the requirements.

(4) Supervise the implementation of the study protocol and SOPs by the researcher during the study.

(5) Verify that informed consent was obtained from all subjects before the study and that enrolled subjects were qualified.

(6) Understand the progress status of the study and report to the funder and the investigator.

(7) Ensure that all study information on subjects is recorded accurately and timely in a complete and standardized manner on the original documents and that EDC entries are correct and consistent with the original data. All errors or questions were corrected or clarified.

(8) Confirm that all adverse events are documented and that serious adverse events are reported and documented within the required time frame.

(9) Ensure that all protocol violations, protocol deviations, and other events are recorded in a timely and accurate manner and reported as required.

(10) Verify that research vaccines are supplied, stored, distributed, used, and withdrawn according to SOP requirements and that records are made accordingly.

(11) Verify that biological samples are collected and stored by the protocol and relevant SOPs and that records are made accordingly.

(12) Assist the researcher in necessary notification and application matters, and report study data and results to the sponsor.

Regularly arrive at the study site to conduct monitoring and submit reports on the monitoring work to the funder and the unit responsible for the clinical study.

13.4.Blood sample management

(1) Blood sample collection: the process of blood sample collection should be by the provisions of its SOP, the collection of blood samples should be recorded, and the blood collection tube should be easy to identify and have a unique identification.

(2) Serum separation: the subject's blood sample is fully coagulated, and the serum should be separated by centrifugation in time (within 4 hours) and recorded. The separated serum is divided into delivery and backup serum. Serum tubes should have easily recognizable, unique, and traceable markings.

(3) Serum cold chain management: serum samples should be managed by specialized personnel, and serum samples should be established to keep file accounts. Send

serum and backup serum -20℃ or below low-temperature preservation. The administrator of blood samples should check the storage temperature of serum on time and record it according to the SOP of blood sample management or related systems to avoid the occurrence of over-temperature. If over-temperature occurs, it should be reported to the sponsor in time. Serum storage and transportation during temperatures> -20 ℃ and <0 ℃ is cold chain abnormality, and≥ 0 ℃ is cold chain damage.

(4) Serum delivery: before delivery, the research party needs to organize the serum for delivery according to the requirements of the testing agency, and the temperature during serum transportation needs to be kept at ≤ -20℃.

(5) Preservation and treatment of backup serum: the backup serum should be properly preserved by the researcher and managed according to the SOP of blood sample management or related system, and the research center may not handle it before the funders give their opinions on the treatment.

13.5.Vaccine management

(1) Vaccine supply: Funders should provide investigators with vaccines that are easily identifiable and correctly coded and labeled "for clinical research only".

(2) Vaccine delivery: The whole process of vaccine management should comply with the requirements of the cold chain, and the vaccine transportation and preservation conditions should be according to the requirements of the program. The vaccine delivery process should have complete transportation and temperature monitoring records.

(3) Handover of vaccines: Vaccine administrators need to verify the vaccine lot number, quantity, expiration date, and delivery status and fill in the vaccine handover records promptly when administering vaccines.

(4) Cold chain management of vaccines: Vaccines should be managed in special cabinets under lock and key, and research vaccines should be placed in

independent partitions with clear markings. Research vaccines should be placed at -20°C±5°C for storage before thawing. Vaccine administrators should set up vaccine entry and exit accounts check the storage temperature of vaccines on time according to the vaccine management SOP or related systems and make records to avoid over-temperature. If over-temperature occurs, the investigator should initiate countermeasures and report to the funder in time.

(5) Distribution and use of vaccines: The distribution and use of vaccines should be recorded in detail, and the vaccination process should be traceable, including the time of subject assignment, the assignee, and the information of the vaccine assigned for vaccination, such as the number, etc. The temperature of vaccines should be recorded by the requirements of their SOPs. The investigator should ensure that the vaccines are all administered within the vaccine potency period and that they are not used in non-study populations.

(6) Vaccine recovery and counting: Vaccine administrators should recover the remaining vaccines promptly, conduct regular counts, and keep records of the counts to ensure that the number of vaccines used and remaining matches the total number of vaccines.

(7) Disposal of surplus vaccines: Discarded, expired, and surplus vaccines should be kept by relevant SOPs or requirements, and may not be destroyed on-site before the disposal advice is given by the funders.

(8) Recycling of empty vaccine boxes and vials: empty vaccine boxes and vials after vaccine use should be managed by specialized personnel for timely recycling sorting and counting, and a management account for empty vaccine boxes and vials to ensure that the number of empty boxes and vials is in line with the number of vaccines used and that they cannot be destroyed at the site of the study before the funders give their opinions on their treatment.

13.6.Research data

The data that recorded the real information of the subjects, such as the informed

consent form, were sealed in the research center;
Archive management shall be carried out according to the SOP and or room management system, and a corresponding ledger shall be established with security measures such as insect prevention, moisture-proof, fire prevention, anti-theft, and anti-rodent.

13.7.Publication of research results

After the completion of the study, the research unit may, with the written consent and authorization of the funder, introduce, publish and publicize the methods and results of the clinical study in seminars, national or regional professional conferences, or in journals, papers, and academic lectures in a manner agreed to by the sponsor, provided that it does not infringe the sponsor's proprietary rights and intellectual property rights to the methods, results, data, reports, information, documents, etc., of the clinical study. ownership and intellectual property rights. Negative or inconclusive findings should be published or publicized in the same way as positive findings.

14. Clinical Medical Research Ethics Committee

14.1. Ethical standards

The study will be conducted by the GCP, World Medical Association Declaration of Helsinki, and all applicable regulations. The study protocol, each study center-specific ICF, participant training and recruitment materials, and other required documentation (including any subsequent modifications) will be reviewed and approved by the IRB overseeing the study site.

14.2. Ethical review

The site investigator will be responsible for ensuring that this protocol, ICF, and other study-related documents have been reviewed and approved by the relevant local IRB before implementation. Any revisions to the protocol, ICF, or other study-related

documents must be approved by the IRB before implementation. A copy of the protocol, written information about the ICF or other participants, and any proposed recruitment materials should be submitted to and approved in writing by the site IRB. If necessary, the investigator must submit all subsequent protocol amendments and changes to the ICF and obtain IRB approval. The investigator will notify the IRB in writing of SAE and protocol deviation noted in the protocol as required by the local regulatory authority and the IRB. The study will be conducted in full compliance with the protocol.

The protocol will not be amended without prior written approval by the sponsor. All protocol amendments must be submitted to and approved by relevant IRB(s) before implementing the amendment at each site.

15. Confidentiality

The subject's personal information is not disclosed during research conducting, biological sample collection and reporting, or publishing. Only the investigator has limited access to electronic or hard-copy information.

The founder, investigator, and clinical medical research ethics or the regulatory agency have access to research-related data. However, the data can't be used in any other clinical study and can't be disclosed to any other person or entity.

The investigator must sign off the confidentiality agreement to ensure they understand the confidentiality of clinical research information. The investigator and other research staff shall keep confidential all information provided by the founder and all the data/information generated during the clinical study, not including the subject's medical record. All the above information can't be used for any other purpose except clinical research. This restriction does not apply to :

(1) Study information is not published due to violations by the investigator and/or the research staff.

(2) Disclosure of research information to clinical medical research ethics only for research evaluation.

(3) Disclosure of research information to provide appropriate medical assistance to subjects.

(4) Publication of research results authorized by the founder.

If the written contract for this study involves a confidentiality clause that contradicts this statement, the contractual clause shall prevail.

Appendix 1.

1. Main efficacy endpoint COVID-19 case definition and diagnostic criteria

Refer to the National Health Commission of the People's Republic of China. Diagnosis and Treatment Protocol for COVID-19 Pneumonia (Trial Version 10, 05Jan2023)(during the study, the latest version of the guidelines shall prevail). The primary efficacy endpoint of clinical study, COVID-19 cases, is defined as subjects with positive SARS-CoV-2 nucleic acid or antigen test with clinical symptoms of any severity, and the diagnostic criteria are as follows:

The subjects developed any of the following signs or symptoms:

A. dry throat/sore throat 1

B. fever (oral temperature 37.5 ° C/axillary temperature ≥37.3 ° C)(lasting any time) or chills

C. muscle pain

D. new onset olfactory/gustatory abnormalities 1

E. nasal congestion/runny nose 1

F. diarrhoea

G. conjunctivitis

H. general fatigue/fatigue 1

I. headache

J. anorexia/nausea/vomiting 1

Or

Subjects developed one or more of the following respiratory symptoms:

A. cough (lasting any time)

B. breathe is in shortness of breath or has difficulty breathing (at any time)

Or

Subjects with clinical or radiographic evidence of pneumonia [2];

And

The subject tested positive for SARS-CoV-2 nucleic acid or antigen.

1: Several signs separated by a slash (/) should be considered as one sign

2: Typical chest imaging findings of COVID-19 include the following:

In the early stage, multiple small patchy shadows and interstitial changes were found, especially in the outer lung. Then develops into bilateral lung multiple ground-glass shadow, infiltration shadow, serious cases can appear lung consolidation, pleural effusion rare. In MIS-C, cardiac insufficiency patients can see cardiac shadow enlargement and pulmonary edema.

1. Secondary efficacy endpoint severe/critical COVID-19 case definition and diagnostic criteria

Severe/critical COVID-19 cases are part of all severity COVID-19 cases. Subjects

meet the primary efficacy endpoint COVID-19 case definition and diagnostic criteria. Severity grading investigators and CEC experts will assess severe/critical COVID-19 cases according to the classification criteria of the COVID-19 Pneumonia Treatment Protocol (Trial Version 10) issued by NMPA.

2. Diagnosis and Treatment Protocol for COVID-19 (Trial Version 10) issued by NMPA

diagnostic criteria:

1. Clinical manifestations associated with SARS-CoV-2 infection

2. The results of etiological and serological tests were as follows:

(1) the detection of nucleic acid was positive;

(2) the detection of antigen was positive;

(3) the isolation and culture of SARS-CoV-2 was positive;

(4) the level of specific IgG antibodies to SARS-CoV-2 in the convalescent stage was 4 times higher than that in the acute stage.

Clinical types:

(I) mild

upper respiratory tract infection as the main manifestations, such as dry throat, sore throat, cough, fever, and so on.

(II) moderate

persistent high fever > 3 days or (and) cough, shortness of breath, but respiratory rate (RR) < 30/min, breathing at rest means oxygen saturation > 93%. The imaging findings were characteristic of pneumonia caused by COVID-19 virus infection.

(III) severe

adult patients met any of the following criteria and could not be explained by any other reason other than SARS-CoV-2 infection:

(1) shortness of breath, RR ≥30/min;

(2) at rest, when inhaling air, oxygen saturation ≤93% ;

(3) Pao2/FIO2≤300 mmHg (1 mmHg = 0.133 kPa) , PAO2/Fio2 should be corrected according to the following formula: PAO2/Fio2 × [760/mmHg] .

(4) the clinical symptoms were gradually aggravated, and the lung imaging showed that the lesion progressed more than 50% in 24 ~ 48 hours.

(IV) critical

the patients with critical type had one of the following conditions:

(1) respiratory failure and required mechanical ventilation;

(2) shock;

(3) complicated with other organ failure required intensive care unit (ICU).

16. References

[1] . Novel Coronavirus Pneumonia Diagnosis and Treatment Program (Trial 10th Edition).

[2] Technical Guidelines for the Development of mRNA Vaccines for Novel Coronavirus Prophylaxis (Trial).

[3] Norms for Preventive Vaccination Work (2016 Edition): National Health and Family Planning Commission, December 6, 2016.

[4] Guiding Principles on Adverse Event Grading Criteria for Clinical Trials of Vaccines for Prophylaxis, State Drug Administration, [2019] No. 102, December 31, 2019.

[5] Yang Huan et al, Considerations for the design and implementation of safety studies in clinical trials of vaccines for prophylaxis, Chinese Journal of Preventive Medicine, March 2020, Vol. 54, No. 3.

[6] Zhang Yanling et al, Vaccinology (6th edition), Science and Technology Press 2017.

[7] Coronavirus disease (COVID-19) pandemic. https://covid19.who.int/

[8] https://www.who.int/publications/m/item/draft-landscape-of-covid-19-candidate-vaccines

[9] Yanping Zhang. Epidemiologic characterization of novel coronavirus pneumonia[J]. Chinese Journal of Epidemiology, 2020, 41(2): 145-151.

[10] Li Q. An Outbreak of NCIP (2019-nCoV) Infection in China Wuhan, Hubei Province, 2019-2020. China CDC Weekly. 2020, 2(5): 79-80.

[11] Chan JF, Yuan S, Kok KH, et al. A familial cluster of pneumonia associated with the 2019 novel coronavirus indicating person-to-person transmission: a study of a family cluster Lancet 2020. DOI: 10.1016/ S0140-6736(20)30154-9 [published Online First: 2020/01/28].

[12] Rothe C, Schunk M, Sothmann P, et al. Transmission of 2019-nCoV Infection from an Asymptomatic Contact in Germany [J]. NEJM 2020. DOI: 10.1056/NEJMc2001468 [published Online First: 2020/02/01].
